# Interrogating bioinspired ESIPT/PCET-based Ir(iii)-complexes as organelle-targeted phototherapeutics: a redox-catalysis under hypoxia to evoke synergistic ferroptosis/apoptosis†

**DOI:** 10.1039/d3sc03096b

**Published:** 2023-08-29

**Authors:** Maniklal Shee, Dan Zhang, Moumita Banerjee, Samrat Roy, Bipul Pal, Anakuthil Anoop, Youyong Yuan, N. D. Pradeep Singh

**Affiliations:** a Department of Chemistry, Indian Institute of Technology Kharagpur West Bengal–721302 India ndpradeep@chem.iitkgp.ac.in anoop@chem.iitkgp.ac.in; b School of Biomedical Sciences and Engineering, South China University of Technology, Guangzhou International Campus Guangzhou 511442 PR China yuanyy@scut.edu.cn; c Department of Physics, Indian Institute of Science Education and Research Kolkata Mohanpur West Bengal 741246 India

## Abstract

Installing proton-coupled electron transfer (PCET) in Ir-complexes is indeed a newly explored phenomenon, offering high quantum efficiency and tunable photophysics; however, the prospects for its application in various fields, including interrogating biological systems, are quite open and exciting. Herein, we developed various organelle-targeted Ir(iii)-complexes by leveraging the photoinduced PCET process to see the opportunities in phototherapeutic application and investigate the underlying mechanisms of action (MOAs). We diversified the ligands' nature and also incorporated a H-bonded benzimidazole-phenol (BIP) moiety with π-conjugated ancillary ligands in Ir(iii) to study the excited-state intramolecular proton transfer (ESIPT) process for tuning dual emission bands and to tempt excited-state PCET. These visible or two-photon-NIR light activatable Ir-catalysts generate reactive hydroxyl radicals (˙OH) and simultaneously oxidize electron donating biomolecules (1,4-dihydronicotinamide adenine dinucleotide or glutathione) to disrupt redox homeostasis, downregulate the GPX4 enzyme, and amplify oxidative stress and lipid peroxide (LPO) accumulation. Our homogeneous photocatalytic platform efficiently triggers organelle dysfunction mediated by a Fenton-like pathway with spatiotemporal control upon illumination to evoke ferroptosis poised with the synergistic action of apoptosis in a hypoxic environment leading to cell death. Ir2 is the most efficient photochemotherapy agent among others, which provided profound cytophototoxicity to 4T1 and MCF-7 cancerous cells and inhibited solid hypoxic tumor growth *in vitro* and *in vivo*.

## Abstract 2

We investigated organelle-targeted bioinspired ESIPT/PCET-based Ir(iii)-complexes as promising (photo)chemotherapeutic lead compounds which actively operational in redox-catalysis under hypoxia to evoke synergistic ferroptosis/apoptosis.

## Graphical abstract

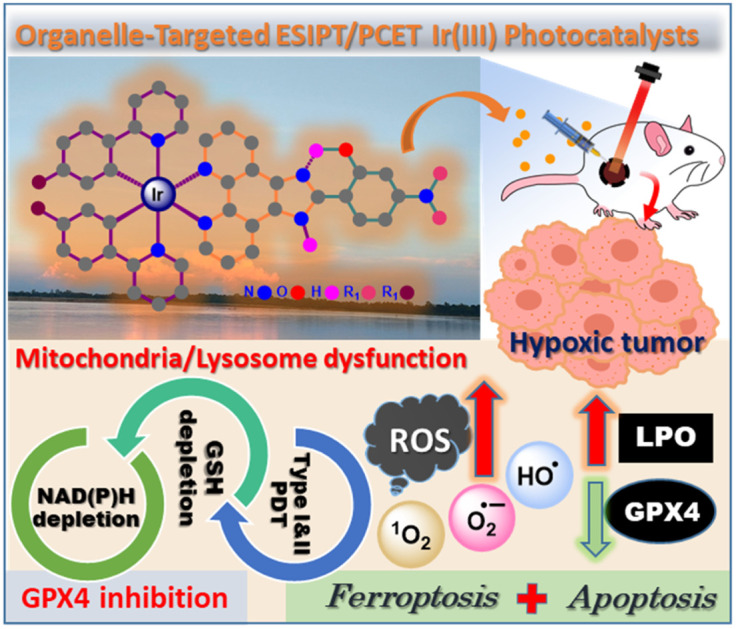

## Introduction

PCET is the discerning application of redox photochemistry advanced by nature where transportation of electrons (ET) occurs from one site to another accompanied by proton transfer (PT) during reactions.^1–3^ This mechanistically interesting phenomenon plays a pivotal role in providing low over-potential redox reaction pathways, production of bioenergetics, DNA repair, and other applications.^4–6^ The essential PCET mediated redox-relay process operates in a sequence of short-distance and rapid redox-equivalent transfer steps to form a charge separation (CS) state at catalytic sites, *e.g.*, the H-bonded tyrosine–histidine (TyrZ–His190) pair presented in Photosystem II (PSII).^2,3^ Indeed, all living cells fundamentally carry out the transportation of e^−^ and H^+^ in a specific direction under electrochemical potential gradients, including the generation of proton-motive force (PMF) for providing energy to maintain living systems.^7^ The PCET mechanism is responsible for reversibly translating chemical potential energy from redox processes to PMF or in an inverse way.

Molecules composed of a H-bonded BIP platform as an e^−^ transfer mediator, which is a closer biomimetic model for the TyrZ–His190 pair, feature the PCET process.^7^ Recently, this essential process has been employed in metal-complexes utilizing the BIP model attached to a chelating phenanthroline group, which serves as an anchoring site for Ru(ii) or Re(i) to develop various photoelectric devices and improve photocatalytic reaction efficiency (*e.g.*, water splitting).^8^ Indeed, introducing PCET in metal-complexes provides interesting photophysics and optimal photochemical output by impeding charge recombination.^9^ However, an in-depth understanding of the PCET mechanism in complexes is still in progress. Nowadays, cyclometalated Ir(iii)-complexes have become much popular in various disciplines, especially owing to their usage as effective photocatalysts and phototherapeutic drugs, *etc.*, because of their synthetic versatility, tunable photophysical properties, and excellent photochemical stability.^10–12^ Intriguingly, despite several advantages of PCET chemistry, introducing it in Ir-complexes, studying their excited-state dynamics, and photoinduced PCET are scarcely explored.^13,14^ A PCET-based Ir-photocatalyst was introduced with enhanced photochemical quantum efficiency by taking advantage of PCET to limit the charge recombination (CR) step.^13^ Recent development of PCET in Ir(iii) in conjugation with ESIPT ligands was carried out to study the solvent mediated PCET dynamics and its mechanistic aspects.^14^ Hence, designing new PCET-based Ir(iii)-complexes featuring high photostabilities with long emission lifetimes and applying them to biological systems for understanding PCET dynamics is quite appealing.

Application of novel metal-based anticancer agents attracted the most attention to combat drug resistance and reduce side-effects apart from well-studied Pt-compounds.^15^ Recently, special attention has been paid to account for photoactivable metal-complexes (Ru-, Pt-, and Lu-based), which showed excellent potential for cancer treatment for their spatiotemporal control over drug activation.^16^ Indeed, cutting-edge catalytic photodynamic therapy (PDT), a complementary technique, offers high therapeutic efficacy at low concentrations and mostly overcomes drug resistance.^17–19^ However, therapeutic efficacy of PDT defies under hypoxia due to the complex microenvironment of hypoxic tumors, the operational detoxification mechanism by over expressive intracellular antioxidants (GSH), drug efflux by P-glycoproteins, and induction of stress response genes.^19*a*,20,21^ To overcome these circumstances, developing new generations of photosensitizers with novel mechanisms of action (MOAs) for cancer phototherapy and to participate in effective ET in competition with energy transfer (EnT) is needed. Specifically, rejuvenating PDT through the type-I method has the benefit of low O_2_ content dependency by the generation of cytotoxic radicals *via* SET to adjacent substrates and O_2_.^20,21^

Apoptosis and ferroptosis are two distinct types of programmed cell death with different activation mechanisms.^22,23^ The well-established apoptosis pathway mostly operates by the activation of caspases followed by the release of DNase to induce DNA fragmentation and subsequent nuclear changes.^22^ However, in the last decade, ferroptosis has become a promising therapeutic approach, characterized by the iron-dependent LPO accumulation in the cell membrane.^23^ This distinct type of cell death mechanism is triggered by the deactivation of the GPX4 enzyme directly to result in an imbalance of cellular redox homeostasis and inhibition of the enzymatic system responsible for removing LPOs from the cell membrane. Interestingly, cellular ferroptosis induced by nonferrous metal-ions is uncommon in the literature, offering a promising lead to circumvent hypoxia-induced resistance.^24^ Moreover, disruption of redox-balance by abating intracellular NADH in the mitochondrial electron transport chain (ETC) prompts cell degeneration even in hypoxia by stimulating ferroptosis.^25^ Therefore, NADH has become a prospectively sought-after target for cancer drug development.

**Fig. 1:**
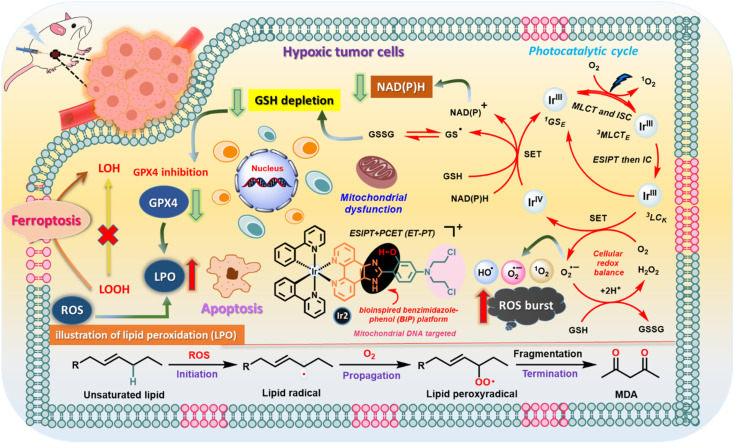
Schematic illustration of the photocatalytic pathways of the Ir2 complex in cancer cells based on the experimental results.

With the stimulation from unexplored aspects, herein, we strategically synthesized four different Ir(iii)-catalysts by introducing bioinspired PCET-based ancillary ligands as an intrinsic component for studying photophysical processes; moreover, we used photoinduced PCET to accentuate their potential application in biology as organelle-targeted (mitochondria and lysosome)^26^ phototherapeutic agents. The PCET strategy by introducing an electron acceptor BIP platform is used to amplify the type-I PDT reactions and O_2_ reduction reactions.^7*b*,27^ Interestingly, we found that all Ir-complexes have a relatively large two-photon absorption cross section (TPACS), good photostability, and profound *in vitro* (photo)cytotoxicity against 4T1 and MCF-7 cancer cells under hypoxia. Notably, mitochondrial targeted Ir2 bearing an *N*,*N*-bis(2-chloroethyl)-azane derivate^19*c*,28^ has the highest phototherapeutic efficiency, applied for the inhibition of solid hypoxic tumor growth in mice, which effectively photooxidizes endogenous NADH in living cancer cells and reduces molecular O_2_ to ROS, triggering ferroptosis adhesively with apoptosis (Fig. 1).

## Results and discussion

### Design plan

**Fig. 2:**
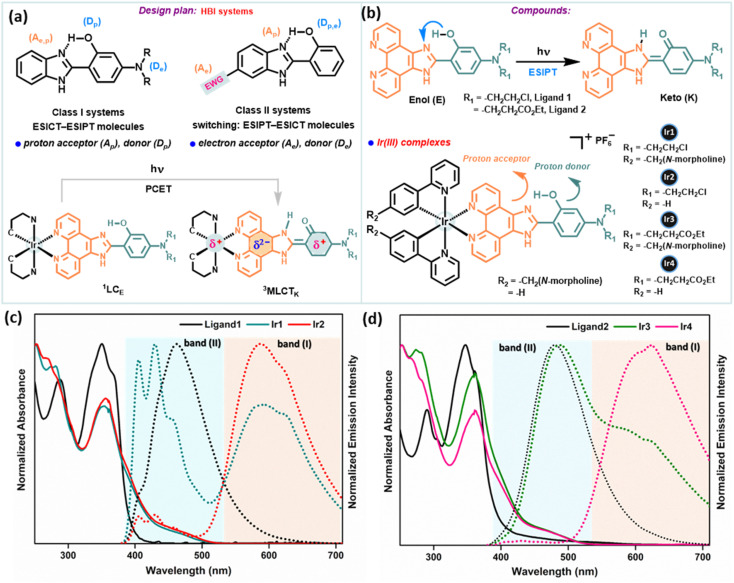
(a) Design plan for synthesizing desired compounds. (b) Structures of phenanthroline-BIP based ancillary ligands attributing to the ESIPT phenomenon and the corresponding Ir(iii)-complexes. The proton-accepting and donating parts are indicated in orange and cyan dark colors, respectively. (c) and (d) Normalized UV-vis absorption spectra (solid line) and the corresponding emission spectra (dotted line) of (c) ligand1, Ir1 and Ir2; (d) ligand2, Ir3 and Ir4; in DCM (10 μM), respectively under 365 nm excitation. Two emission bands are assigned as major band (I) and minor band (II), respectively.

PCET coupled with ESIPT/ESICT (excited state intramolecular charge transfer) processes received particular attention for systems invoking ESIPT, especially to avoid bimolecular complexity.^29^ In various electronically substituted 2-(2′-hydroxyphenyl)benzimidazole (HBI) molecules, the interplay between ESIPT and ESICT processes is apparent (depicted in Fig. 2a), which manifests interesting photophysical properties due to the simultaneous act of both events.^29,30^ In this approach, we have strategically adopted the intrinsic ESIPT of the parent HBI moiety to synthesize new phenanthroline-BIP based ancillary ligands with an intramolecular H-bonded –OH group to act as the proton donor (D_p_), while *N*,*N*-dialkyl amino groups at the C-4 position act as an e^−^ donor (D_e_), benzazole N(sp^2^)-group as a proton acceptor (A_p_), and phenanthroline as an e^−^ acceptor (A_e_) (Fig. 2b). Overall, our designed systems have a good electronic coupling matrix with a strong π-network of e^−^ donors and acceptors, where a facile ET reaction may simply involve instant e^−^ relocalization. To tempt excited-state intramolecular PCET, we have conjugated the chromophoric ligand with an Ir(iii)-atom, where the e^−^ accepting phenanthroline moiety is prompted to be more of an e^−^ sink (Fig. 2a).

### Synthesis and characterization

Two new phenanthroline-BIP based ancillary ligands (Fig. 2b) were synthesized following literature reports (Scheme S1†).^9^ These compounds adopt a rigid molecular structure with two aromatic rings in a coplanar geometry in the solution phase due to strong intramolecular H-bonding between the phenolic –OH group and N-atom of imidazole, a critical design parameter for effective intramolecular PCET reactions. This is supported by distinct structured UV-absorption bands ranging from 300–380 nm in various solvent polarity systems and the existence of dissymmetric protons of the phenanthroline ring in ^1^H NMR spectra (in DMSOd_6_) (Fig. 2c, d, S42 and 43†). Moreover, the absence of –OH group vibration characteristics in the IR spectrum also indicates intramolecular H-bonding interaction (Fig. S48†). We synthesized four desired cyclometalated Ir(iii)-complexes (denoted as Ir1, Ir2, Ir3, and Ir4; Fig. 2b) by varying the ligands (Scheme S2†). The intermediates and final products were characterized by ^1^H NMR, ^13^C NMR, electrospray ionization (ESI) high-resolution MS, IR, and UV-vis absorption spectroscopy (detailed in the ESI†), and their purity was checked by HPLC.

### Electronic absorption/emission spectra and emission lifetimes

**Fig. 3:**
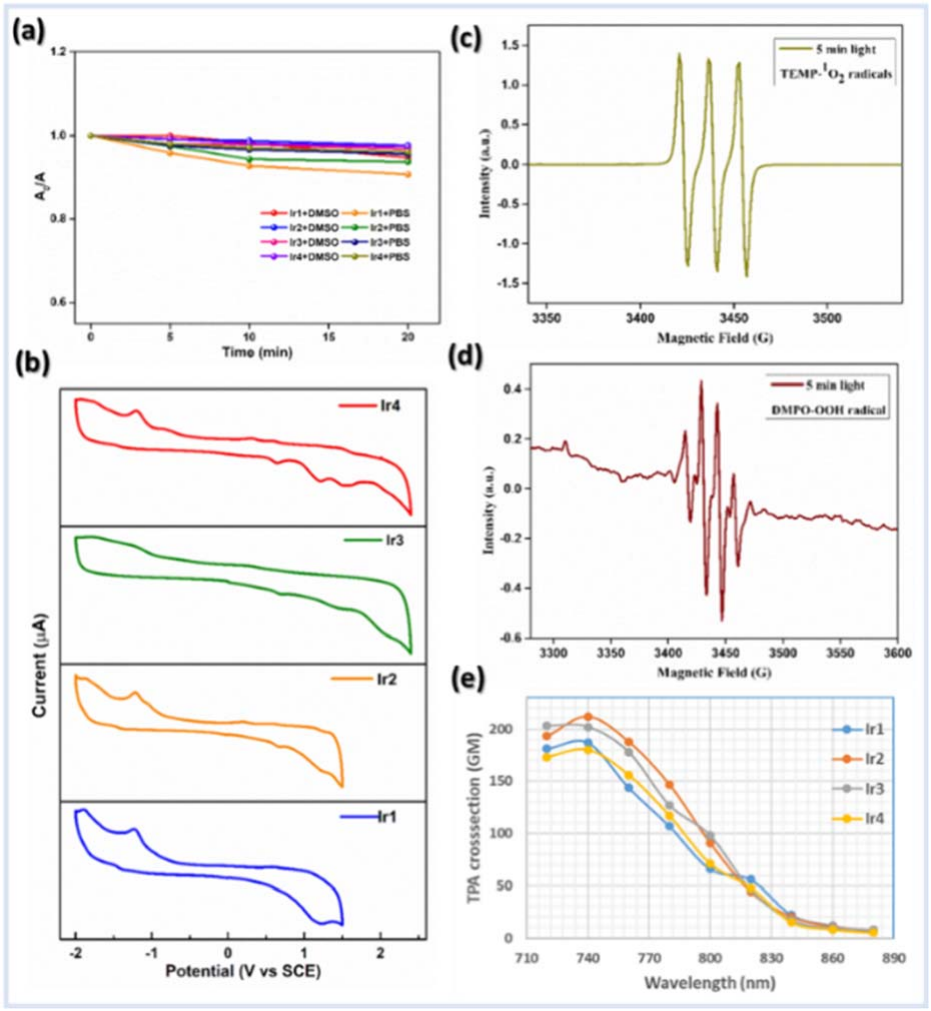
(a) Photostability study: normalized absorption spectra of Ir1–Ir4 (1 μM) in DMSO and PBS (1% DMSO) after irradiation with a 405 nm LED for different time intervals between 0 and 20 min. (b) Electrochemical properties of Ir1–Ir4 (0.5 mM) *vs.* SCE in dry CH_3_CN using 0.1 M nBu_4_NPF_6_ supporting electrolyte, a Ag/Ag^+^ reference electrode, a glassy carbon working electrode, and a Pt-wire counter electrode with a scan rate @100 mV s^−1^. (c) and (d) ESR spectra to detect (c) ^1^O_2_ using TEMP (10 mM) and (d) ˙OH using DMPO (10 mM) as spin trapping agents respectively in the presence of GSH (10 mM) under irradiation with light for 5 min. (e) Two-photon absorption cross-sections (*δ*_2PA_) of Ir1–Ir4 complexes in DMSO at different excitation wavelengths between 720 and 880 nm.

The steady-state absorption and emission spectra of Ir1–Ir4 were investigated in different aprotic to protic solvents with varying polarity indices, *e.g.*, DCM, ACN, DMSO, MeOH, and phosphate-buffered saline (PBS) solution for further application in biological studies at 298 K (Fig. S1–S5†). The absorption spectra of Ir1–Ir4 in DCM are similar to those of free ligands, except for some fine features with broadening over 400 nm (Fig. 2c and d). The absorption spectra consisting of intense absorption bands ∼300 nm are attributed to spin-allowed ligand-centered (^1^LC) π–π* transitions for ligands. The moderately intense absorption bands between 320 and 425 nm are assigned for the mixed transitions resulting from ligand-centered charge-transfer (^1^LCCT) and singlet metal-to-ligand charge-transfer [^1^MLCT, Ir^3+^(dπ) → ppy(π*)]. Less intense absorption bands >425 nm (an absorption tail reaching ∼500 nm) are due to the integrated transition of triplet ^3^MLCT and spin-forbidden ^3^LC. Delicate differences in the absorption spectra of Ir1–Ir4 are observed for PBS buffer. Furthermore, the absorption in DMSO and PBS (1% DMSO) in the dark and upon continuous irradiation with a 405 nm LED for 20 min was monitored in UV-spectroscopy. Promisingly, very few minor changes were observed, indicating high photostability of Ir1–Ir4 in the physiological environment and suitability for stable photosensitizers (Fig. 3a, S2 and 3†).

Upon excitation (*λ*_ex_ = 365 nm), Ir1–Ir4 exhibit blue to red emissions with unstructured and broad shaped bands in DCM solvent (Fig. 2c and d). The intense and broad emission band over 550 nm (I) with a small shoulder peak is typical of pronounced triplet ^3^MLCT/^3^LLCT transitions upon electronic excitations. However, another minor emission band (II) with low intensity at approximately 400–480 nm (except for Ir1, which has a more intense emission) can be assigned to fluorescence from the ^1^LC state. The short lifetime of emission band (II), evaluated using the time-correlated single-photon counting (TCSPC) method in the range of 1–4 ns (for instance, 1.47 ± 0.56 ns @450 nm for Ir1 or 4.01 ± 0.25 ns @490 nm for Ir3), suggests that this band corresponds to fluorescence (Fig. S6 and Table S1†). An additional indication on the origin of the emission band (II, 400–480 nm) was obtained from the comparison study with the emission spectrum of free ligands in the same region with the emission (II) of Ir-complexes (Fig. 2c and d). The emission decay profiles indicate that the lifetimes (*τ*_F_) of Ir2 and Ir4 are longer compared to those of Ir1 and Ir3 in DCM (Table S1†). The steady-state emission and corresponding quantum yields (*Φ*) of 0.01, 0.01, 0.031, and 0.011 were quantified for Ir1–Ir4 in DMSO, respectively (Table S2†). However, in polar protic solvents (MeOH and PBS), all complexes exhibit mild photoluminescence (emission quenching) in the red region.

We also recorded photoluminescence spectra and the corresponding luminescence lifetimes in a THF glassy matrix at 77 K (Fig. S7 and Table S3†) to get the essence of triplet states. The triplet lifetime (*τ*_T_) for each complex was also determined in different solvents by time-resolved phosphorescence measurements at 298 K (Table S4†). The formation of a long-lived triplet charge transfer state infers that Ir-complexes have good photocatalytic activity which can drive chemistry.

### Electrochemical properties

Cyclic voltammetry (CV) experiments of Ir1–Ir4 and free ligands were performed in degassed ACN solution to gain insight into the electrochemical parameters and investigate the feasibility of Ir-complexes in redox catalysis (Fig. 3b, S8 and 9†). The important electrochemical parameters are presented in Table S5.† The CV shows a first oxidation potential (*E*^a^_p_) at 0.63 and 0.61 V *vs.* SCE, respectively, for ligands 1 and 2, which corresponds to the process of phenoxyl radical formation from phenol, much lower values than those observed for H-bond free phenol.^7,9^ Hence, the first one-electron oxidation peak at 0.65–0.69 V *vs.* SCE for Ir1–Ir4 is assigned to the PhO˙/PhOH couple of the attached phenanthrolin-BIP ligands. Moreover, another irreversible oxidation peak in the range of 1.22–1.48 V *vs.* SCE was found for all Ir-complexes (Fig. S8†), which is likely to be assigned to the Ir(iv/iii) couple.

### Solvent-modulated ESIPT and PCET

To substantiate the ESIPT phenomenon, we studied the emission spectral change of Ir1–Ir4 and ligands using mixed solutions with various fractions of DCM (aprotic) and ethanol (polar protic solvent, to disturb the PT process), and the emission intensity variation curves are illustrated (Fig. S10–S12†). Ancillary ligands exist mainly in keto (K) form in the excited state. Overall, the change in relative peak intensities of emission bands decreases with the addition of EtOH. The decreased emission intensities of emission bands (I) for Ir1–Ir4 reflect a decrease in the MLCT efficiency. Accordingly, we can infer that the emission band (II) observed at 400–480 nm for Ir-complexes corresponds to the emissions from the singlet excited state of the keto (^1^LC_K_) and enol (^1^LC_E_) forms. Overall, these studies indicate that the ESIPT process efficiently occurs in Ir1–Ir4; moreover, MLCT and PT processes can be modulated by the external H-bonding network with solvent. These experimental results support the occurrence of both PT and MLCT in these complexes which particularly gives a clue that PCET is operational. Solvent-modulated PCET in Ir-complexes can occur through a stepwise PT-ET or ET-PT mechanism depending on the H-bonding ability with the solvent (Fig. S17†).^14^ The low emission quantum yield of photoexcited Ir-complexes indicates a heavy population of non-emissive excited states. The addition of trifluoroacetic acid (TFA) to these complexes increases emission intensity, contrary to the free ligands (Fig. S5†). In an acidic medium, the imidazole PCET proton acceptor is protonated, preventing intramolecular PCET. The increment of emission intensity of Ir-complexes upon acid addition suggests the PCET quenches the emissive ^3^MLCT state by BIP oxidation, resulting in a charge separated state.

The PCET process is also supported by infrared spectroelectrochemistry (IRSEC) and bulk electrolysis (BE) experiments (Fig. S13–16†). Upon electro-oxidation of Ir3 or Ir4, changes in absorption bands relative to the ground state at 1616 cm^−1^, 1636 cm^−1^ (coupling between C=N stretching and C=NH^+^ bending modes of protonated benzimidazole), and 3260 cm^−1^ (N–H stretching vibration) in the IRSEC difference spectra suggest intramolecular PT from the phenol moiety. In the BE experiment, the newly formed quasireversible oxidation peaks (with *E*^a^_p_ = 0.75 V and 1.26 V for Ir3; *E*^a^_p_ = 0.8 V and 1.17 V for Ir4; *E*^a^_p_ = 1.11 V for free ligand2) indicate the phenoxyl radical/phenol couple of the attached BIP moiety and driving force for ET in a PT-ET mechanism.^7,9^

### Computational studies

The ground-state (GS) geometric conformations of Ir1–Ir4 were optimized using density functional theory (DFT) at the B3LYP/def2svp level of theory. Electronically excited structures were computed using time-dependent (TD)-DFT calculations with the CAM-B3LYP/def2-SVP functional (ESI†). The optimized structures and corresponding relative energy levels were calculated (Fig. S32–S38†). The GS consists of Ir(iii) in pseudo-octahedral geometry with ancillary ligands having a phenanthroline-benzimidazole-phenol moiety adopting an almost coplanar conformation. The dihedral angle (*θ*(CCCN)) between proton-donating and accepting groups in the optimized GS structure of Ir1–Ir4 was determined to be confined within a narrow range of (0.1–3.3)°. This limited angular deviation signifies a quasiplanar geometry. Furthermore, the distinctive alignment of the –OH group directed towards the N(sp^2^)-atom of the imidazole ring also facilitates strong intramolecular H-bonding interactions. The energy gaps between the lowest-energy excited states S_1_ and T_1_ (Δ*E*_ST_) of Ir1–Ir4 were determined to be 0.41, 0.40, 0.48, and 0.47 eV, respectively (Fig. S35†). However, S_1_ is also accessible to closely lying T_2_ or T_3_ states for favorable and fast intersystem crossing (ISC) due to large Ir-atom spin–orbit coupling (SOC). After rapid internal conversion, the T_1_ excited state is populated to participate in photochemical reactions. The charge distribution in charge density difference (CDD) plots of the enol-form of Ir-complexes showed distinct charge separation to form a CT state, with negative charge densities residing mostly on the phenanthroline moiety which corresponds to either ^3^MLCT or ^3^LLCT (Fig. S36†). The CT in the transient excited state is probably the driving force of the PT process or *vice versa*. Similarly, significant spin density was confined to the ESIPT ligand from the spin density plots (Fig. S37†), highlighting the interplay between CT and spin dynamics in the system.

### ROS detection in solution

The ability of the Ir-complex to catalytically generate ^1^O_2_ upon irradiation with a 405 nm blue LED was quantified using 1,3-diphenylisobenzofuran (DPBF) as an ^1^O_2_ trapping agent upon time-dependent monitoring of its concomitant decrease in absorbance @425 nm. Quantification of the amount of ^1^O_2_ produced in PBS buffer solution (1% DMSO) upon irradiation demonstrated that Ir1 and Ir3 were found to generate ^1O^_2_ slightly more efficiently than Ir2 and Ir4 (^1^O_2_ quantum yield of Ir1: 0.43, Ir2: 0.36, Ir3: 0.39, and Ir4: 0.33) (Fig. S18, 19 and Table S6†). However, Ir2 and Ir4 generated ˙OH radicals more efficiently due to their significantly longer luminescence lifetimes than Ir1 and Ir3. Probably, Ir2 and Ir4 showed a more efficient PCET process compared to Ir1 and Ir3 making the ET process faster.

As an endogenous bio-reductive molecule, GSH can scavenge generated ROS and limit the therapeutic effect. Afterward, the potential generation of various ROS by photocatalysis upon the addition of GSH was also qualitatively investigated by electron spin resonance (ESR) spectroscopy to prove the mechanism (Fig. S20†).

The characteristic 1 : 1 : 1 strong signal peak corresponding to (2,2,6,6-tetramethylpiperidin-1-yl)oxyl (TEMPO) in ESR spectra confirms the ^1^O_2_ production using standard scavenger 2,2,6,6-tetramethylpiperidine (TEMP) (Fig. 3c). Moreover, the ˙OH radical formation was assessed through a distinct ESR signal intensity ratio of 1 : 2 : 2 : 1 using 5,5-dimethyl-1-pyrroline N-oxide (DMPO) as a spin trap (Fig. 3d). Notably, the intensity of ESR signals for ˙OH was significantly improved from 0–5 min irradiation time (Fig. S20†). We speculate that the inclusion of PCET-based ligands in Ir(iii) may facilitate SOC and thus enhance ISC as well as stabilize the CT state to minimize the back ET process, which results in higher photocatalytic efficiency.^13^ The methylene blue (MB) assay is another detection technique used to further verify the photocatalytic efficacy of Ir2 for generating ˙OH. The MB and ˙OH reaction is photooxidation, resulting in a decolorization from blue to colorless (Fig. S21†).^31^ The conspicuous degradation of MB, as indicated by the lowering in absorbance @554 nm, was observed for PBS solution (pH = 7.4, 1% DMSO) of Ir2, and also for Ir2 pretreated with GSH, validating the photocatalytic formation of ˙OH. Overall, the above results indicate the ability of Ir-complexes to promote ROS generation *via* a dual type I/II photosensitization process.

### Two-photon absorption

Importantly, efficient photocatalytic drugs with excitation in the NIR-region are needed to treat solid tumors, which can be achieved using light through two-photon absorption (TPA). Evidently, several d^6^-transition metal complexes feature substantial optical nonlinearity.^32^ Hence, the two-photon luminescence of Ir-complexes was measured in DMSO solvent using a femtosecond ultrafast spectrometer equipped with an NIR two-photon laser. Ir1–Ir4 have strong TPA in the 720–840 nm region (Fig. 3e and S22†) and the absorption maximum was detected @740 nm with TPACS (*δ*_2PA_) values of 187 GM @ Ir1, 212 GM @ Ir2, 202 GM @ Ir3, and 180 GM @ Ir4 (Göppert-Mayer, 1 GM = 10^−50^ cm^4^ s^−1^ per photon).^32*b*^ The NIR two-photon excitation of Ir(iii)-complexes would allow for biological applications within the biological spectral window (600–900 nm), indicating their ability to treat large or deep-seated tumors through precise control of excitation sites. We have also corroborated the effective ROS generation ability of Ir-complexes through EPR studies using a two-photon laser @740 nm.

### *In vitro* study

#### Intracellular uptake and subcellular localization

**Fig. 4:**
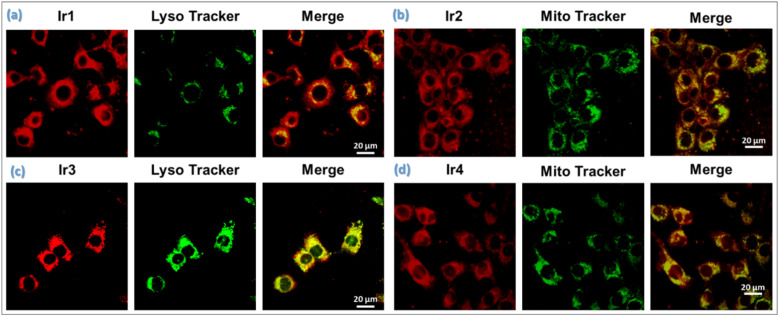
Cellular colocalization assay of Ir-complexes (a) Ir1 and (c) Ir3 with LysoTracker Green (LTG); (b) Ir2, and (d) Ir4 with MitoTracker Green (MTG) in 4T1 cells. The cells were incubated with Ir(iii)-complexes (2 μM, 37 °C, 4 h) (*λ*_ex_: 405 nm, red emission), followed by staining with mitochondria-specific dye, MTG (20 μM, 37 °C, 30 min) (*λ*_ex_/*λ*_em_ = 488/550 nm, green) or lysosome-specific dye, LTG (20 μM, 37 °C, 30 min) (*λ*_ex_/*λ*_em_ = 488/550 nm, green). Scale bar: 20 μm.

The cellular uptake profile of Ir1–Ir4 in 4T1 cancer cells was monitored by confocal laser scanning microscopy (CLSM) before examining the *in vitro* therapeutic effect. Red intracellular emission was observed in 4T1 cells after 4 h of incubation with compounds, attributing it to successful internalization of Ir-complexes. Then, specific subcellular localization of Ir1–Ir4 was studied by the colocalization assay using CLSM with the standard lysosome-specific dye (LTG) and the mitochondria-specific dye (MTG). The microscopy images expressed high congruency with good overlap with the MTG dye, indicating that Ir2 and Ir4 complexes primarily accumulate in mitochondria due to high nitrogen in both cyclometalated and ancillary ligands (Fig. 4b and d). However, Ir1 and Ir3 having morpholine as a lysosome targeting linker in the cyclometalated ligands substantially change subcellular localization to the lysosome. The red emission of Ir1 and Ir3 complexes exhibited good overlap with a commercial LTG dye, implying that Ir1 and Ir3 may selectively accumulate in the lysosome after entering the cells (Fig. 4a and c). These results indicate effective cellular uptake for further therapeutic applications.

#### (Photo-)cytotoxicity *in vitro*

**Fig. 5:**
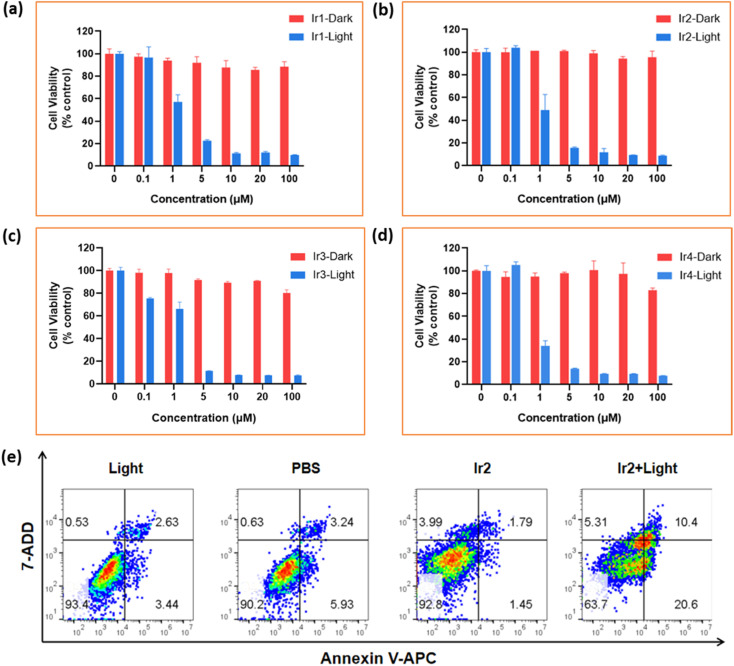
Identification of phototoxicity effects from Ir(iii)-complexes. (a)–(d) Relative cell viabilities of 4T1 cells in the presence of different concentrations of Ir1–Ir4 complexes after incubation at 37 °C for 24 h in the dark and under irradiation conditions at low concentrations of oxygen, respectively. All the values are presented as mean ± SD. (e) Flow cytometry analysis in 4T1 cells under hypoxic conditions using an Annexin V-APC and 7-AAD based method. The cells were treated with Ir2 (2 μM, at 37 °C) in an incubator for 4 h before irradiation. Cells were irradiated with 405 nm light, 20 mW cm^−2^, 10 min.

Based on these promising properties, *in vitro* (photo)cytotoxicity performance of all highly oxidative Ir1–Ir4 complexes was assessed against malignant mouse breast cancer cells MCF-7 and 4T1 under normoxia (21% O_2_) and hypoxia (1% O_2_) using standard 3-(4′,5′-dimethylthiazol-2′-yl)-2,5-diphenyl tetrazolium bromide (MTT) assay (Fig. 5a–d, S29–31† and Table 1). Satisfactorily, all Ir-complexes showed very low dark cytotoxicity with a half-maximal inhibitory concentration (IC_50_) value > 100 μM in both normoxic and hypoxic environments, and MB as a typical type-II PDT agent showed much higher dark toxicity (IC_50_ = 19–56 μM) (Table 1). Conversely, light irradiation led to a substantial increment in cytotoxicity of Ir-complexes against cancerous cells with an IC_50_ estimated in the range of 0.5–3.0 μM. The low dark-cytotoxicity and high (photo)cytotoxicity of these compounds led to a high (photo)cytotoxicity index (PI = IC_50(dark)_/IC_50(light)_). The PI index, recognized as one of the key parameters to evaluate the validity and safety of a promising clinical tumor treatment PDT agent, was calculated to be in the range of (0.32–1.3) × 10^2^. PIs of MB under normoxia and hypoxia are much lower than those of Ir-complexes. These results indicate the potential of our synthesized Ir-complexes as very effective phototherapeutic agents compared to many well-known PDT agents (*e.g.* 5-aminolevulinic acid, 5-ALA).^33^ Overall, Ir2 containing an *N*,*N*-bis(2-chloroethyl)-azane derivate was found to have an excellent therapeutic efficiency with high photocytotoxicity (IC_50_ = 0.5–1.40 μM) upon light exposure due to its mitochondrial accumulation, high ROS production efficiency and photooxidation of biomolecules *via* SET even in hypoxia, demonstrating an appreciable synergistic therapeutic effect. Importantly, Ir2 is the most efficient photochemotherapy agent among others and showed cytophototoxicity to 4T1 and MCF-7 cancerous cells even in hypoxic environments, an observation that supports our proposed cytotoxic mechanism.

**Table 1:** The dark- and photo-IC_50_ values of the compounds against MCF-7 and 4T1 cells under normoxia and hypoxia

Cells	Conditions	Hypoxic conditions (1%)					Normoxic condition (21%)				
	Compounds	Ir1	Ir2	Ir3	Ir4	MB	Ir1	Ir2	Ir3	Ir4	MB
MCF-7	Dark (μM)	>100	>100	>100	>100	43.89	>100	>100	>100	>100	19.09
	Light^a^ (μM)	1.73	0.78	1.06	1.17	8.55	2.06	1.40	2.01	2.78	5.28
	PI^b^	57.80	128.20	94.34	85.47	5.13	48.54	71.43	49.75	35.97	3.62
4T1	Dark (μM)	>100	>100	>100	>100	55.68	>100	>100	>100	>100	26.99
	Light^a^ (μM)	2.87	1.4	3.16	2.78	7.02	1.44	1.03	1.14	1.37	3.74
	PI^b^	34.87	71.43	31.64	35.94	7.93	69.59	97.56	87.87	73.1	7.22

a Photoirradiation was imposed (405 nm, 20 mW cm^−2^, 10 min) after 8 h of the incubation of complexes and then 40 h of recovery.

b Photo-cytotoxicity index, the ratio of (IC_50_)_dark_/(IC_50_)_Light_.

#### Flow cytometry assay

Generally, Ir-complexes exhibit the capacity to induce apoptosis under normoxia because of ROS generation. The PDT effect of Ir2 was assayed by flow cytometry analysis in 4T1 cells under hypoxic conditions with short time photoirradiation using an Annexin V-APC/7-AAD based method (Fig. 5e) to indicate early- and late-stage cell apoptosis or necrosis, where Annexin V-APC and 7-AAD (7-aminoactinomycin D) were applied to stain live and dead cells in green and red fluorescence, respectively. Ir2 did not induce apoptosis in cells after incubation in the dark, which is consistent with the observations of the control experiments. Notably, the photoinduced apoptosis by Ir2 was about 31.0% under hypoxia as a fraction of early and late apoptosis among all dead cells (Fig. 5e). However, the high photocytotoxicity effect of Ir2 at low concentrations of O_2_ clearly indicates that other cell death pathways are involved in this phototherapy. We speculated that ferroptosis might be actively involved in the cell death process induced by Ir2, which can potentially consume GSH to GSSG *via* oxidation by the photocatalytic process and could further inhibit the expression of the glutathione peroxidase 4 (GPX4) enzyme. To verify this hypothesis, we have performed several intra- and extracellular experiments. The GSH depletion was detected using UV-vis and fluorescence quenching experiments.

#### Photocatalytic NADH oxidation

**Fig. 6:**
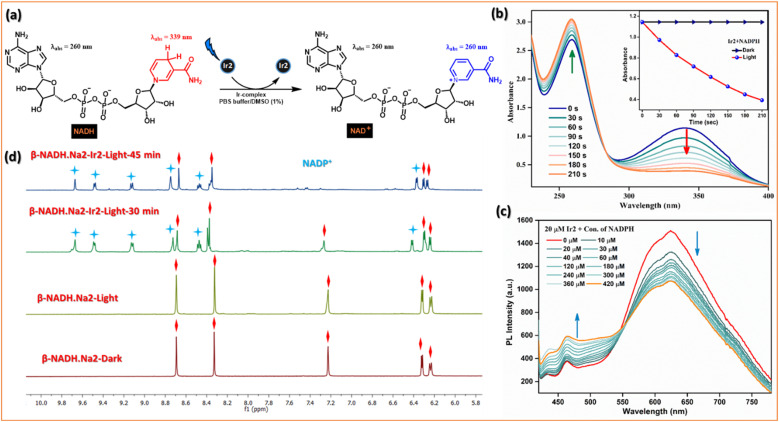
Photocatalytic oxidation of NADH. (a) Schematic illustration of photocatalytic NADH/NAD^+^ transformation by Ir2 under light irradiation. (b) Reaction of Ir2 (10 μM) and NADH (200 μM) in PBS solution under blue LED light irradiation monitored by UV-vis absorption spectra at 298 K. The inserted picture shows TON of Ir2 under dark or irradiation conditions. (c) Stern–Volmer quenching for the interaction of different conc. of NADH with the excited state of Ir2. (d) Photooxidation of NADH (3.5 mM) by Ir2 (0.25 mM) under dark or irradiation conditions monitored by ^1^H NMR spectroscopy at 298 K. Peaks associated with blue stars represent NAD^+^, and those with red squares represent NADH.

Mitochondria play an essential role in energy production, metabolism, and regulation of cell death, and they rely on the cofactor NADH to maintain redox balance, facilitate ATP synthesis, and regulate biomolecules.^25^ The depletion of intracellular NADH *via* oxidation to NAD^+^ in cancer cells would disturb the redox balance, leading to cell death. Hence, NADH has gained prominent importance as a target for cancer drug development. Since Ir-complexes have strong visible-light absorption and a long excited-state lifetime with high redox potentials, we studied their catalytic activity for the photooxidation of NADH and GSH in PBS (Fig. 6a and S23–28†). The high reduction potential of Ir(iii/iv), relative to GSH and NAD(P)H (*E*_red_ > −1.20 V *vs.* −0.24 V and −0.33 V respectively),^34^ favors the electron extraction from NADH or GSH by transient Ir^IV^-species generated in the photocatalytic pathway.

The photocatalytic efficiency of Ir2 (10 μM) towards NADH (200 μM) oxidation in PBS was first determined by UV-vis absorption spectroscopy. On photoirradiation (405 nm) in air, the absorbance of NADH @339 nm decreased, and absorbance @259 nm increased gradually with increasing irradiation time (Fig. 6b), and the conversion was also confirmed by HRMS. It follows first-order kinetics with a higher rate constant (*k* = 5.2 × 10^−3^ S^−1^) in comparison with Ir1, Ir3, and Ir4 (Fig. S24†). We evaluated the photocatalytic efficiency of the NADH oxidation reaction by calculating the turnover frequency (TOF) value which is quite high, *ca.* 310 h^−1^, compared to widely explored metallaphotocatalysts. Moreover, photoluminescence intensity of Ir2 decreased gradually when the NADH concentration was increased (Fig. 6c). The Stern–Volmer quenching experiments also validated the mechanism of photocatalytic oxidation of NADH. Furthermore, the photocatalytic oxidation of NADH (3.5 mM) by the Ir2-complex (0.25 mM) in D_2_O/CD_3_OD (1/3, v/v) at 298 K was monitored by ^1^H NMR spectroscopy (Fig. 6d and S25†). The newly formed peaks of NAD^+^ were observed at 6.27, 8.47, 8.71, 9.12, 9.48, and 9.67 ppm. In the dark, the sample remained unchanged for 24 h. This study indicates that Ir2 can reduce the enzyme activity of NADH under light irradiation.

### *In vitro* ferroptosis analysis *via* Fenton-type chemistry induced by Ir2 upon photoactivation

#### Accumulation of intracellular ROS and LPOs

**Fig. 7:**
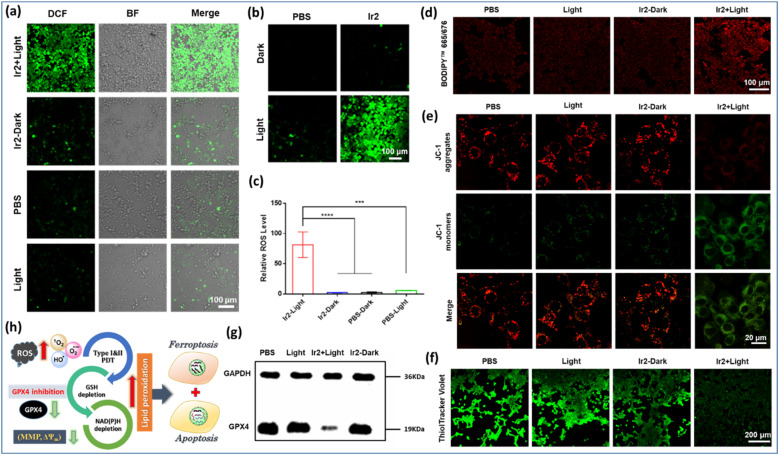
Ferroptosis induction by Ir2 under light irradiation in 4T1 cells. (a) and (b) CLSM images for intracellular ROS assessed by using a commercial DCFH-DA dye after various treatments in 4T1 cells under normoxia and hypoxia respectively. Scale bar: 100 μm. (c) Relative intracellular ROS levels under hypoxic conditions. (d) The fluorescence images of lipid peroxides in the treated 4T1 cells detected by the BODIPY^665/676^ probe. Scale bar: 100 μm. (e) (JC-1) assay in 4T1 cells after various treatments. Scale bar: 200 μm. (f) CLSM images for ThiolTracker Violet assay to quantify intracellular GSH levels after various treatments. Scale bar: 200 μm. (g) Image for western blot analysis of the GPX4 protein after various treatments. The relative expression levels of GPX4. Various treatments: (i) control experiment in the dark; (ii) control experiment with light (405 nm, 20 mW cm^−2^, 10 min); (iii) cells incubated with Ir2 (2 μM) for 4 h in the dark; (iv) cells incubated with Ir2 (2 μM) for 4 h and irradiated with light. Data are expressed as mean ± SD (*n* = 3), **p < 0.01, and ***p < 0.001. (h) The schematic illustration of a synergistic apoptosis/ferroptosis pathway.

The crucial indicator for ferroptosis in cancerous cells is the accumulation of LPO and intracellular ROS burst.^23,24^ Moreover, the central enzyme, GPX4 (selenoenzyme) is involved as a major ferroptosis suppressor through the reduction of LPO levels. To investigate this hypothesis, photocatalytic generation of intracellular ROS was assessed by using commercial 2′,7′-dichlorofluorescein diacetate (DCFH-DA) as the nonspecific ROS probe *via* CLSM images. The exposure of Ir2 to light (*t*_irr_ = 10 min) resulted in remarkably improved green fluorescence intensity from oxidized DCFH @529 nm under normoxia (Fig. 7a), corroborating the superior ROS generation capability of Ir2 for oxidative stress induced combined phototherapy, including under hypoxic conditions (Fig. 7b and c).

Furthermore, LPO levels were detected using an oxidation-sensitive and LPO-specific fluorescent probe, BODIPY^665/676^ dye, capable of accumulating at the cell membrane. The fluorescence of this probe changed when it reacted with ROO˙ radicals. The CLSM images revealed that the enhancement of red fluorescence in the case of ‘Ir2 + light’ treatment indicated the tremendous upregulation of LPO due to its reaction with the ROO˙ radicals (Fig. 7d). Generally, a typical LPO process involves three steps: (a) emerging ROS attacks polyunsaturated fatty acids to form lipid radicals at the initiation stage; (b) in the proliferation period, very unstable lipid peroxide radicals (ROO˙) are produced from lipid free radicals in combination with O_2_*via* the chain reaction to produce more of them; (c) finally, the termination process occurs through the breakdown of LOOH into smaller molecules forming malondialdehyde (MDA) as the by-product (Fig. 1). Glutathione consumption can down-regulate GPX4 activity, inhibit the lipid peroxidation protection system, and increase the production of LPOs, thus all together making tumor cells prone to ferroptosis, a rarely described form of cell death with clinical relevance due to its ability to overcome some of the limitations of traditional apoptosis-inducing therapeutic agents.

#### Mitochondrial depolarization

Mitochondrial membrane potential (MMP, Δ*Ψ*_m_) is an indicator of mitochondrial activity and plays a key role in ATP production, maintaining redox-balance, cell signalling, and cellular metabolism.^25^ Additional data for the oxidative damage by LPO were obtained with the 5,5′,6,6′-tetrachloro-1,1′,3,3′-tetraethylbenzimidazolyl-carbocyanine iodide (JC-1) assay in 4T1 cells using CLSM to characterize the change of MMP (Fig. 7e). The disappearance of fluorescence for J-aggregates in the red channel upon irradiation with Ir2 revealed drastic depolarization of the mitochondrial membrane (decrease in Δ*Ψ*_m_). Based on the selective localization of Ir2 in mitochondria (Fig. 4b), it induced a significant change in the Δ*Ψ*_m_ only after illumination as a result of photoinduced intracellular ROS generation, burst of LPO, and NAD(P)H photooxidation.

#### Intracellular GSH depletion and downregulation of GPX4 expression

Usually, overexpression of GSH in *in situ* tumor tissue maintains the redox-balance essential for proliferation by consuming ROS and activating GPX4 expression, which is a significant impediment to effective tumor therapeutics.^23,24^ Importantly, the inactivation of the GPX4 enzyme triggered by GSH depletion is often an important marker of the ferroptosis process. To visualize GSH depletion, we performed a ThiolTracker Violet assay to quantify intracellular GSH levels and general redox status in cultured 4T1 cells, incubated with various treatments for 4 h. The CLSM analysis depicted that ‘Ir2 + light’ treatment depressed the expression of GSH levels massively (Fig. 7f). The ˙OH generation might be promoted by the presence of GSH at millimolar levels. We further assessed the expression of GPX4 by a western blotting (WB) assay in 4T1 cells after similar treatments. The results showed that the ‘Ir2 + light’ treated cells could significantly downregulate GPX4 expression compared to other groups (Fig. 7g).

#### Photocatalytic performances and the mechanism of Ir2-complexes

Accordingly, we proposed a plausible mechanism to explain the pathways of ˙OH production *via* single electron transfer (SET) (Fig. 1 and S26†). Ir2 functions as a strong excited-state reductant, as evident from the reduction of molecular O_2_ even in the absence of any electron donor.^7*b*,27^ Initially, the Ir(iii) photosensitizer undergoes to an excited triplet state (^3^Ir*) *via* simultaneous MLCT and ISC processes. Then, ESIPT followed by the IC process forms the ^3^LC state, which rapidly provides an electron to O_2_ to generate the Ir(iv) electronic state. Now, strong oxidizing Ir(iv) abstracts 1e^−^ from substrates to turn-over the catalyst. We also speculated that Fenton-like reactions proceed for generating ˙OH from H_2_O_2_ under the action of ^3^Ir^(*n*)+*^ through EnT or ET processes (Fig. S26†).^35^ Overall, the cell death mechanism was also explained (Fig. 7h). The first amplified oxidative stress came from the basic type I/II PDT of Ir2. The oxidative stress burst was due to high reactivity of ˙OH with a high diffuse-controlled reaction rate. Drastic depletion of intracellular GSH levels could result in disrupted redox homeostasis to cause secondary oxidative stress. Generally, O_2_-independent PDT may work under slight or moderate hypoxia to produce sufficient ROS lethal to tumor cells but is inefficient under extreme hypoxia. In this situation, both ROS burst and GSH depletion strategies that are based on the oxidative mechanism have limitations. However, extreme hypoxia does not affect this PCET-based Ir-photosystem. In anaerobic solutions, Ir2 oxidizes NAD(P)H to NAD(P)^+^ and amino acids. Collectively, Ir2 manifested excellent phototherapeutic efficiency under hypoxia induced by distinct immunogenic apoptosis/ferroptosis cell death pathways.

### *In vivo* study: antitumor activity of Ir2

**Fig. 8:**
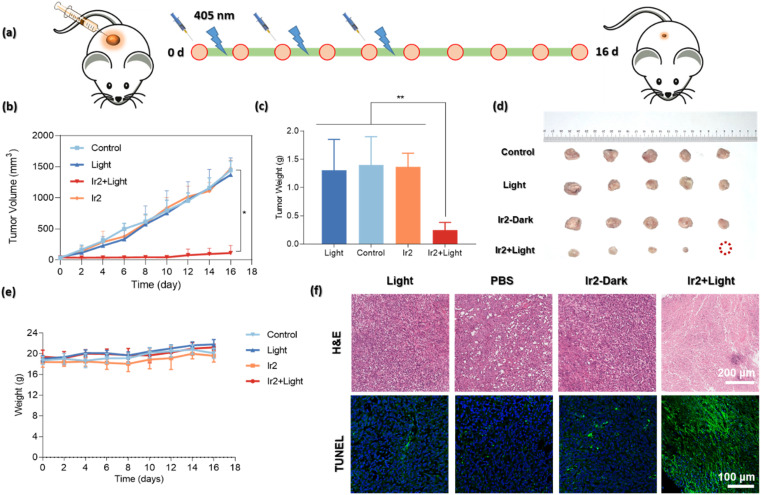
*In vivo* photo–therapeutic activity of Ir2. (a) Schematic illustration of the *in vivo* (4T1-tumor-bearing B/BLAC female mice) therapeutic protocol. Mice were irradiated using light (405 nm, 20 mW cm^−2^, 10 min) after intratumoral injection with 25 μL of PBS containing 500 μM of Ir2. (b) Tumor growth curves after various treatments. (*p < 0.05, **p ≤ 0.01 or ***p ≤ 0.001). (c) Tumor weights of mice at day 16 after various treatments. (d) Representative photographs of dissected tumors after various treatments [(i) the control group (PBS only), (ii) the PBS + light group, (iii) the only Ir2 (500 μM, 25 μL) (dark) group, and (iv) the Ir2 (500 μM, 25 μL) + light group, respectively; *n* = 5]. (e) Body weights of mouse groups after different treatments. (f) Representative H&E and TUNEL immunofluorescence staining images of the tumor tissues after different treatments. Scale bar: 200 μm and 100 μm, respectively.

The highly promising *in vitro* biocompatibility and photoactivated anticancer profile of Ir2 encouraged us to study its *in vivo* toxicity in 4T1-tumor-bearing B/BLAC female mice (Fig. 8a). Tumor growth inhibition in mice was evaluated when the primary tumor volume reached 50–80 mm^3^. The mice were randomly divided into 4 groups (5 mice/group): (i) the control group (PBS only), (ii) the PBS + 405 nm laser light (20 mW cm^−2^) group, (iii) the only Ir2 (dark) group, and (iv) the Ir2 + light group. Ir2 (500 μM, 25 μL) was administered to the mice by intratumoral injection. Tumor volume and body weight were recorded every 2 days for 16 days to note the therapeutic effect (Fig. 8b and c). The animals behaved normally without any signs of pain or stress during all treatments. However, the body weight of the mice did not change significantly, indicating that the systemic cytotoxicity of Ir2 is negligible (Fig. 8e). The results showed that there was no significant difference in tumor volume in the Ir2 without irradiation group. After exposure to light, the tumor volume of mice decreased significantly (nearly complete tumor eradication within a single treatment) compared with the other three groups, indicating that Ir2 has a photocatalytic role in the effective treatment of tumor *in vivo*. The photographic images of the dissected tumors visually highlight the strong antitumor effect (Fig. 8d).

After final treatment, the tumor tissues were collected for histological assessment to investigate the therapeutic effect. The hematoxylin and eosin (H&E) staining, as well as terminal deoxynucleotidyl transferase uridine triphosphate nick end labeling (TUNEL) staining, showed obvious destruction of tumor tissues for the treatment with the ‘Ir2 + light’ group, while tumor tissues in the other three groups were not affected (Fig. 8f). The observable strong green fluorescence signals for the ‘Ir2 + light’ group are the manifestation of DNA strand breaks during apoptosis. Overall, these results expressed the high biocompatibility and strong antitumor effect of Ir2 as a multifunctional therapeutic PDT agent.

## Conclusions

We have strategically developed different octahedral heteroleptic [Ir(iii)-phenanthroline-imidazole-phenol] complexes by introducing a H-bond interactive BIP platform, inspired by the PCET process in PSII mimicking the TyrZ–His190 pair to improve the photocatalytic efficiency. PCET limits the charge recombination process in catalytic pathways forming a charge separated triplet state supported by computational and spectroscopic studies. These complexes exhibited excellent photophysical and photochemical properties with a relatively large TPA cross section for photoexcitation in the NIR region (180–212 GM @740 nm), high photostabilities, long emission lifetimes, and dual emission properties in different H-boding solvent dynamics. We have also studied the effect of external H-bonding on the ESIPT/PCET dynamics of Ir-complexes in comparison with the largely conjugated π-extended mostly planar ESIPT ligands in various DCM/EtOH mixtures. We anticipated this by extending this PCET-based photocatalytic approach to a broad spectrum of targeted photoredox catalysis in cancer cells with high efficiency.

Ir1–Ir4 complexes are mostly localized in specific organelles (mitochondria or lysosomes) of cancer cells. Cell viability experiments indicate that these catalysts are equipotent towards normoxic and hypoxic environments, inducing remarkable photo-triggered cytotoxicity against MCF-7 and 4T1 cells, while remaining non-toxic in the dark (>100 μM). The manifestation of high photo-cytotoxicity of Ir2 was related to cumulative effects with its excess production of toxic ˙OH, photocatalytic oxidation of NADH, effective disruption of MMP (Δ*Ψ*_m_) and intracellular redox-balance, LPO accumulation, GSH depletion, and consequent GPX4 enzyme down-regulation, leading to a synergetic immunogenic ferroptosis/apoptosis. Furthermore, *in vivo* studies expressed good biocompatibility and photocatalytic anticancer efficiency of Ir2 in mouse cancer models, which could be a potential photochemotherapy lead compound.

This is an initial attempt, but our future outlook is to explore more of the ESIPT/PCET based Ir-complexes bearing the HaloTag ligand for further biological studies, such as by conjugating the thiol containing protein^4^ to quantify the hydration environment as a molecular probe based on the dual emissive nature and change in ratios of emission intensities affected by external H-bonding environments or map cellular trafficking of the glutathione conjugates produced within mitochondria.

## Ethical statement

All animal operations were in conformity with the Guidelines for Care and Use of Laboratory Animals of South China University of Technology (SCUT) and approved by the animal ethics of SCUT.

## Data availability

Data supporting this article have been uploaded as the ESI.†

## Author contributions

M. S. conceived and designed the project, synthesized Ir-complexes, investigated photophysical and photochemical properties, conducted other experiments, and wrote the full manuscript. N. D. P. S. supervised, validated all data, and helped in editing the manuscript. D. Z. and Y. Y. conducted the *in vitro* and *in vivo* studies and helped to analyse the corresponding data. M. B. performed the DFT calculations and A. A. supervised this study. S. R. and B. P. performed the two-photon study. N. D. P. S. and Y. Y. were responsible for the acquisition of funding and supervised the project. M. S. and D. Z. contributed equally.

## Conflicts of interest

There are no conflicts to declare.

## Supplementary Material

SC-014-D3SC03096B-s001
