# Supplementary material for: Interrogating bioinspired ESIPT/PCET-based Ir(iii)-complexes as organelle-targeted phototherapeutics: a redox-catalysis under hypoxia to evoke synergistic ferroptosis/apoptosis
Source: Chem Sci. 2023 Aug 29;14(36):9872–84. doi: 10.1039/d3sc03096b (PMC10510766; doi:10.1039/d3sc03096b)
Supplement: SC-014-D3SC03096B-s001 [file SC-014-D3SC03096B-s001.pdf]

Electronic Supplementary Material (ESI) for Chemical Science.
This journal is © The Royal Society of Chemistry 2023

Supporting Information

Interrogating Bioinspired ESIPT/PCET-Based Ir(III)-Complexes as Organelle-Targeted Phototherapeutics: A Redox-Catalysis under Hypoxia to Evoke Synergistic Ferroptosis/Apoptosis

Maniklal Shee,[a] Dan Zhang,[b] Moumita Banerjee,[a] Samrat Roy,[c] Bipul Pal,[c] Anakuthil Anoop*[a], Youyong Yuan*[b], and N. D. Pradeep Singh*[a]

[a]Department of Chemistry, Indian Institute of Technology, Kharagpur, West Bengal–721302, India.
E-mail: ndpradeep@chem.iitkgp.ac.in
E-mail: anoop@chem.iitkgp.ac.in

[b] School of Biomedical Sciences and Engineering, South China University of Technology, Guangzhou International Campus, Guangzhou 511442, PR China.
E-mail: yuanyy@scut.edu.cn

[c]Department of Physics, Indian Institute of Science Education and Research Kolkata, Mohanpur, West Bengal 741246, India.

Table of Contents

Table of contents……………………..………………...S2-S3

1.1. General information……………………..………………...S4

1.2. Synthesis and Structural Characterization.....…...S4-S10

1.3. UV-Visible Spectroscopy…….……...S11

1.4. Stability Tests ………………………………………………………..……….……..……...S12

1.5. Steady-State Emission Spectroscopy.…………………………………….……………S13-S14

1.6. Fluorescence Lifetimes ……………………………………….…………………….…….S15-S16

1.7. Fluorescence Quantum Yields ……………………………….…………………………S17

1.8. Phosphorescence spectra and lifetimes at 77 K…………………. …..……………..S18-S19

1.9. Triplet State Lifetimes ………………………...S20

1.10. Electrochemical measurements…………………………….….….……….….S21-S22

1.11. Solvent-Modulated ESIPT Dynamics ……………………………..………..S23-S24

1.12. Infrared Spectroelectrochemistry ……………………..……….……….…..S25- S26

1.13. Solvent-Modulated ESIPT and PCET Pathways ……..S27

1.14. Singlet Oxygen Quantum Yields…………..S28-S30

1.15. ESR measurements…………...S31

1.16. Methylene Blue Assay for Extracellular •OH Detection...S32

1.17. Two-photon Absorption Cross Section..S33-S34

1.18. Photocatalytic oxidation of NADPH by UV-visible spectroscopy........................S35-S36

1.19. Photocatalytic oxidation of NADPH by 1H NMR spectra..S37

11.20. Photocatalytic Performances and Mechanism of Ir2-complex …..............................S38

1.21. Depletion of GSH in solution..S39-S40

2. In vitro study………………………………………………………..............................S41-S43

2.1. Cell culture……………………………………………….............................. S41-S43

2.2. Photo-toxicity and dark-toxicity in vitro……………………….......................... S41-S43

2.3. Cellular colocalization imaging.. S41-S43

2.4. Flow cytometry analysis Apoptosis.. S41-S43

2.5. Detection of intracellular ROS generation……………………......................... S41-S43

2.6. Intracellular lipid peroxides measurement.. S41-S43

2.7. Analysis of mitochondrial membrane potential (MMP)... S41-S43

2.8. Cellular GSH detection.. S41-S43

2.9. Western blot analysis for GPX4 protein.. S41-S43
3. In vivo antitumor experiments...S44
4. Computational studies...S47-S52
5. Characterization of compounds..S53-S67

1. Experimental Results

1.1. General Information

All reactions were carried out under an aerobic or O_2 atmosphere as specified. All the chemicals were purchased from Aldrich, TCI, Alfa Aesar, or Spectrochem, and they were used without further purification. All glasswares were washed properly and dried in an oven before use. Commercial solvents were purified according to procedures described in Perrin's handbook. TLC (Thin Layer Chromatography) was performed on silica gel coated aluminum plates (MERCK, 60F254), which were visualized by UV fluorescence. Manual flash column chromatography was performed using high-purity grade silica gel, pore size 60 Å, 200-400 mesh particle size. Preparative Thin Layer Chromatography was performed on silica gel-coated glass plates (silica gel GF-254). ^{1}H and ^{13}C NMR spectroscopy were performed on a Bruker FT-NMR spectrometer (400 MHz and 500 MHz). The coupling constants (J) are reported in hertz (Hz) and corresponding multiplicity (s = singlet, d = doublet, t = triplet, m = multiplet). Reference for reported products is given after the characterization data of products. UV/vis absorption spectra were recorded on a Shimadzu UV-2450 UV/vis spectrophotometer, fluorescence emission spectra were recorded on a Shimadzu RF-6000 fluorescence spectrophotometer, and High-resolution mass spectrometry (HRMS) was recorded on a JEOL-AccuTOF JMS-T100L mass spectrometer.

1.2. Synthesis and Structural Characterization:

The synthetic route employed for the synthesis of two phenanthroline-BIP based ancillary ligands (**Ligand-1 and Ligand-2**) and photocatalysts **(Ir1, Ir2, Ir3, and Ir4)** is outlined in **Scheme S1** and **S2**.

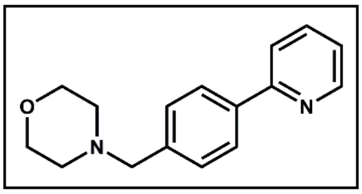

4-(4-(pyridin-2-yl)benzyl)morpholine:

2-(4-(bromomethyl)phenyl)pyridine was taken in CH_3CN solvent with morpholine (1.2 eq.) and K_2CO_3 (2 eq.). The resulting mixture was heated at 80 ºC for 16 h. Then the solution was evaporated to dryness, followed by aqueous workup with EtOAc to extract, and purified by column chromatography by 10% EtOAc/hexane. ^{1}H NMR (400 MHz, $CDCl_3$) δ 8.72–8.60 (m, 1H), 7.93 (d, J = 8.1 Hz, 2H), 7.76–7.65 (m, 2H), 7.42 (d, J = 8.0 Hz, 2H), 7.25–7.14 (m,

1H), 3.74–3.62 (s, 4H), 3.55 (s, 2H), 2.47 (s, 4H). ^{13}C NMR (126 MHz, $CDCl_3$) δ 157.33, 149.73, 138.54, 136.76, 129.68, 126.92, 122.08, 120.47, 66.99, 63.12, 53.66.

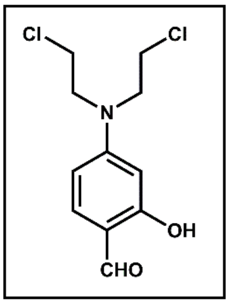

4-(Bis(2-chloroethyl)amino)-2-hydroxybenzaldehyde:

The following compound was synthesized according to the reported method.[1] Freshly distilled $POCl_3$ (1.8 mL, 19 mmol) was added dropwise to anhydrous DMF (3 mL) at 0 ºC. The mixture was stirred for 20 min at room temperature before 2,2'-((3-hydroxyphenyl)azanediyl)bis(ethan-1-ol) (1.08 g, 5.5 mmol) in DMF (3 mL) was added. The resulting mixture was heated at 80 ºC for 8 h. Then the solution was poured into ice and quenched with aq. NaOH and extracted with EtOAc, purified by column chromatography by 10% EtOAc/hexane. ^{1}H NMR (500 MHz, $CDCl_3$) δ 11.51 (s, 1H), 9.58 (s, 1H), 7.35 (d, J = 8.8 Hz, 1H), 6.30 (dd, J = 8.8, 1H), 6.12 (d, J = 2.3 Hz, 1H), 3.80 (t, J = 6.9 Hz, 4H), 3.67 (t, J = 6.9 Hz, 4H). ^{13}C NMR (126 MHz, $CDCl_3$) δ 193.08, 164.37, 153.30, 135.82, 113.00, 104.58, 98.20, 53.48, 40.17.

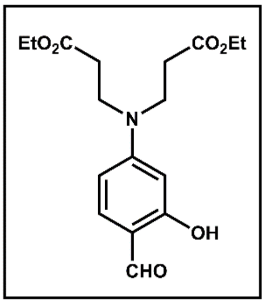

Diethyl 3,3'-((4-formyl-3-hydroxyphenyl)azanediyl)dipropionate:

The following compound was synthesized according to the reported method.[1] Freshly distilled $POCl_3$ (1.8 mL, 19 mmol) was added dropwise to anhydrous DMF (3 mL) at 0 ºC. The mixture was stirred for 20 min at room temperature before diethyl 3,3'-((3-hydroxyphenyl)azanediyl)dipropionate (1.69 g, 5.5 mmol) in DMF (3 mL) was added. The resulting mixture was heated at 60 ºC for 8 h. Then the solution was poured into ice and quenched with aq. NaOH and extracted with EtOAc, purified by column chromatography by 10% EtOAc/hexane. ^{1}H NMR (400 MHz, $CDCl_3$) δ 11.53 (s, 1H), 9.53 (s, 1H), 7.30 (d, J = 8.9 Hz, 1H), 6.28 (dd, J = 8.9, 2.4 Hz, 1H), 6.08 (d, J = 2.3 Hz, 1H), 4.24–4.05 (m, 4H), 3.84–3.65 (m, 4H), 2.73–2.46 (m, 4H), 1.24 (t, J = 7.1 Hz, 6H). ^{13}C NMR (126 MHz, $CDCl_3$) δ 192.69, 171.39, 164.37, 153.72, 135.60, 112.41, 104.74, 97.85, 60.97, 46.84, 32.50, 14.24.

Synthesis and Characterization of Ligand-1 and Ligand-2:

Ligands 1 and 2 were synthesised following the literature procedure.[2] A 1: 1 mixture of 5,6-phenanthrolinedione and the corresponding aldehydes in AcOH and an excess of NH_4OAc were heated to reflux for 2 to 4 h. A yellow solid for ligand 1 and a grey solid for ligand 2 precipitated upon cooling and neutralizing with excess ammonia solution, which was isolated by filtration, washed with water, and vacuum dried.

Ligand-1 (5-(bis(2-chloroethyl)amino)-2-(1*H*-imidazo[4,5-*f*][1,10]phenanthrolin-2-yl)phenol): yellow solid; ^{1}H NMR (400 MHz, DMSO-d_6) δ 13.65 (s, 1H), 12.73 (s, 1H), 9.05 (d, *J* = 3.1 Hz, 2H), 8.95 (d, *J* = 8.2 Hz, 1H), 8.88 (d, *J* = 6.9 Hz, 1H), 8.01 (d, *J* = 8.8 Hz, 1H), 7.86 (d, *J* = 10.8 Hz, 2H), 6.57 (d, J = 9.7 Hz, 1H), 6.38 (s, 1H), 3.82 (s, 8H). ESI-MS Calcd for $[C_{23}H_{19}Cl_2N_5O]^+$: 451.0967 m/z, Found: 451.0948 m/z.

Ligand-2 (diethyl 3,3'-((3-hydroxy-4-(1*H*-imidazo[4,5-*f*][1,10]phenanthrolin-2-yl)phenyl)azanediyl)dipropionate): brown solid; ^{1}H NMR (400 MHz, DMSO-d_6) δ 12.75 (s, 1H), 9.04 (d, *J* = 4.2 Hz, 2H), 8.89 (d, *J* = 7.6 Hz, 2H), 7.97 (d, *J* = 8.6 Hz, 1H), 7.84 (dd, *J* = 7.8, 4.3 Hz, 2H), 6.49 (d, *J* = 8.7 Hz, 1H), 6.31 (s, 1H), 4.09 (dd, *J* = 14.2, 7.1 Hz, 4H), 3.67 (t, *J* = 6.7 Hz, 4H), 2.62 (t, *J* = 6.7 Hz, 4H), 1.20 (t, *J* = 7.1 Hz, 6H). ESI-MS Calcd for $[C_{29}H_{29}N_5O_5]^+$: 527.2169 m/z, Found: 527.2152 m/z.

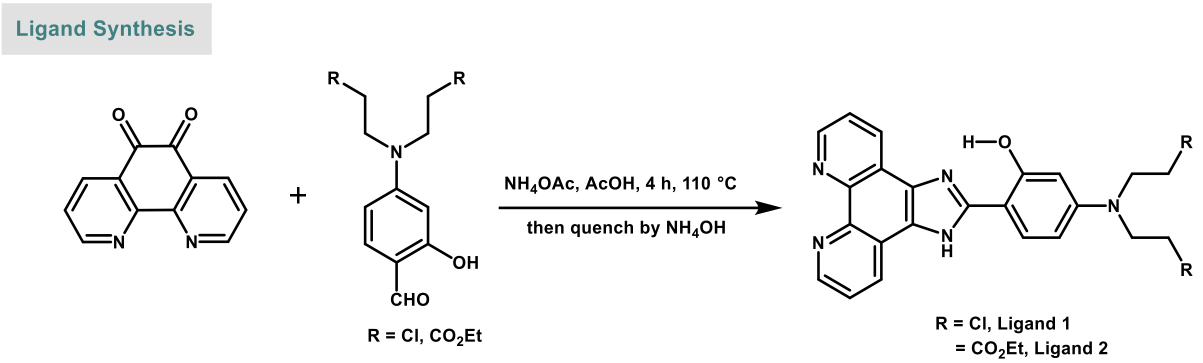

Scheme S1. Synthetic procedure of ligand 1 and ligand 2.

Synthesis of Ir (III) complexes (Ir1, Ir2, Ir3 and Ir4):

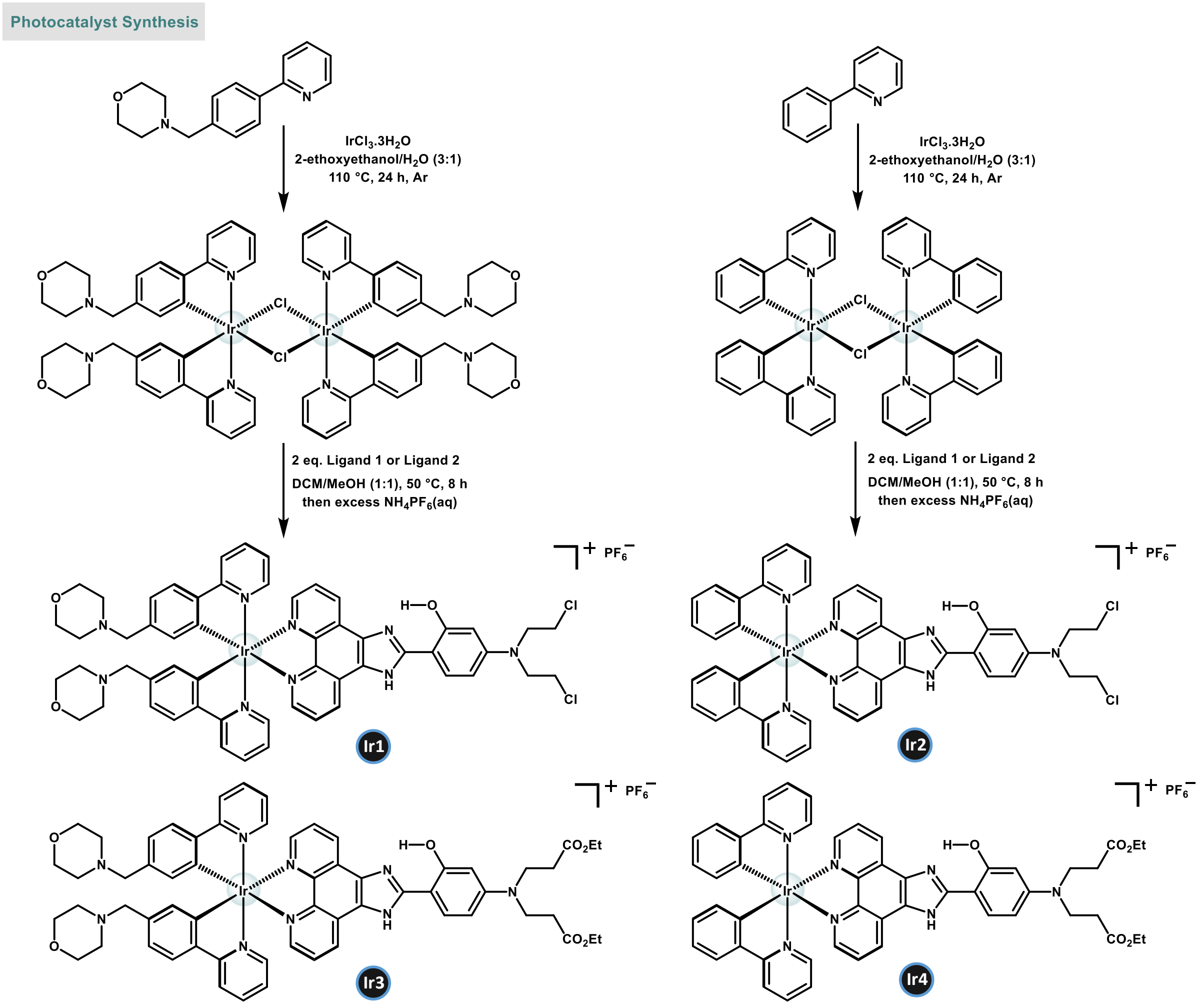

Scheme S2. Synthesis routes of Ir (III) complexes (Ir1, Ir2, Ir3 and Ir4).

A. Synthesis of $[Ir(ppy)_2Cl]_2$

$IrCl_3 \cdot 3H_2O$ (194.0 mg, 0.55 mmol) and 2-phenylpyridine (190.0 mg, 1.22 mmol) were dissolved in a mixture of 2-ethoxyethanol (30 mL) and water (10 mL), which was refluxed for 24 h under an argon atmosphere.[3] Then, the solution was cooled to room temperature, and the resulting yellow precipitate was collected through filtration. The precipitate was successively washed with ethanol (30 mL) and acetone (30 mL) to obtain a yellow solid with a yield of 72% (214.0 mg).

B. Synthesis of $[Ir(ppy\text{-}morpholine)_2Cl]_2$

$IrCl_3 \cdot 3H_2O$ (194.0 mg, 0.55 mmol) and 4-(4-(pyridin-2-yl)benzyl)morpholine (310.0 mg, 1.22 mmol) were dissolved in a mixture of 2-ethoxyethanol (30 mL) and water (10 mL), which was refluxed for 24 h under an argon atmosphere.[3] Then, the solution was cooled to room temperature, and the resulting yellow precipitate was collected through filtration. The precipitate was successively washed with ethanol (30 mL) and acetone (30 mL) to obtain an orange solid with a yield of 47% (186.1 mg).

Synthesis of four ESIPT Ir(III) complexes (Ir-1, Ir-2, Ir-3 and Ir-4):

A. Synthesis of Ir-1

$[Ir(ppy\text{-}morpholine)_2Cl]_2$ (50 mg, 0.034 mmol) and Ligand-1 (33.9 mg, 0.075 mmol) were dissolved in CH_2Cl_2/MeOH (12 mL, 2:1, v/v). Then, the mixture was refluxed at 50 ºC for 8 h under an argon atmosphere. After the reaction finished, the mixture was cooled to room temperature, and a 10-fold excess of ammonium hexafluorophosphate (NH_4PF_6) was added. The suspension solution was stirred for 30 min and then filtered to remove insoluble inorganic salts. The solution was evaporated to dryness under reduced pressure to obtain a yellow solid. The crude product was further purified with column chromatography (silica gel, dichloromethane/methanol = 10:1, v/v) to obtain the yellow solid with a yield of 44.1 % (38.2 mg).

^{1}H NMR (400 MHz, DMSO-d_6) δ 14.86 (s, 1H), 12.38 (s, 1H), 11.23 (s, 1H), 9.67 (s, 1H), 9.18 (s, 1H), 8.35 (d, J = 7.8 Hz, 2H), 8.27 (s, 3H), 8.05 (d, J = 7.6 Hz, 4H), 7.93 (d, J = 7.8 Hz, 2H), 7.53 (s, 2H), 7.35 (m, 2H), 7.05 (d, J = 12.6 Hz, 2H), 6.35 (s, 3H), 4.06 (s, 4H), 3.89 (s, 4H), 3.80 (s, 12H), 2.87 (d, J = 12.8 Hz, 8H). ^{13}C NMR (126 MHz, DMSO-d_6) δ 165.83, 159.12, 159.12, 153.49, 153.49, 150.21, 149.80, 149.51, 145.34, 143.81, 138.89, 138.89, 134.47, 130.18, 128.29, 125.45, 124.77, 124.77, 120.28, 120.28, 104.37, 102.13, 98.82, 62.92, 59.13, 51.79, 50.50, 41.11. ESI-MS Calcd for $[C_{55}H_{53}Cl_2IrN_9O_3]^+$: 1150.3272 m/z, Found: 1150.3232 m/z.

B. Synthesis of Ir-2

$[Ir(ppy)_2Cl]_2$ (50 mg, 0.047 mmol) and Ligand-1 (46.5 mg, 0.103 mmol) were dissolved in CH_2Cl_2/MeOH (15 mL, 2:1, v/v). Then, the mixture was refluxed at 50 ºC for 8 h under an argon atmosphere. After the reaction finished, the mixture was cooled to room temperature, and a 10-fold excess of ammonium hexafluorophosphate (NH_4PF_6) was added. The suspension solution was stirred for 30 min and then filtered to remove insoluble inorganic salts. The solution was evaporated to dryness under reduced pressure to obtain an orange solid. The crude product was further purified with column chromatography (silica gel, dichloromethane/methanol = 10:1, v/v) to obtain the orange solid with a yield of 63.0 % (72 mg).

^{1}H NMR (500 MHz, DMSO-*d*$_6$) δ 14.38 (s, 1H), 12.26 (s, 1H), 8.26 (d, *J* = 8.1 Hz, 2H), 8.15 (d, *J* = 4.4 Hz, 2H), 8.05 (d, *J* = 6.2 Hz, 2H), 7.96 (d, J = 7.8 Hz, 2H), 7.88 (t, *J* = 7.7 Hz, 2H), 7.53 (d, *J* = 5.6 Hz, 2H), 7.22 (s, 2H), 7.12 (s, 2H), 7.07 (t, *J* = 7.5 Hz, 2H), 7.02 (d, *J* = 4.3 Hz, 2H), 6.96 (m, 3H), 6.30 (d, *J* = 7.5 Hz, 2H), 3.81 (s, 8H). ^{13}C NMR (126 MHz, DMSO-*d*$_6$) δ 166.84, 159.02, 153.32, 150.23, 149.88, 149.09, 148.62, 143.96, 140.84, 138.58, 132.41, 131.15, 130.16, 128.03, 126.80, 124.96, 123.72, 122.28, 119.85, 104.38, 102.13, 98.91, 51.80, 41.10. ESI-MS Calcd for $[C_{45}H_{35}Cl_2IrN_7O]^+$: 952.1904 m/z, Found: 952.1883 m/z.

C. Synthesis of Ir-3

$[Ir(ppy\text{-}morpholine)_2Cl]_2$ (50 mg, 0.034 mmol) and Ligand-2 (39.5 mg, 0.075 mmol) were dissolved in CH_2Cl_2/MeOH (12 mL, 2:1, v/v). Then, the mixture was refluxed at 50 ºC for 8 h under an argon atmosphere. After the reaction finished, the mixture was cooled to room temperature, and a 10-fold excess of ammonium hexafluorophosphate (NH_4PF_6) was added. The suspension solution was stirred for 30 min and then filtered to remove insoluble inorganic salts. The solution was evaporated to dryness under reduced pressure to obtain a reddish-brown solid. The crude product was further purified with column chromatography (silica gel, dichloromethane/methanol = 10:1, v/v) to obtain the reddish brown solid with a yield of 46.7 % (48 mg).

^{1}H NMR (400 MHz, $CDCl_3$) δ 9.03 (s, 2H), 8.17 (s, 2H), 7.90 (m, 3H), 7.72 (m, 4H), 7.66 (d, *J* = 7.9 Hz, 2H), 7.37 (s, 2H), 7.04 (d, *J* = 7.7 Hz, 2H), 6.88 (s, 2H), 6.31 (s, 2H), 6.24 (s, 1H), 6.11 (s, 1H), 4.16 (q, 4H), 3.60 (m, 12H), 3.38 (q, 4H), 2.59 (m, 4H), 2.33 (m, 8H), 1.27 (t,

6H). ^{13}C NMR (126 MHz, $CDCl_3$) δ 171.96, 168.40, 168.00, 160.03, 150.34, 148.82, 148.54, 142.75, 138.17, 132.36, 127.79, 124.75, 123.65, 123.16, 119.58, 108.49, 105.06, 102.30, 101.55, 100.16, 99.15, 66.99, 63.08, 60.93, 53.51, 46.75, 32.77, 14.34. ESI-MS Calcd for $[C_{61}H_{63}IrN_9O_7]^+$: 1226.4474 m/z, Found: 1226.4455 m/z.

D. Synthesis of Ir-4

$[Ir(ppy)_2Cl]_2$ (50 mg, 0.047 mmol) and Ligand-2 (54.3 mg, 0.103 mmol) were dissolved in CH_2Cl_2/MeOH (15 mL, 2:1, v/v). Then, the mixture was refluxed at 50 ºC for 8 h under an argon atmosphere. After the reaction finished, the mixture was cooled to room temperature, and a 10-fold excess of ammonium hexafluorophosphate (NH_4PF_6) was added. The suspension solution was stirred for 30 min and then filtered to remove insoluble inorganic salts. The solution was evaporated to dryness under reduced pressure to obtain a yellowish-green solid. The crude product was further purified with column chromatography (silica gel, dichloromethane/methanol = 10:1, v/v) to obtain the yellowish-green solid with a yield of 64.5 % (78 mg).

^{1}H NMR (500 MHz, $CDCl_3$) δ 9.27 (s, 2H), 8.19 (d, J = 4.5 Hz, 2H), 8.11 (s, 1H), 7.93 (d, J = 8.1 Hz, 2H), 7.72 (m, 6H), 7.39 (d, J = 4.8 Hz, 2H), 7.09 (t, J = 7.4 Hz, 2H), 6.98 (t, J = 7.2 Hz, 2H), 6.89 (s, 2H), 6.41 (d, J = 7.4 Hz, 2H), 6.24 (s, 2H), 4.16 (q, J = 7.2 Hz, 4H), 3.66 (m, 4H), 2.60 (t, J = 6.6 Hz, 4H), 1.27 (t, J = 7.2 Hz, 6H). ^{13}C NMR (126 MHz, DMSO-d_6) δ 171.44, 166.87, 159.02, 153.49, 150.28, 150.07, 149.12, 148.26, 143.97, 143.76, 138.62, 132.19, 131.17, 130.20, 127.64, 126.82, 125.00, 123.76, 122.32, 119.90, 104.41, 101.40, 98.68, 60.06, 46.00, 32.01, 14.01. ESI-MS Calcd for $[C_{51}H_{45}IrN_7O_5]^+$: 1028.3111 m/z, Found: 1028.3127 m/z.

1.3. UV-Visible Spectroscopy

Molar extinction coefficients for photocatalysts Ir1, Ir2, Ir3, and Ir4 were determined in the aprotic solvents like DCM and acetonitrile and in the protic solvents like MeOH and phosphate buffer saline (PBS) solution. Stock solutions were prepared with ~1.00 mg of each complex (Ir1, Ir2, Ir3, or Ir4) dissolved in 1.00 mL DCM to make (1×10^{-3} M) solution. Three stock solutions of each photocatalyst were prepared. Then, 40 µL aliquots of each stock solution were diluted to 4.00 mL with respective solvents, and the electronic absorption of each diluted sample was measured with a Shimadzu UV-2450 spectrophotometer.

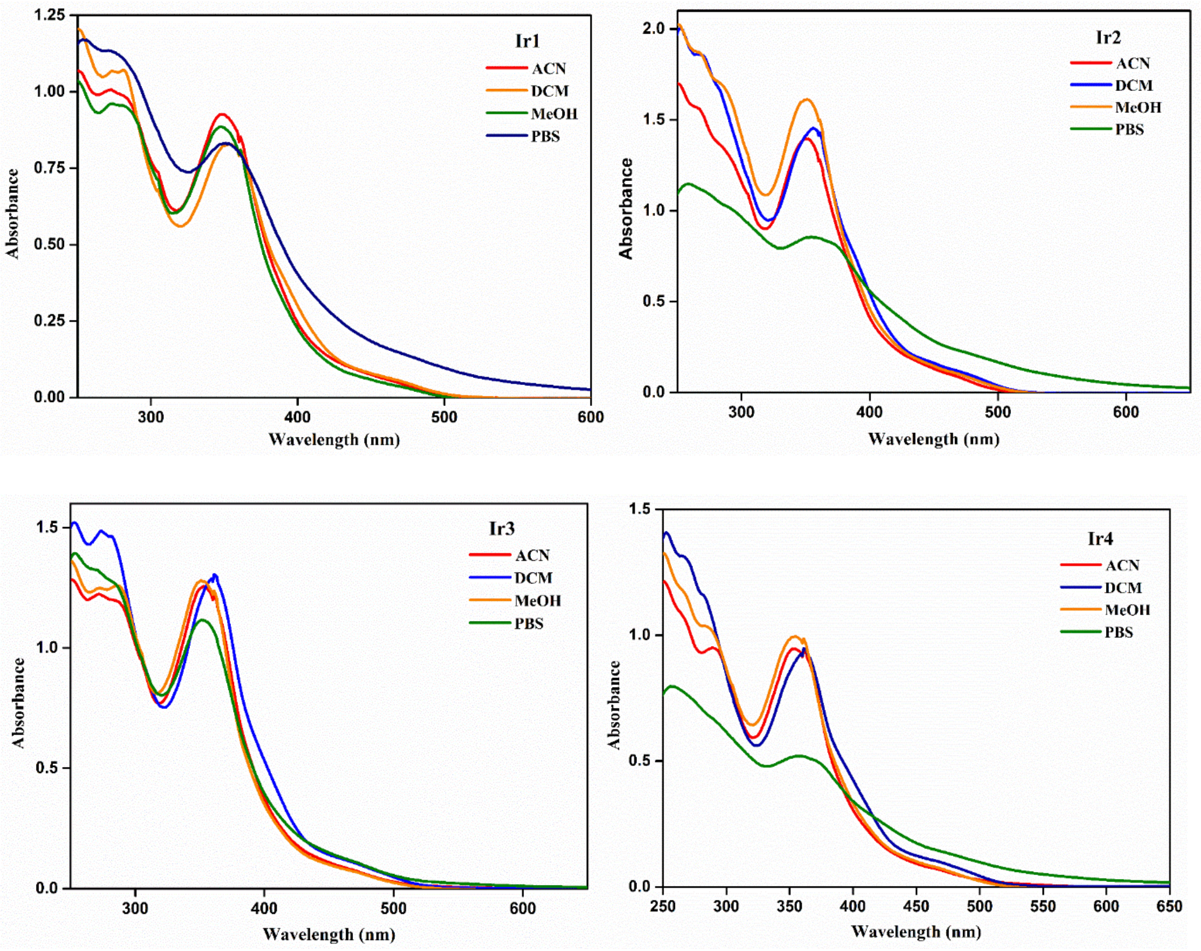

Figure S1. Molar extinction coefficients of photocatalysts Ir1, Ir2, Ir3 and Ir4 in acetonitrile, DCM, MeOH and phosphate buffer saline (PBS) solution.

1.4. Stability Tests

The photostability of all Ir-complexes (10 μM) was investigated by recording the absorption spectra in DMSO and PBS (1% DMSO, v%) in dark or illumination with 405 nm blue LED light for various time intervals.

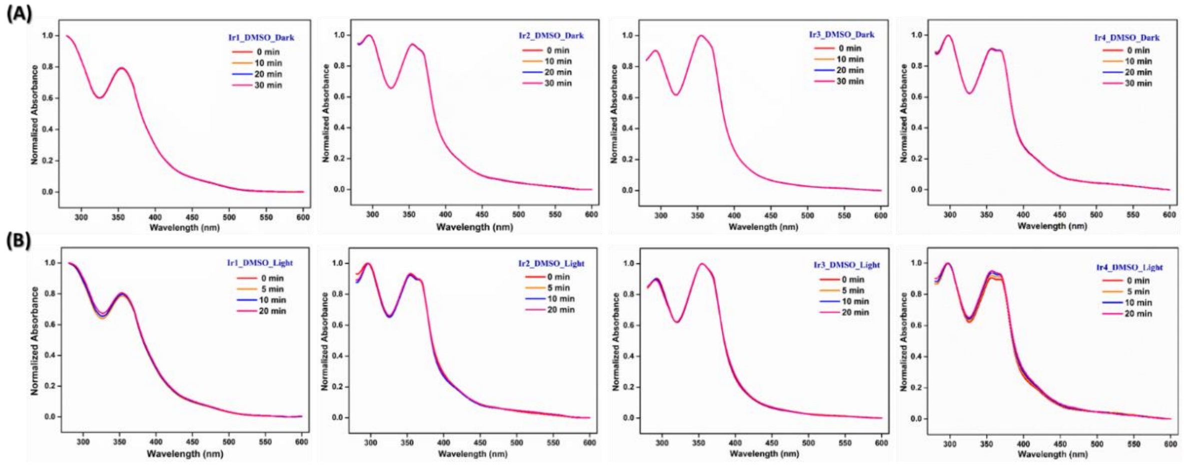

Figure S2. The photostability tests of Ir1-Ir4. (A) Absorption of Ir1-Ir4 (10 μM) in DMSO for 0–30 min. (B) Absorption of Ir1-Ir4 (10 μM) in DMSO after irradiation by 405 nm blue LED for 0–20 min.

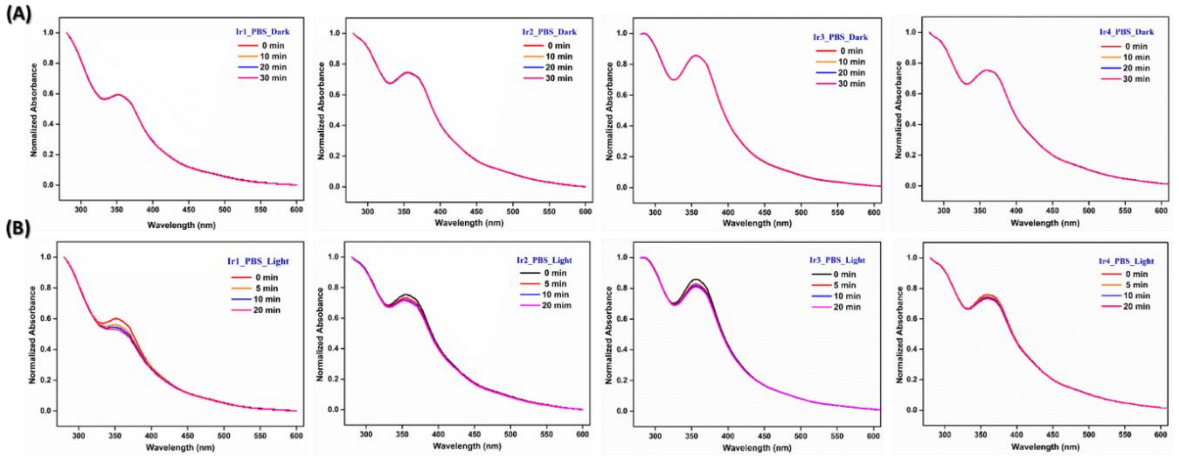

Figure S3. The photostability tests of Ir1-Ir4. (A) Absorption of Ir1-Ir4 (10 μM) in PBS (1% DMSO, v%) for 0–30 min. (B) Absorption of Ir1-Ir4 (10 μM) in PBS (1% DMSO, v%) after irradiation by 405 nm blue LED for 0–20 min.

1.5. Steady-State Emission Spectroscopy

Samples for emission spectroscopy were similarly prepared as described for absorption spectra. Fluorescence emission spectra for photocatalysts Ir1, Ir2, Ir3, and Ir4 were also determined in the aprotic solvents like DMSO, DCM, and acetonitrile, and in the protic solvents like MeOH and phosphate buffer saline (PBS) solution using Shimadzu RF-6000 spectrofluorometer. The corresponding emission maxima are listed in the experimental section of the corresponding compound. The fluorescence emission spectra of Ir1, Ir2, Ir3, and Ir4 are shown below (**Fig. S4**). The change in emission intensity of Ir1-Ir-4 complexes and Ligands in DCM with adding 1 mM TFA is recorded (**Fig. S5**).

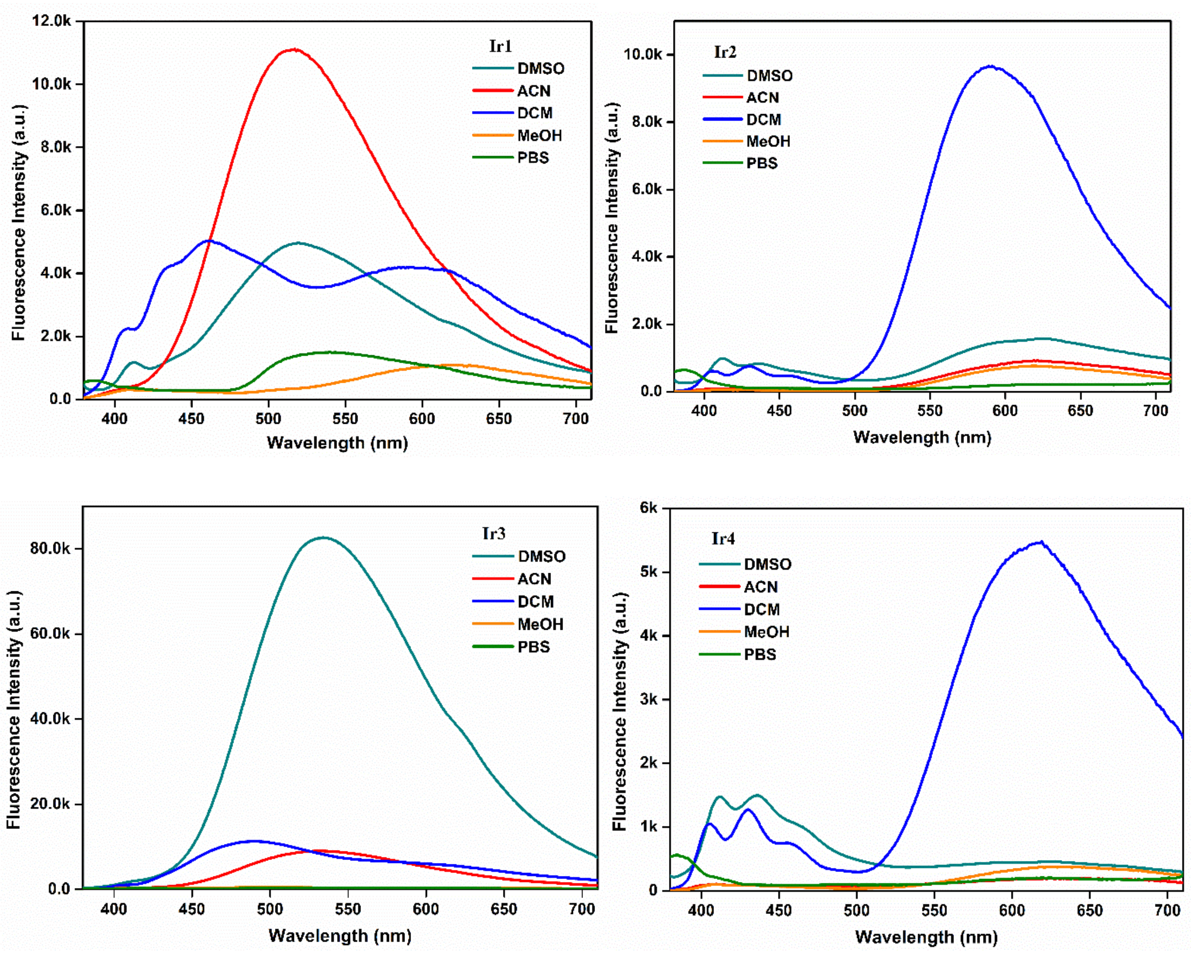

Figure S4. Steady-state emission spectra of photocatalysts Ir1, Ir2, Ir3, and Ir4 in DMSO, acetonitrile, DCM, MeOH, and phosphate buffer saline (PBS) solution. Samples were excited at 365 nm wavelength.

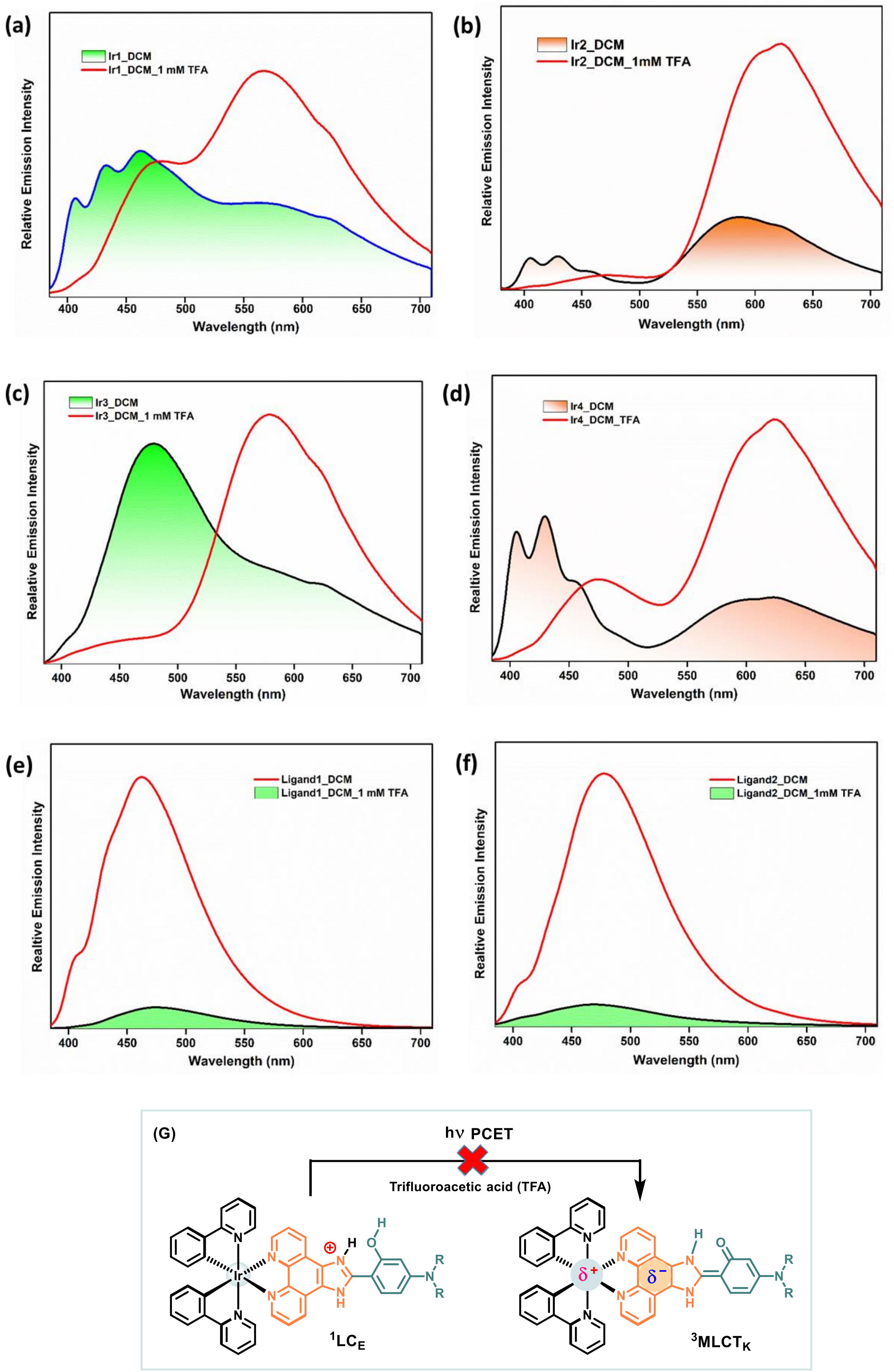

Figure S5. (a)-(d) Emission quantum yield of Ir1-Ir4 increases in DCM with the addition of 1 mM TFA. (e)-(f) Emission quantum yield of Ligand1 and 2 decreases in DCM with the addition

of 1 mM TFA. Samples were excited at 365 nm wavelength. (G) Quenching of intramolecular PCET in presence of TFA for Ir-complexes.

1.6. Fluorescence Lifetimes (τ_F)

The TCSPC measurement, which is actually a time-resolved emission measurement, gives us the excited state decay dynamics of a fluorescent molecule through the excited state lifetime information. In the TCSPC measurement module, we have used a picosecond diode LASER unit with its peak wavelength at 375 nm excitation LASER source. The fluorescence signals generated are detected using the Hamamatsu MCP PMT (3809U) detector at the polarizer angle of ~54.7°, which is called magic angle polarization. The TCSPC decays are collected in forward mode and analyzed with respect to the prompt (or instrument response function (IRF)) that has been taken. The IBH DAS-6 software has been utilized to fit the collected decays using the respective order of exponential equation to get the subsequent lifetime values and contributions. The average lifetime (τ_{avg}) has been calculated using the obtained lifetime information following the equation below, where τ_i is the lifetime component, and a_i is the respective amplitude. Contributions with i represent the order of the exponential equation used to fit the lifetime decay trace, and $\Sigma\, a_i = 1$.

$$\tau_{ave} = \sum a_i \tau_i \text{ ....(1)}$$

The monitored emission wavelengths and the corresponding fluorescence lifetimes (τ_F) are indicated in the Fig. S6 and Table S1.

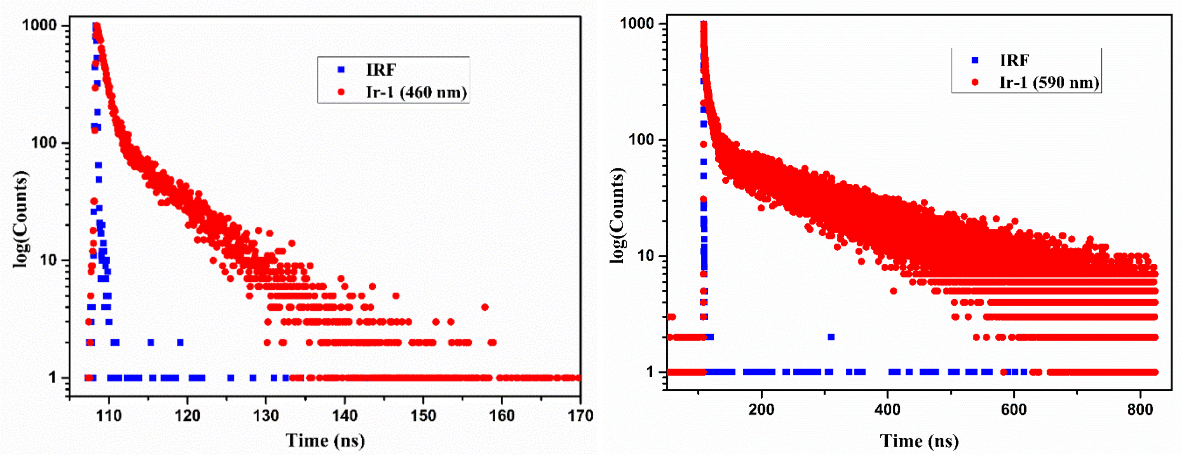

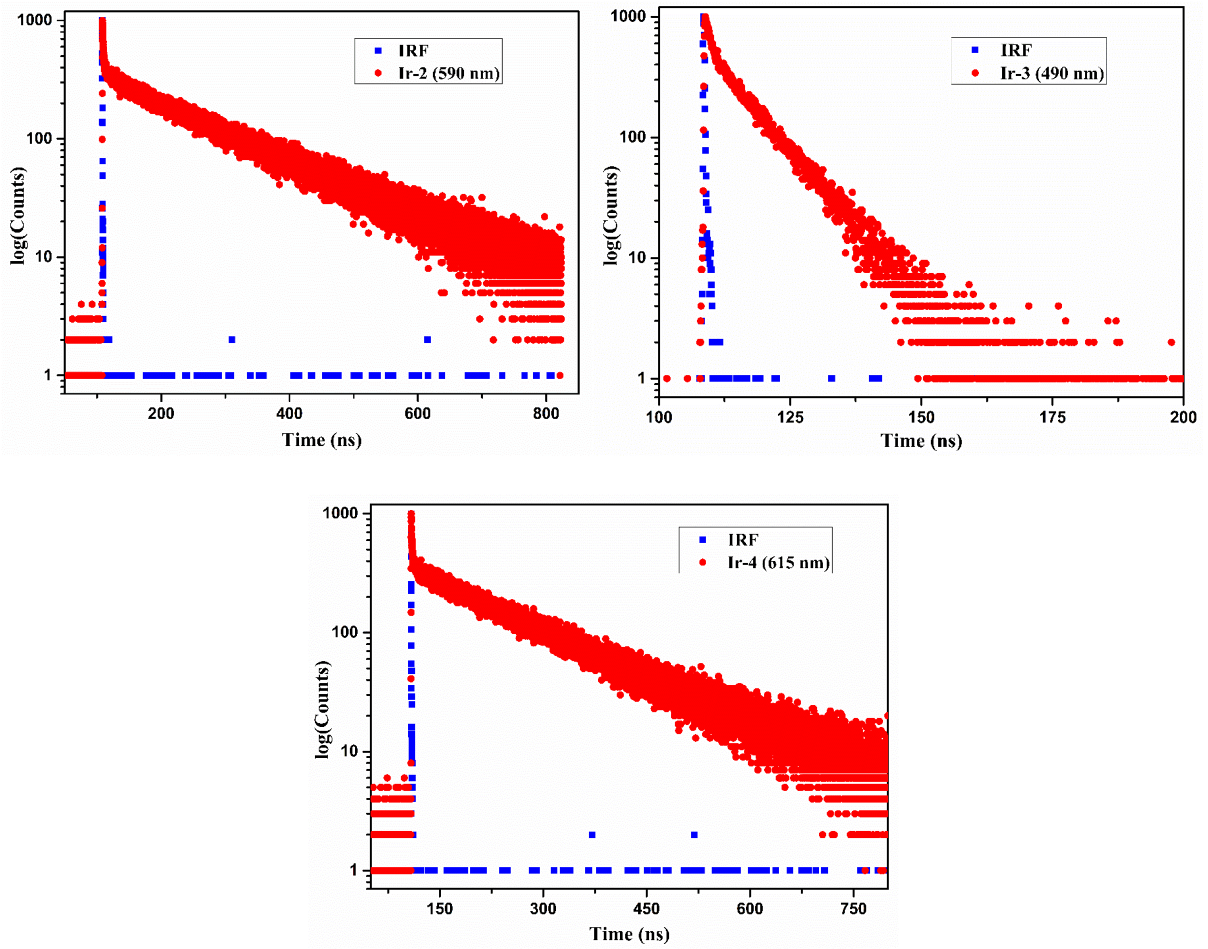

Figure S6. TCSPC decay profile at the excitation wavelength of 370 nm, red circles denote the decay traces of Ir-complexes (Ir1, Ir2, Ir3, Ir4) at the corresponding emission maxima in the 20 μM DCM solution, and blue squares indicate the IRF.

Table S1. The fluorescence lifetimes (τ_F) of corresponding Ir complexes in DCM.

Complex	@Wavelength (nm)	TCSPC lifetime (τ_F)
Ir-1	450	1.476 ± 0.5634 ns
	590	25.6128 ± 5.0451 ns
Ir-2	590	62.1164 ± 1.39245 ns
Ir-3	490	4.01394 ± 0.2534 ns
Ir-4	615	52.6876 ± 1.07024 ns

1.7. Fluorescence Quantum Yields

The fluorescence quantum yield (Φ_F) is defined as the fraction of the number of quanta absorbed by a molecule that is emitted as fluorescence. The Φ_F of Ir1, Ir2, Ir3, and Ir4 were determined by the reference point method.[4] A solution of quinine sulfate (Φ_F = 0.546 in 0.5 M H_2SO_4) was used as the standard for the fluorescence quantum yield determinations following the equation given below. An error of 5% is estimated for the fluorescence quantum yields. The absorbance values of the solutions at the excitation wavelength were measured with a UV-Vis spectrophotometer. Photoluminescence (PL) emission spectra of all the sample solutions were recorded by Shimadzu RF-6000 spectrofluorophotometer at 365 nm excitation wavelength of compounds. Where Φ_F represents the fluorescence quantum yield, "A" is the absorbance monitored at the excitation wavelength, "F" designates the areas under the fluorescence emission curves, and "η" is the refractive index of the medium. For DMSO solvent, the refractive index is 1.4793, and for 0.5 M H_2SO_4, the refractive index is 1.346. The subscripts S and R denote the corresponding parameters for the sample and reference, respectively.

$$\Phi_{F(S)} = \Phi_{F(R)} \times \left(\frac{F_S}{F_R}\right) \times \left(\frac{A_R}{A_S}\right) \times \left(\frac{\eta_S}{\eta_R}\right)^2 \ldots\ldots\ldots (2)$$

Table S2. The fluorescence quantum yields (Φ_F) of Ir1, Ir2, Ir3, and Ir4 upon irradiation with light of 365 nm.

Ir(III)-Complex	Φ_F (in DMSO)
**Ir-1**	0.01
**Ir-2**	0.01
**Ir-3**	0.031
**Ir-4**	0.011

1.8. Phosphorescence Spectra and Lifetimes at 77 K

The phosphorescence emission measurements were performed using a HORIBA Jobin Yvon-spex Fluorolog-3 spectrofluorimeter at 77 K in THF glassy matrix. Each Ir-complexes (10^{-4} M) in THF was diluted from a stock solution in DCM (1 mM) to record phosphorescence emission and time-resolved spectra at 77 K in a liquid N_2 atmosphere with an excitation wavelength λ_{ex} = 365 nm. The 1 mL of each solution is taken in an NMR tube, and the solution is purged with Ar gas prior to recording phosphorescence. The sealed NMR tube containing the solution was slowly immersed in the liquid-nitrogen-filled Dewar flask. The frozen sample is excited with a pulsed xenon lamp for phosphorescence measurements. The fwhm lamp-pulse width is 3 μs. The emitted phosphorescence is measured using a photon-counting detector (PMT). The instrument was set in the phosphorescence lifetime mode, and the time range was set at 10 ms. Phosphorescence lifetime measurements used the same equipment. The raw data were analyzed with OriginPro 2016 software by using the exponential function to obtain the phosphorescence lifetime.

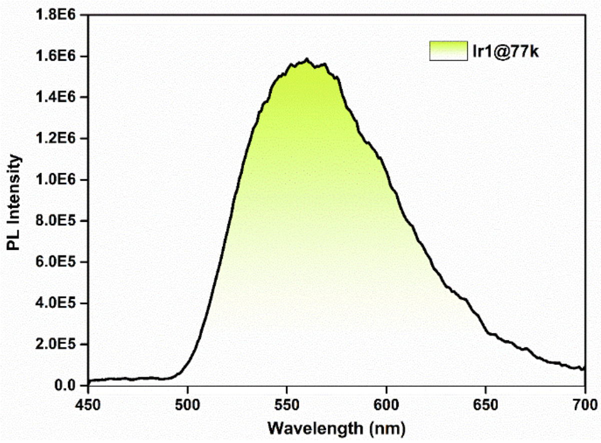

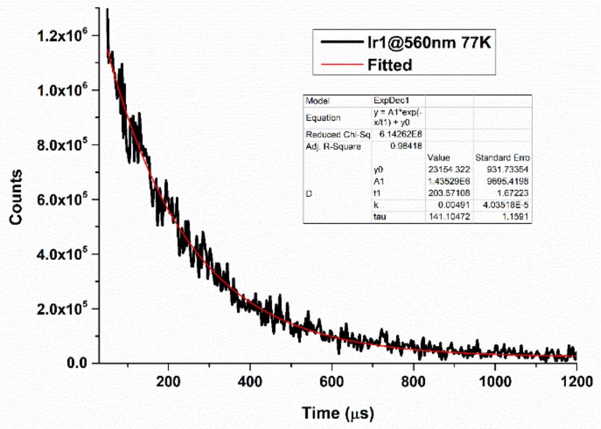

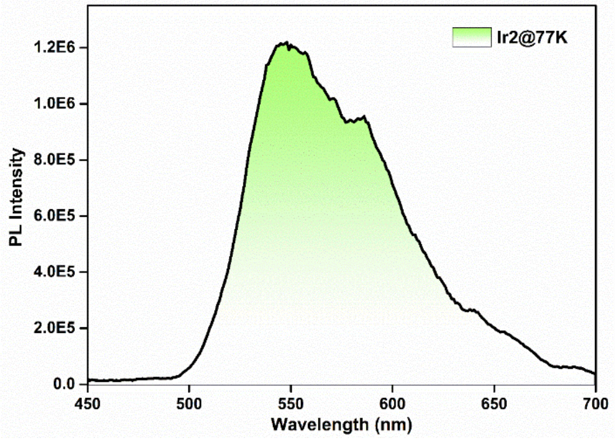

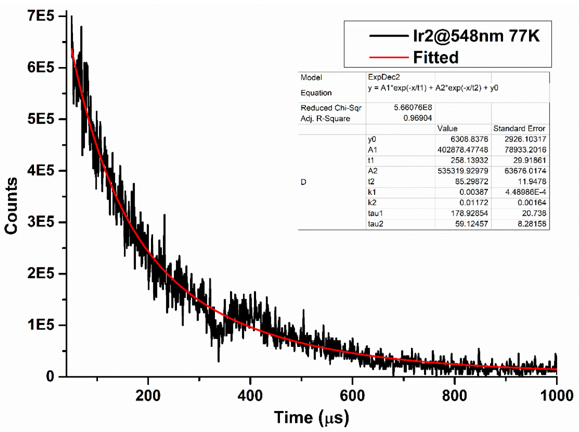

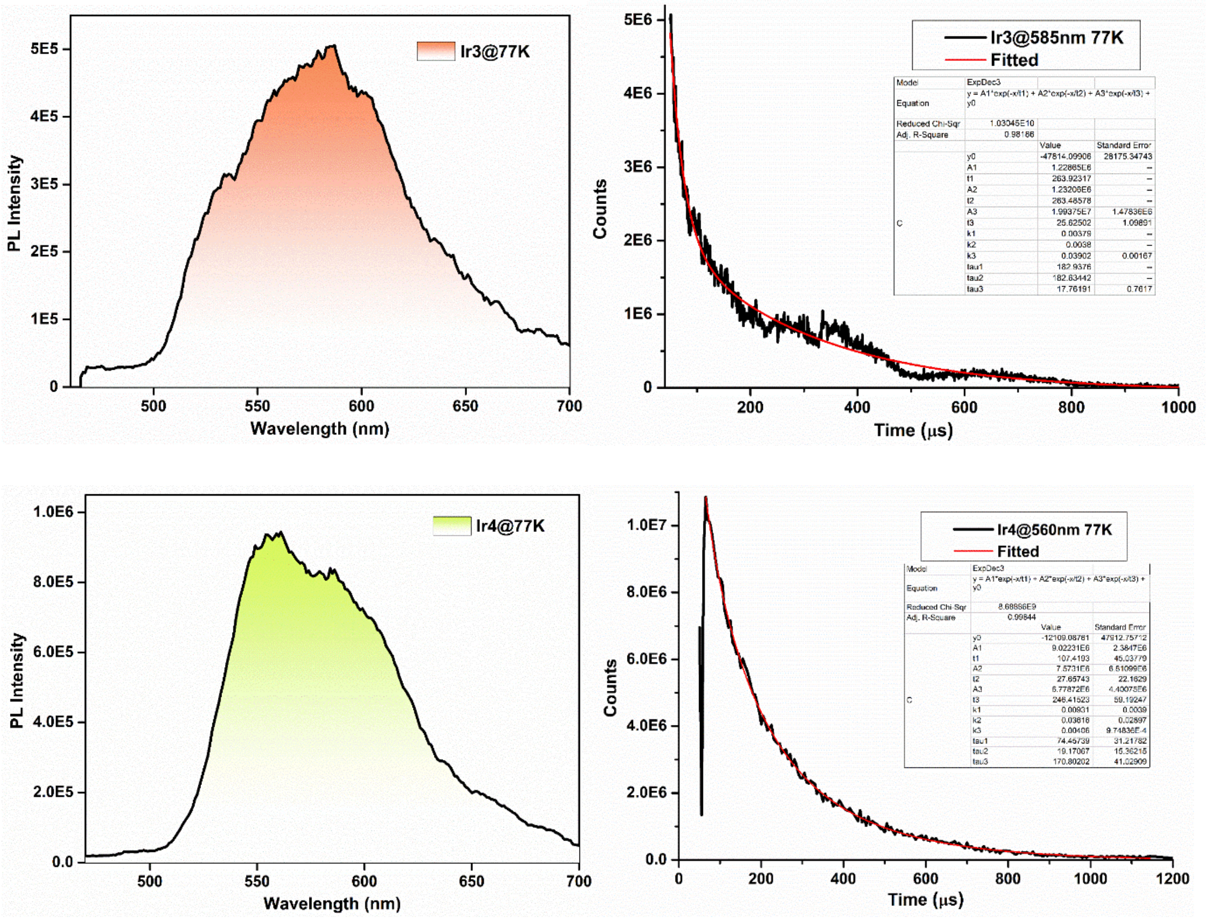

Figure S7. (a) Photoluminescence spectra of corresponding Ir-complexes (Ir1, Ir2, Ir3, Ir4) in deaerated THF glassy matrix at 77 K and emission decay curves at λ_{exc} = 370 nm with their corresponding $\lambda_{emi}^{(max)}$ wavelengths respectively, with luminescence lifetimes with relative emission amplitudes, from exponential fits.

Table S3. The phosphorescence lifetimes (τ_P) of corresponding Ir-complexes at 77 K in THF glassy matrix, λ_{irr} = 370 nm.

Complex	@Wavelength (nm)	Phosphorescence lifetimes@77K (τ_P) (μs)
Ir-1	560	203.57 ± 1.67
Ir-2	548	τ_1 = 258.14 ± 29.90; τ_2 = 85.30 ± 11.94
Ir-3	585	τ_1 = 263.92; τ_2 = 263.48; τ_3 = 25.63 ± 1.09
Ir-4	560	τ_1 = 107.42 ± 45.03; τ_2 = 27.65 ± 22.16; τ_3 = 246.42 ± 59.19

1.9. Triplet State Lifetimes (τ_T)

Phosphorescence lifetime (λ_{exc} = 370 nm) was measured on an FLS1000 spectrometer, Edinburgh Instruments equipped with a micro flash-lamp (µF2) set-up.

$$\text{Fit: } A+B1\exp(-t/\tau_1)+B2\exp(-t/\tau_2) \quad \ldots\ldots..(3)$$

$$\text{Fit: } A+B1\exp(-t/\tau_1)+B2\exp(-t/\tau_2)+B3\exp(-t/\tau_3) \ldots\ldots..(4)$$

Table S4. Triplet state lifetimes (τ_T) for all Ir(III) complexes in DCM, DCM (with 10 µM TFA), ACN, PBS buffer solution were recorded.

Complex	@Solvent	Phosphorescence lifetime (τ)
Ir-1	DCM	18.93 (11.80) + 241.3 (42.78) + 1073 (45.43) ns
	DCM (TFA)	21.44 (13.48) + 201.5 (50.38) + 632.4 (36.14) ns
	ACN	14.31 (27.53) + 89.86 (52.15) + 419.3 (20.33) ns
	PBS	23.74 (15.00) + 118.4 (73.30) + 560 (11.71) ns
Ir-2	DCM	261.2 (61.74) + 556.3 (38.26) ns
	DCM (TFA)	290.3 (65.38) + 550.5 (34.62) ns
	ACN	43.43 (19.42) + 133.9 (69.14) + 643.8 (11.44) ns
	PBS	43.45 (20.44) + 118.6 (68.04) + 652.5 (11.53) ns
Ir-3	DCM	4.761 (55.70) + 148.5 (12.00) + 458.1 (32.31) ns
	DCM (TFA)	5.639 (42.43) + 90.17 (27.51) + 432.5 (30.06) ns
	ACN	7.912 (85.17) + 68.82 (9.55) + 633.6 (5.27) ns
	PBS	3.071 (93.14) + 37.81 (3.82) + 329.1 (3.05) ns
Ir-4	DCM	21.18 (2.76) + 208.3 (68.82) + 498.2 (28.42) ns
	DCM (TFA)	253.9 (81.52) + 608.5 (18.48) ns
	ACN	19.21 (48.30) + 110.8 (40.58) + 536.1 (11.13) ns
	PBS	29.26 (65.66) + 100.4 (25.83) + 646.9 (8.51) ns

1.10. Electrochemical Measurements

All electrochemical experiments were performed using CH Instruments (model CHI700E electrochemical analyzer). Cyclic Voltammetry (CV) of all compounds was recorded inside the glove box under an inert N_2 atmosphere. The glassy carbon electrode was taken as the working electrode, standard double-junction Ag/AgCl (saturated KCl) as the reference electrode, and a Pt was taken as the counter electrode. 4 mL of 0.5 mM compound solution in anhydrous CH_3CN was taken in the presence of 0.1 M Tetrabutylammonium hexafluorophosphate (TBAP) as the supporting electrolyte, and the scan rate was 100, 200, 500 mV/s.

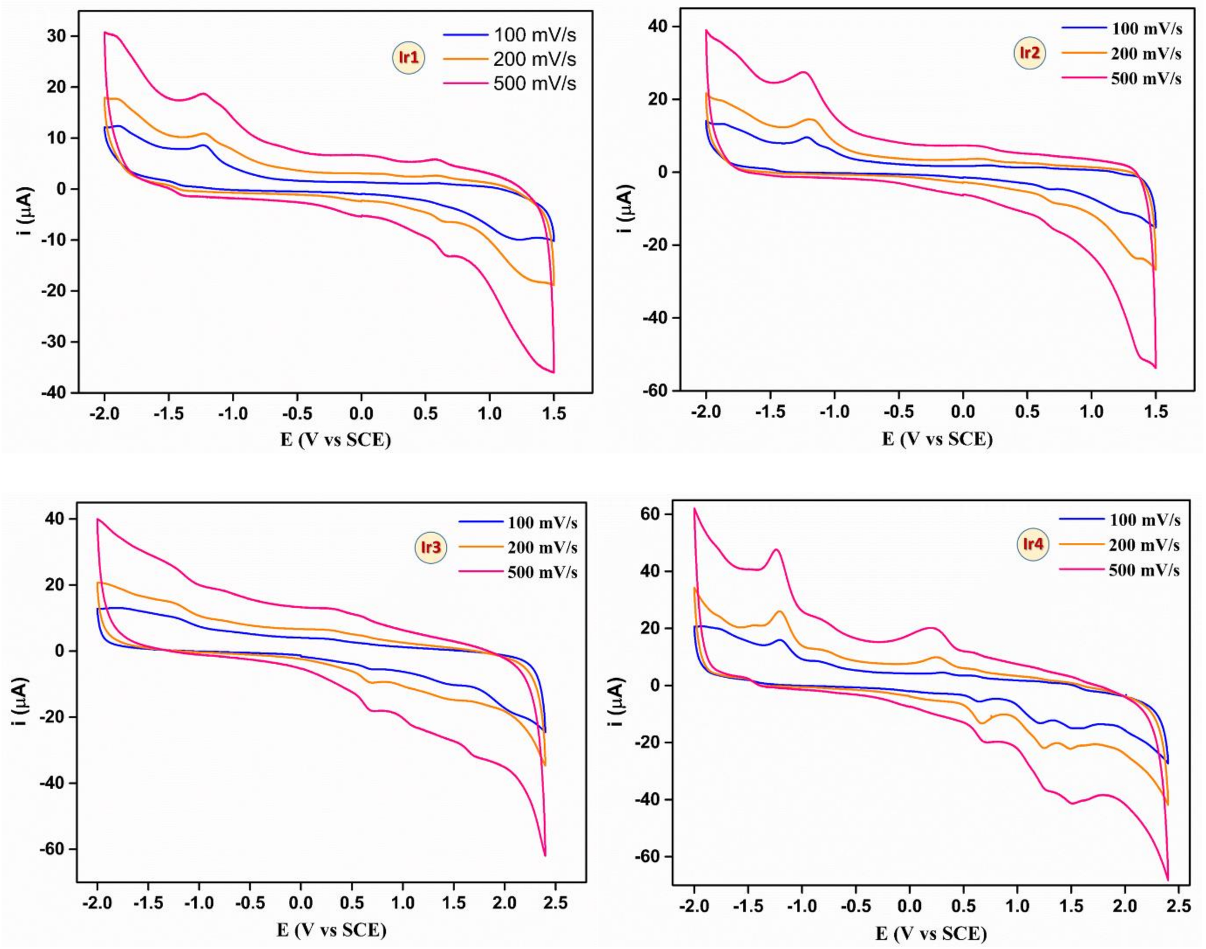

Figure S8. Cyclic voltammograms of Ir1-Ir4 under N_2 saturation, recorded at 100, 200, and 500 mV/s in ACN (catalyst concentration 0.5 mM) with TBAP as supporting electrolyte and glassy carbon working electrode.

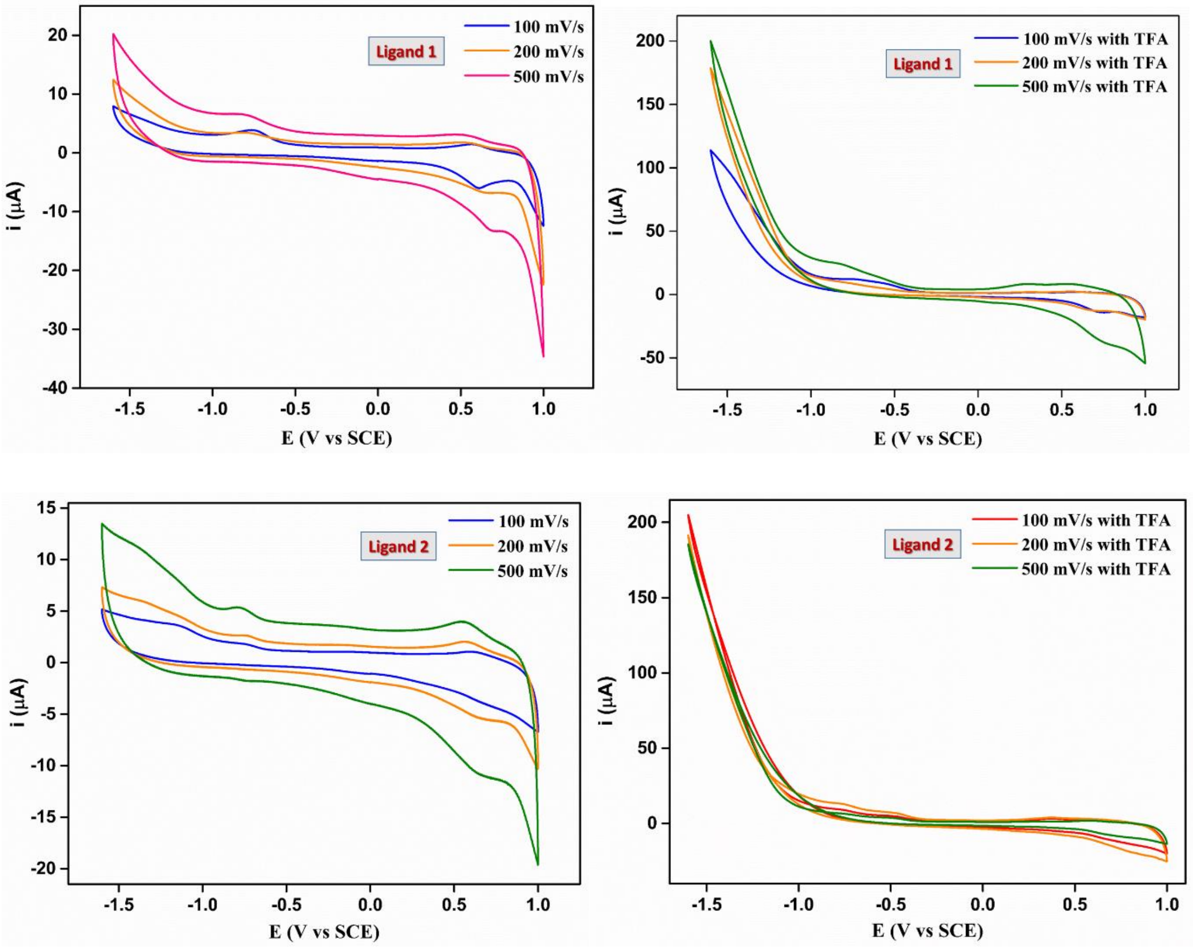

Figure S9. Cyclic voltammograms of uncoordinated Ligand 1 and 2 under N_2 saturation, recorded in the absence and presence of TFA at 100, 200, and 500 mV/s in ACN (concentration 0.5 mM) with TBAP as supporting electrolyte and glassy carbon working electrode.

Table S5. Electrochemical Potentials of Ir1−Ir4 photocatalysts and uncoordinated free ligands Phenanthrolin-BIP in acetonitrile.

Compound	$E_{1/2}[Ir^{IV}/Ir^{III}]^{b}$	$E_{1/2}[BIP^{\bullet+}/BIP]^{a}$	$E_{1/2}[Ir^{III}/Ir^{II}]$
**Ir-1**	1.23 V	0.65 V	−1.21 V
**Ir-2**	1.24 V	0.68 V	−1.22 V
**Ir-3**	1.48 V	0.69 V	−1.22 V
**Ir-4**	1.22 V	0.67 V	−1.19 V
**Ligand-1**	n/a	0.63 V	
**Ligand-2**	n/a	0.61 V	

[a]First peak potential for ligand; [b]Second peak potential for $Ir^{IV/III}$ couple.

1.11. Solvent-Modulated ESIPT Dynamics

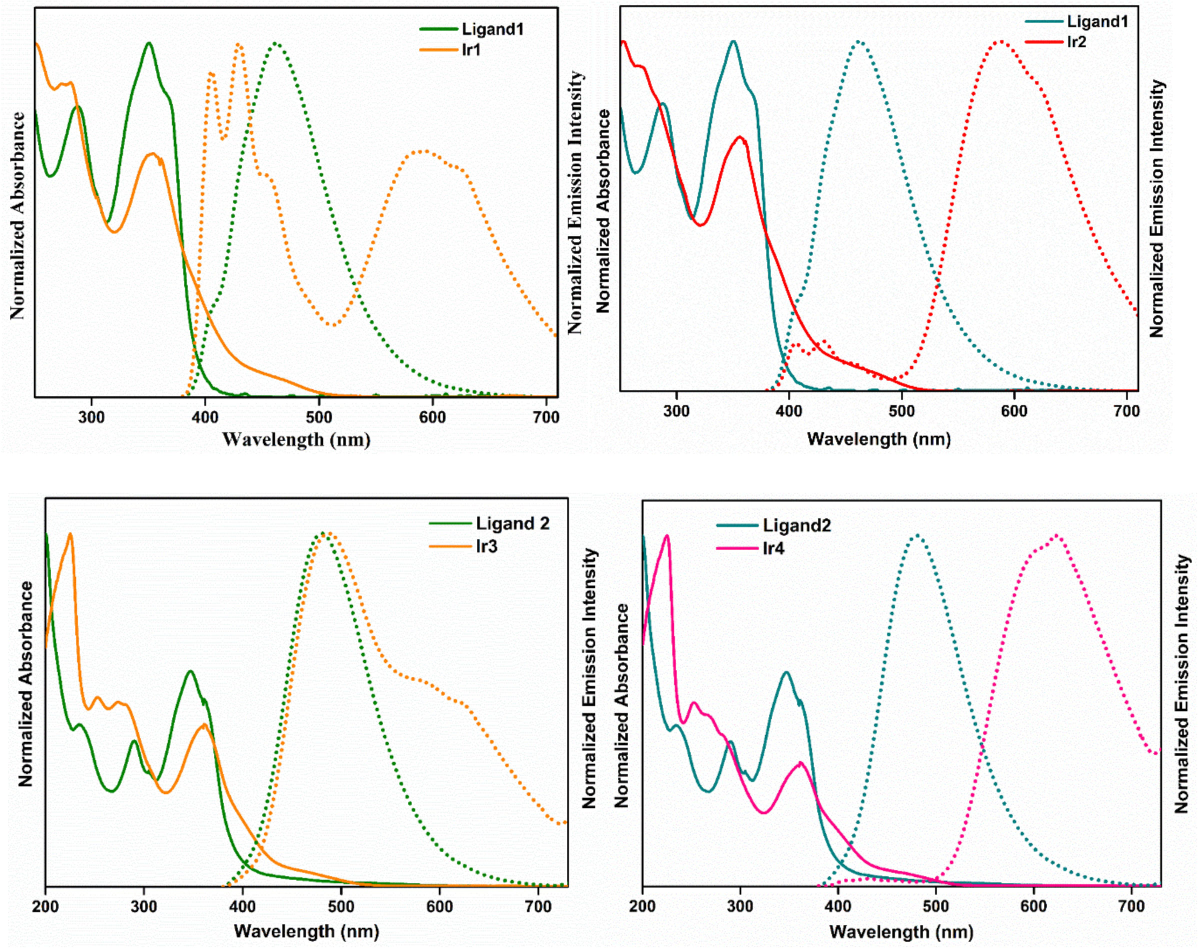

Figure S10. (a-d) Normalized steady-state absorption (solid line) and emission spectra (dotted line) of the free Phenanthroline-BIP ligand (Ligand 1 and 2) and corresponding Ir-complexes (Ir1-Ir4) in DCM with the 365 nm excitation, respectively. The two emission band (I), and (II) represent the MLCT (> 500 nm) and LC (~ 400-480 nm) emissions, respectively.

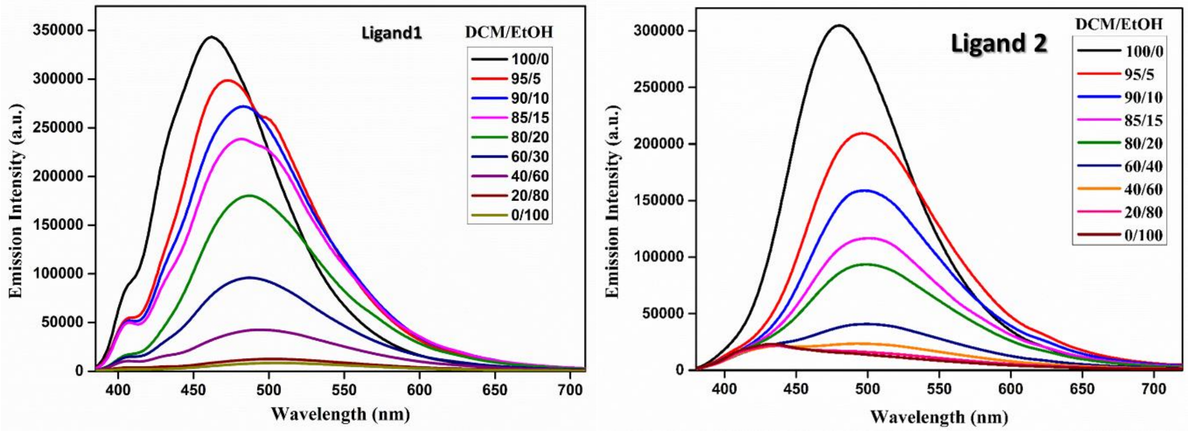

Figure S11. (a-b) Emission spectra of ligand1 and 2 in DCM/EtOH mixtures with various ratios (100/0, 80/20, 60/40, 40/60, 20/80, and 0/100). The excitation wavelength is 375 nm.

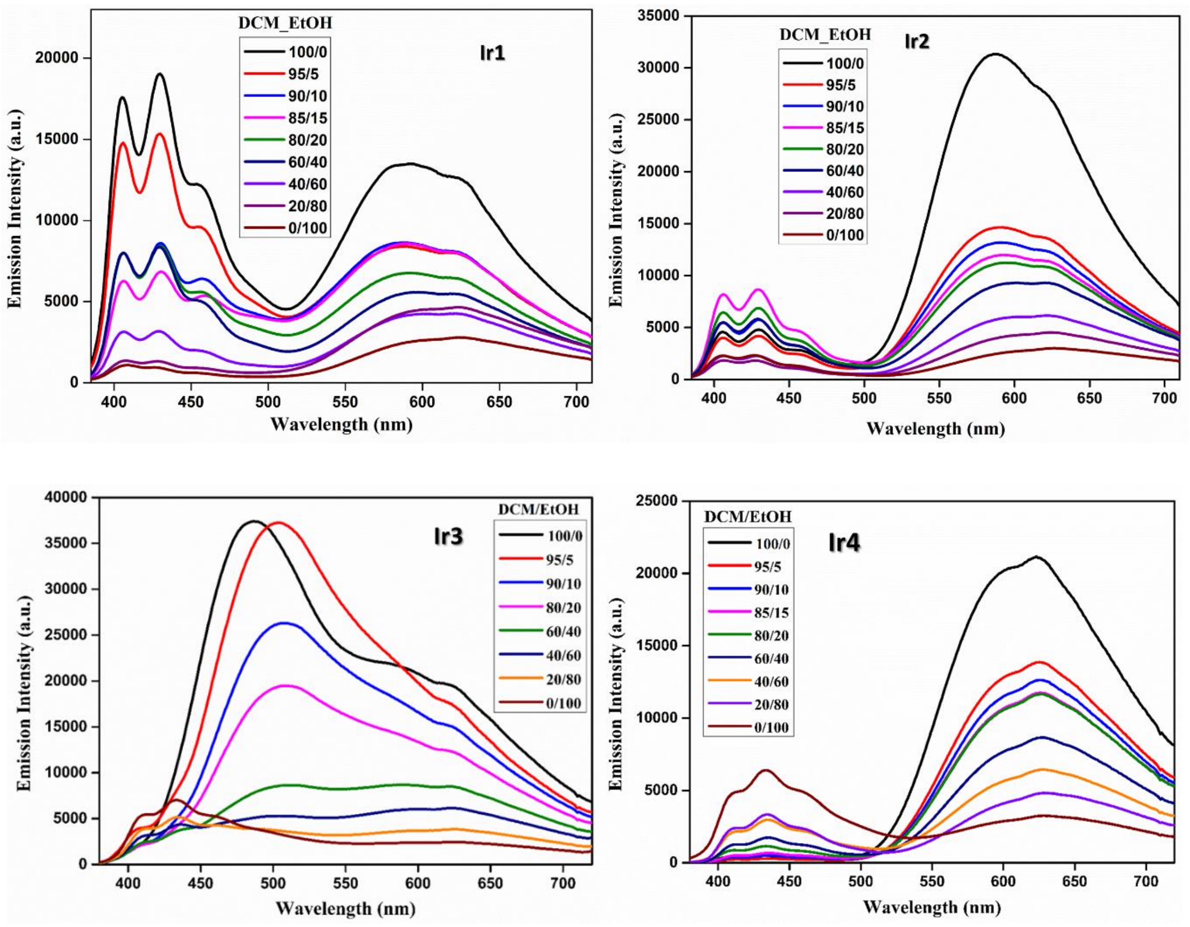

Figure S12. (a-d) Emission spectra of Ir1-Ir4 in DCM/EtOH mixtures with various ratios (100/0, 80/20, 60/40, 40/60, 20/80, and 0/100). The excitation wavelength is 375 nm. The two emission band (I), and (II) represent the MLCT and LC emissions, respectively. When the relative portion of EtOH increases, the band corresponding to the emission of $^3MLCT_{K/E}$ decays. The subscripts E and K refer to the enol (E) and keto (K) forms, respectively.

1.12. Infrared Spectroelectrochemistry (IRSEC)

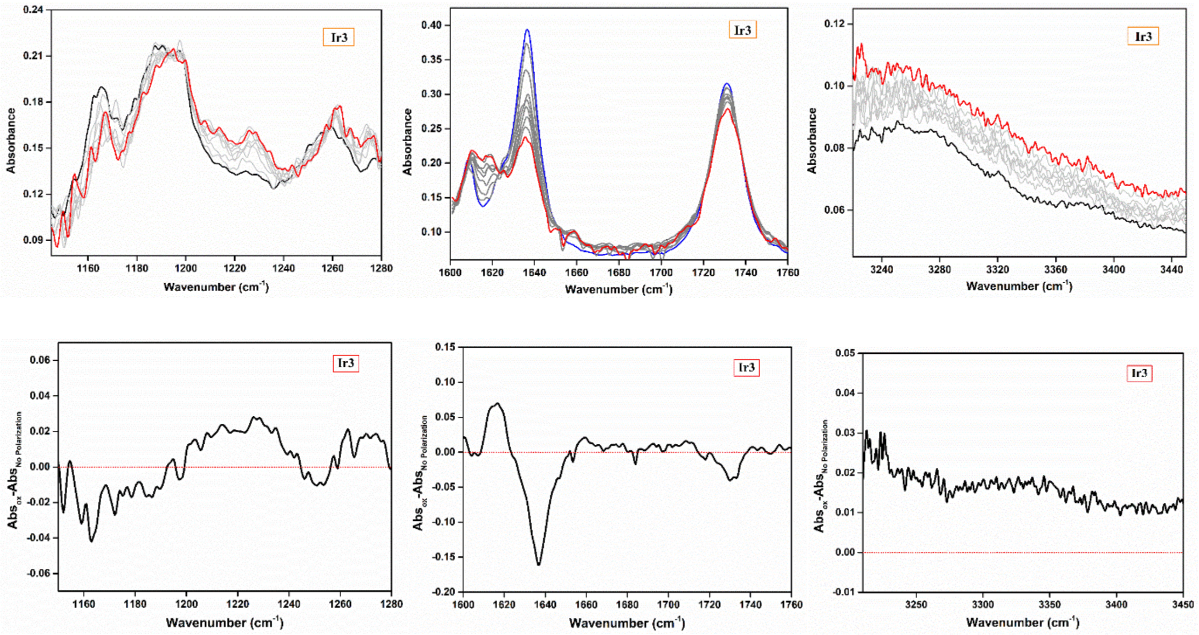

Figure S13. IRSEC spectra of Ir3 (4 M, 500 µL solutions) under oxidative conditions, respectively, in the various regions. Black line corresponds to the spectrum at resting potential (no polarization, ground state spectrum). Red line corresponds to the oxidized species, respectively. Grey lines represent spectra evolution upon polarization. The difference spectra taken from the final spectrum of the oxidized (red line) and the neutral (black line) species. Solvent: $CHCl_3$, 0.1 M nBu_4NPF_6.

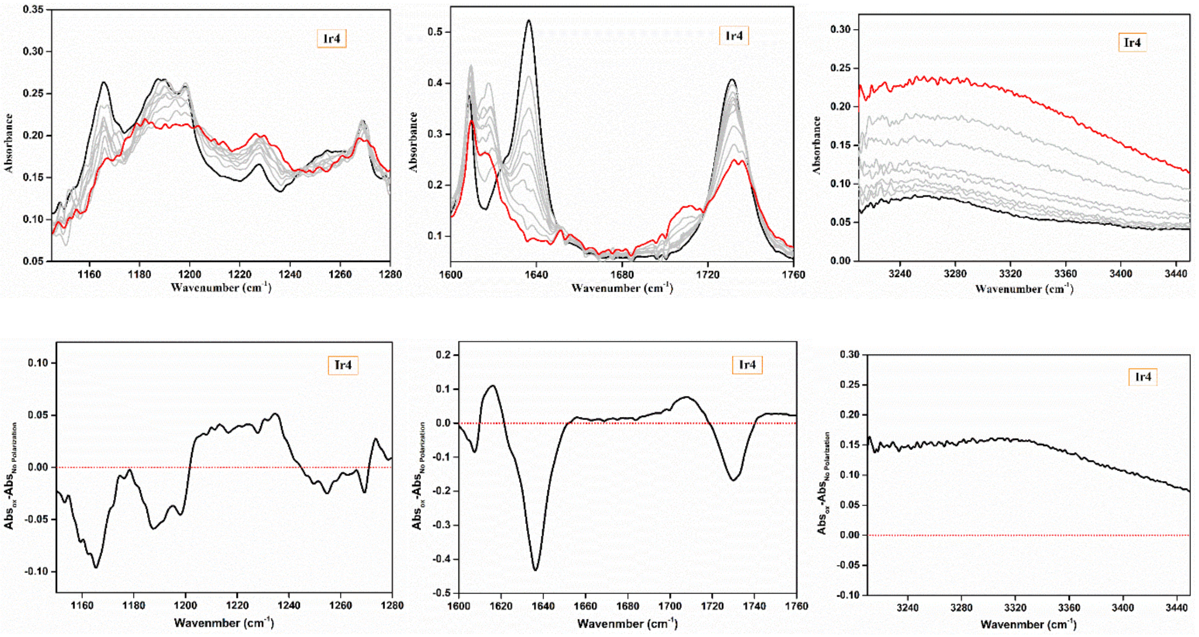

Figure S14. IRSEC spectra of Ir4 (4 M, 500 μL solutions) under oxidative conditions, respectively, in the various regions. Black line corresponds to the spectrum at resting potential (no polarization, ground state spectrum). Red line corresponds to the oxidized species, respectively. Grey lines represent spectra evolution upon polarization. The difference spectra taken from the final spectrum of the oxidized (red line) and the neutral (black line) species. Solvent: $CHCl_3$, 0.1 M nBu_4NPF_6.

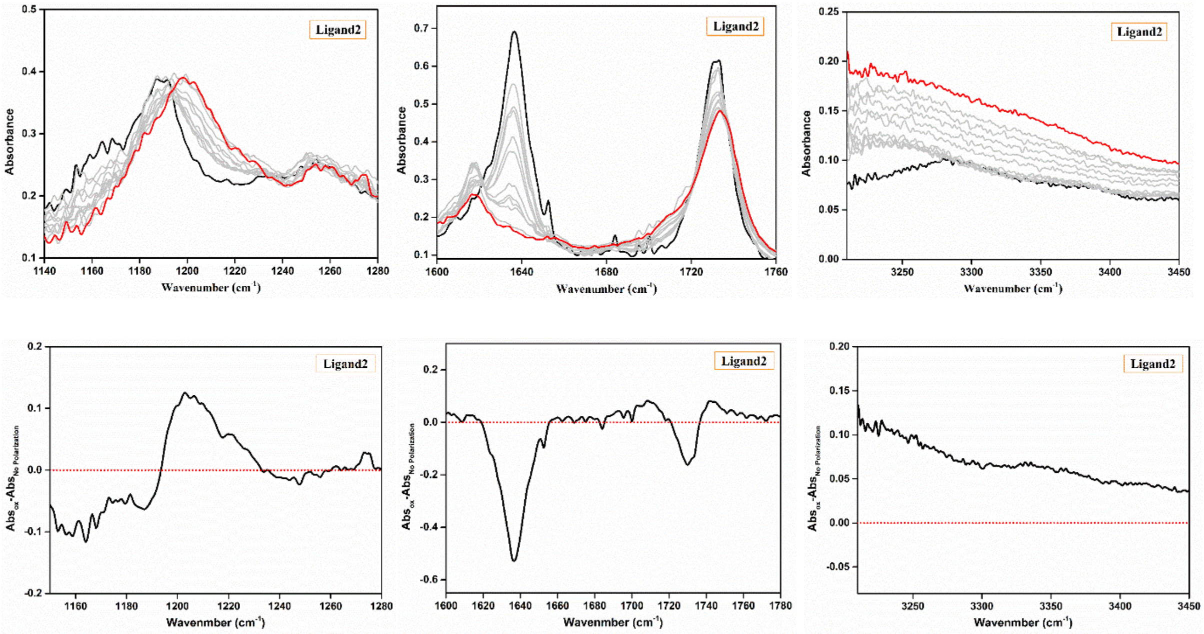

Figure S15. IRSEC spectra of Ligand2 (4 M, 500 μL solutions) under oxidative conditions, respectively, in the various regions. Black line corresponds to the spectrum at resting potential (no polarization, ground state spectrum). Red line corresponds to the oxidized species, respectively. Grey lines represent spectra evolution upon polarization. The difference spectra taken from the final spectrum of the oxidized (red line) and the neutral (black line) species. Solvent: $CHCl_3$, 0.1 M nBu_4NPF_6.

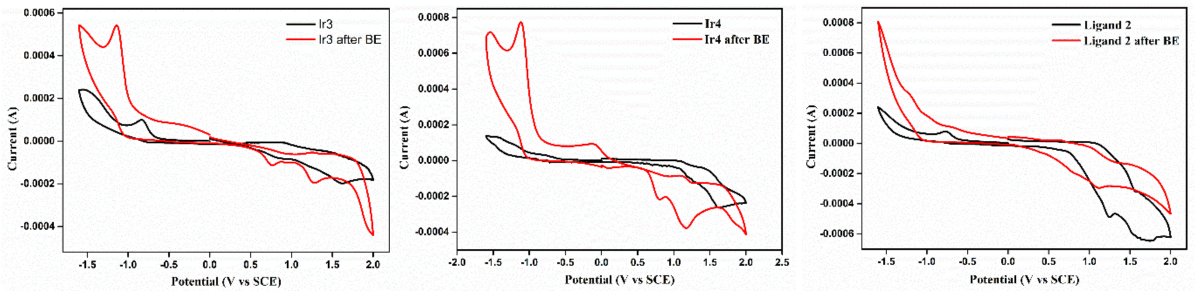

Figure S16. Electrochemical spectra of Ir3, Ir4 and Ligand2 before and after Bulk Electrolysis in $CHCl_3$, 0.1 M nBu_4NPF_6 recorded during IRSEC experiment.

1.13. Solvent-Modulated ESIPT and PCET Pathways[4]

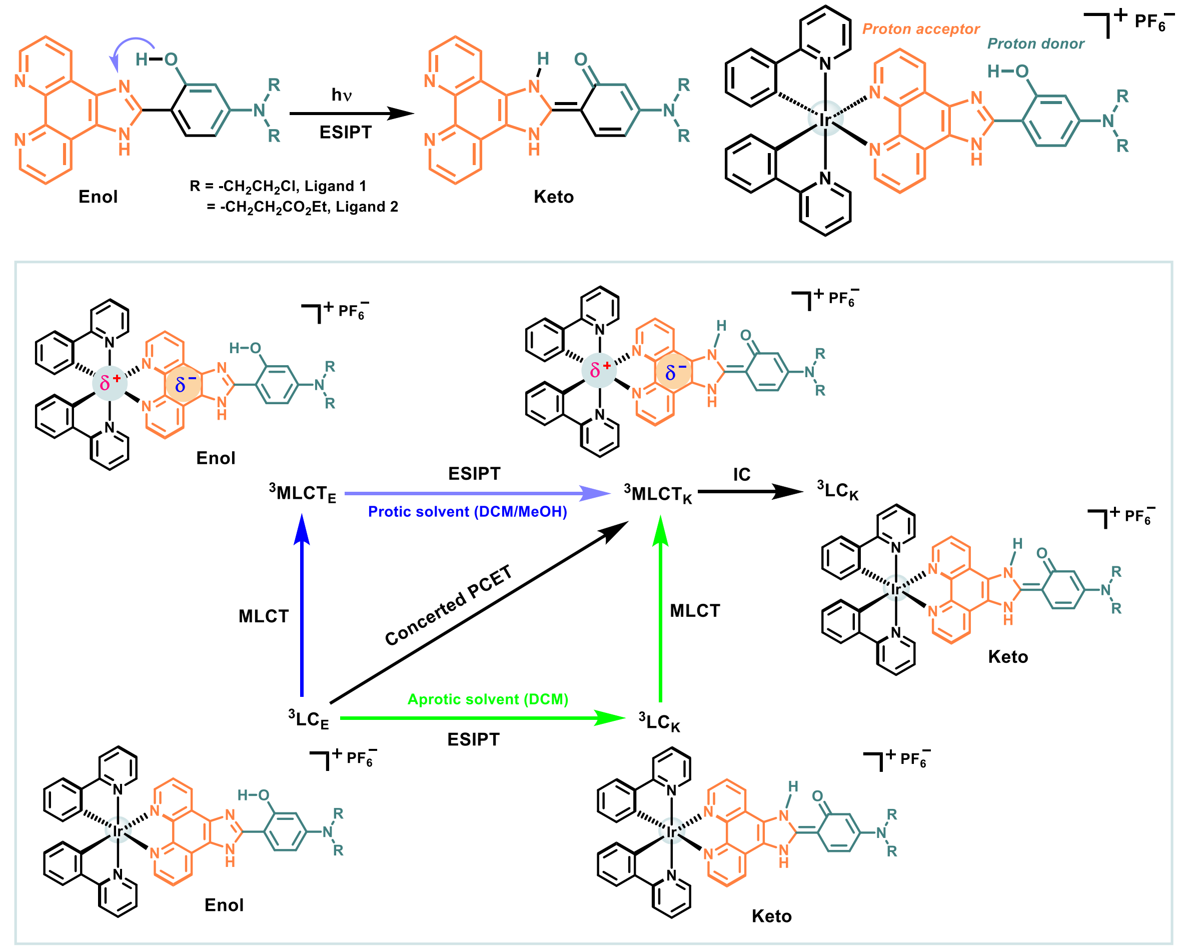

Figure S17. Molecular structure of [Ir(III)-phenanthroline-imidazole-phenol] complexes. The ESIPT occurs in the free ligands. The proton-accepting part and donating parts are marked in orange and blue, respectively. Schematic representation of three possible pathways of PCET. The stepwise ET-PT pathway and stepwise PT-ET pathway are marked in blue and green, respectively.[5] The concerted PCET pathway is marked in black. LC and MLCT represent ligand-centered and metal-to-ligand charge transfer states, respectively. The subscripts E and K refer to the enol and keto forms, respectively. The superscripts indicate the spin multiplicities.

1.14. Singlet Oxygen Quantum Yields

The singlet oxygen quantum yield (Φ_Δ) is a term used to measure the efficiency between the absorbed photons and generated singlet oxygen ($^1O_2$) of a photosensitizer (PS). The Φ_Δ of Ir1, Ir2, Ir3, and Ir4 were determined using the relative method by a standard 1,3-diphenylisobenzofuran (DPBF) photodegradation study. The Φ_Δ were calculated by comparison with the literature reported standard methylene blue (MB, Φ_Δ = 0.52 in H_2O) according to the equation given below, while MB was measured with the same excitation light source, in the same solvent and with the same number of measurement runs. Excitation was performed using a 405 nm LED light source at a distance of 6.0 cm from the cuvette. Where, K is the slope of photodegradation of DPBF vs time (s) plot.

$$\Phi_{\Delta(\mathrm{PS})} = \Phi_{\Delta(\mathrm{MB})} \times \left(\frac{K_{PS}}{K_{MB}}\right) \ldots\ldots\ldots (5)$$

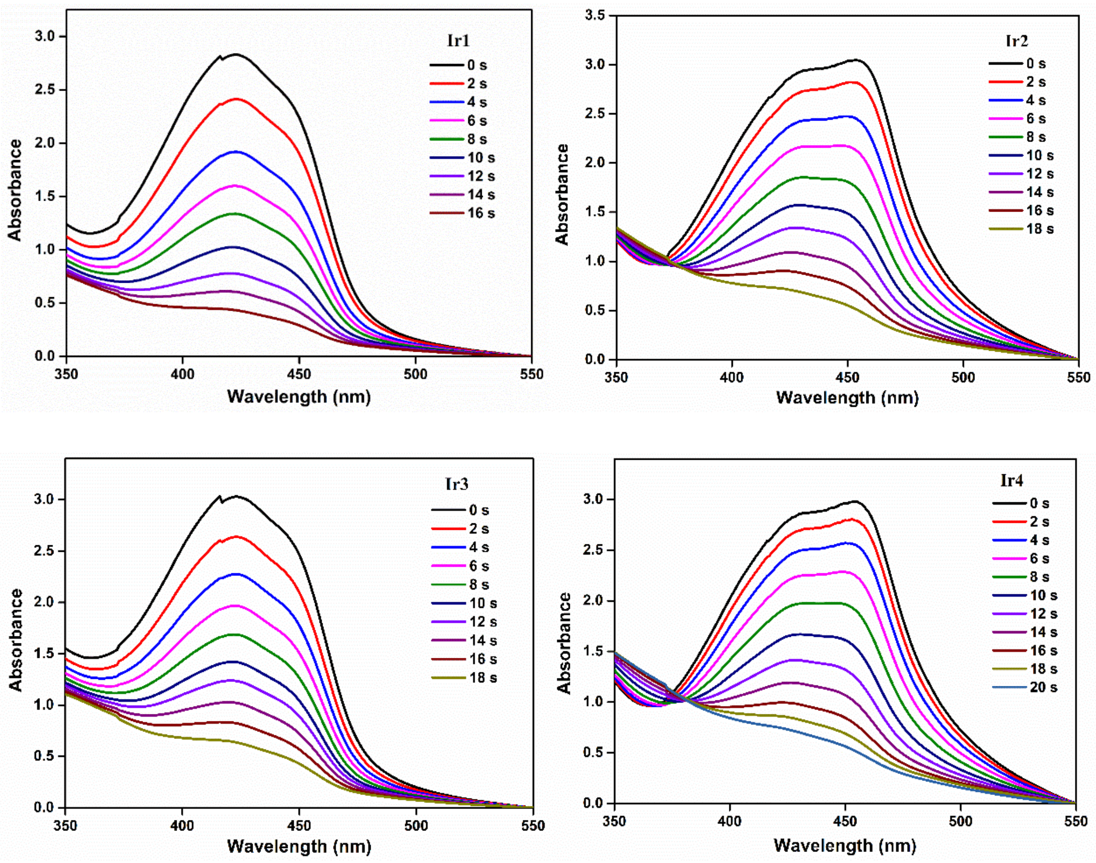

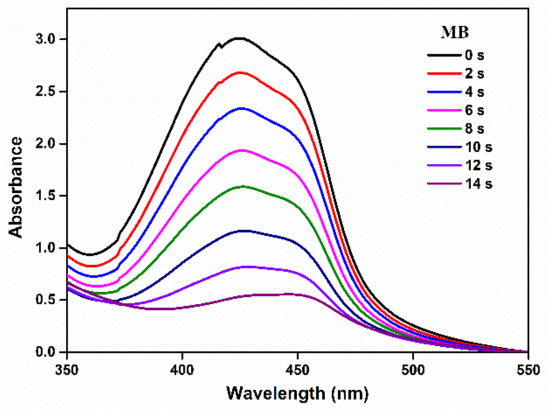

Figure S18. Change in the electronic absorption spectrum of a mixture of DPBF (initial concentration = 100 μM) and (a) Ir1, (b) Ir2, (c) Ir3, (d) Ir4, or (e) MB (10 μM) in PBS solution (pH = 7.4, 1% DMSO) under light illumination (λ_{ex} = 405 nm) at 298 K at different times.

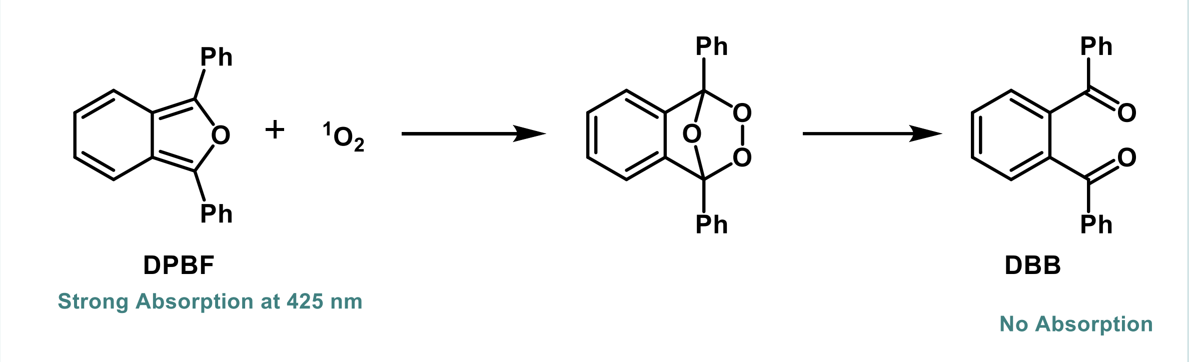

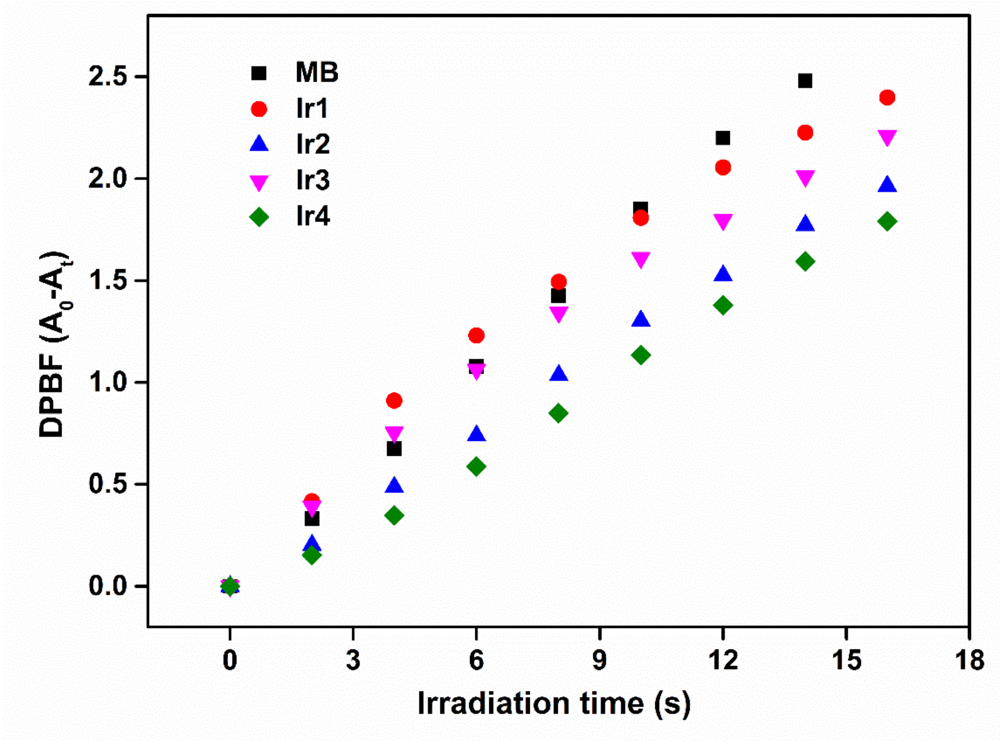

Figure S19. A) The mechanism of 1,3-diphenylisobenzofuran (DPBF) as the $^1O_2$ scavenger monitors singlet oxygen generation in the solution. B) Determination of the singlet oxygen quantum yield of Ir(III)-complexes in phosphate-buffered solutions containing 1% DMSO. Plots of the changes in absorption ΔAbs (A_0-A_t) of DPBF in dependence of the irradiation time in the presence of Ir(III)-complexes or Methylene blue (MB, as reference 0.52 in water). All spectra were determined upon irradiation with a LED light (λ = 405 nm).

Table S6. The singlet oxygen quantum yields (Φ_Δ) of Ir1, Ir2, Ir3, and Ir4 in 1% DMSO PBS buffer solution upon irradiation with light of 405 nm.

**Ir(III)-Complex**	**Φ_Δ**
**Ir-1**	0.43
**Ir-2**	0.36
**Ir-3**	0.39
**Ir-4**	0.33

1.15. ESR measurements

ESR measurements were carried out at room temperature on a Bruker Elexsys 580 instrument. The TEMP and DMPO were used to detect $^1O_2$ and $^{\bullet}OH$, respectively. In an EPR tube 10 μL TEMP (10 mM) or DMPO (10 mM) was mixed with 10 μL Ir2 (10^{-4} M) + 10 μL GSH (10 mM) and irradiated by blue LED light (405 nm) for 3-5 min. As a comparison, the Ir2 mixed with TEMP or DMPO in the dark was detected as well.

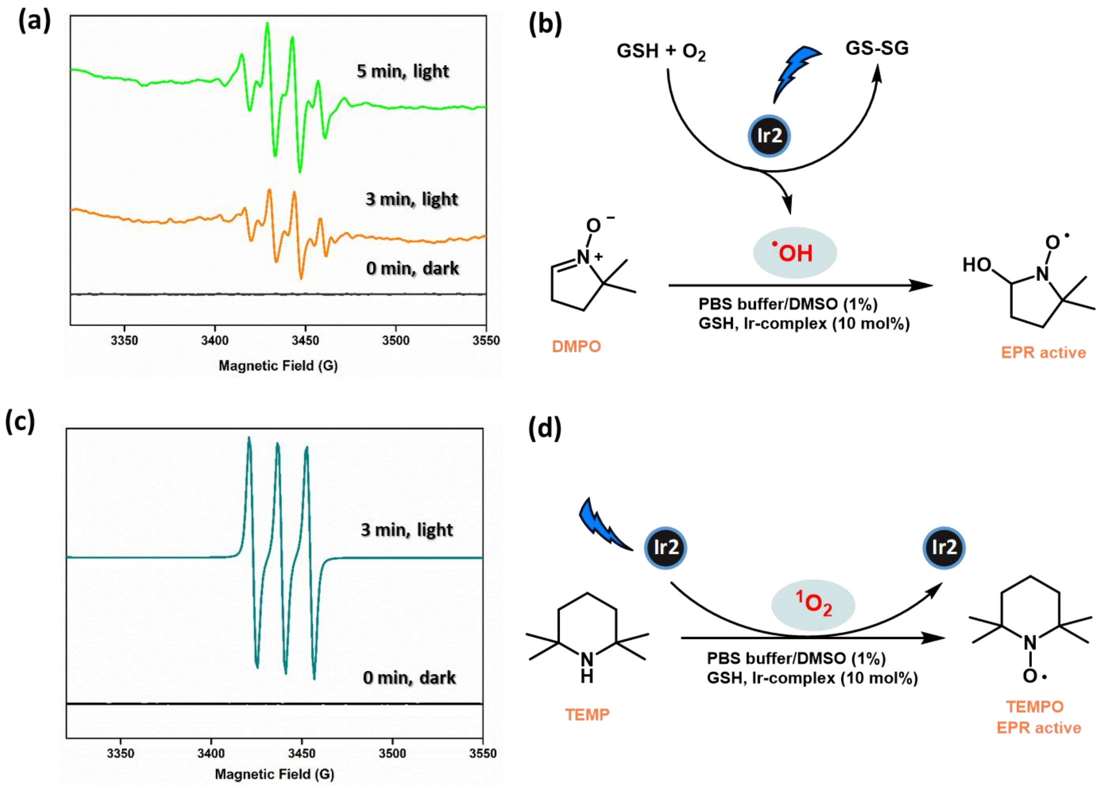

Figure S20. (a) The characterization of $^{\bullet}OH$ by ESR spectra using DMPO as a spin trap. (b) The mechanism of DMPO as the spin trapper to detect the $^{\bullet}OH$. (c) ESR spectra to detect $^1O_2$ generated using TEMP as a spin trap. (d) The mechanism of TEMP as the spin trapper to detect the $^1O_2$.

1.16. Methylene Blue Assay for Extracellular $^{\bullet}OH$ Detection

To detect the $^{\bullet}OH$ generated by Ir-complexes under photocatalytic reaction, MB probe was used for verification. 10 μL MB (0.8 mg/mL) solution was respectively added into 3 mL Ir2 (10 μM) without or with 10 μL GSH (3.5 mg/mL) treatment. Afterward, the mixtures were irradiated with blue LED light at different time intervals. The corresponding changes in the UV absorption values of MB (650 nm) were measured.

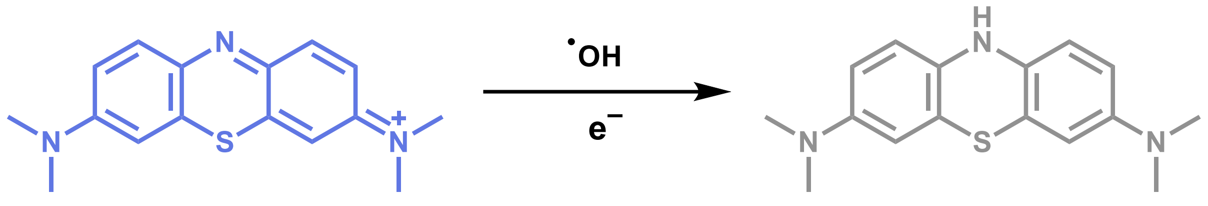

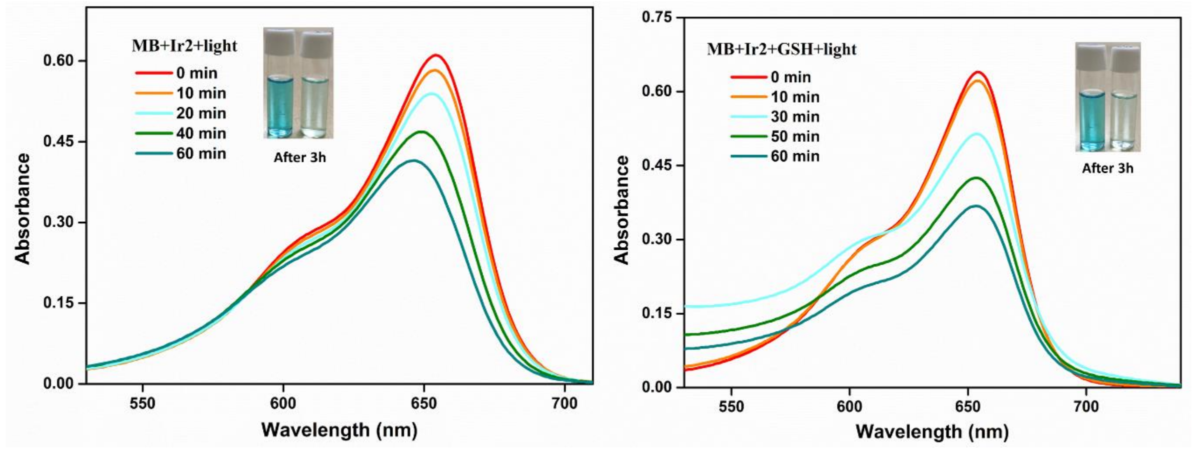

Figure S21. Illustration of MB reaction with $^{\bullet}OH$. UV-Vis absorption spectra of MB degradation assay for $^{\bullet}OH$ evaluation treated with Ir2+light, and the Ir2+GSH+light in PBS solution, photograph (inset) of MB solution after 3h of exposure to light.

1.17. Two-photon Absorption Cross Section

Two techniques were used to measure two-photon absorption cross section (δ_{2PA}), namely, two-photon excited fluorescence (2PEF) and Z-scan methods. Two-photon luminescence measurements were performed in fluorometric quartz cuvettes. The experimental luminescence excitation and detection conditions were conducted with negligible reabsorption processes, which can affect TPA measurements. The quadratic dependence of two-photon induced luminescence intensity on the excitation power was verified at different excitation wavelength ranges of 720–840 nm. The two-photon absorption cross section of the probes was calculated at each wavelength using relative to Rhodamine-6G reference according to equation (3):

$$\delta_1 = \delta_1 \frac{\emptyset_1 C_1 I_2 n_2}{\emptyset_2 C_2 I_1 n_1} \dots\dots\dots (6)$$

Where I is the integrated luminescence intensity, C is the concentration, n is the refractive index, and Φ is the quantum yield. Subscript '1' stands for reference samples, and '2' stands for samples. (Göppert-Mayer, 1 GM = 10^{-50} cm^4 s^{-1} photon^{-1})

Using a single-beam open-aperture (OA) z-scan technique, we performed the nonlinear optical measurement of all Ir-complexes. A Ti: Sapphire laser giving 100 fs pulses with 1 W average power and a focused beam spot size of 50 μm was used for the OA z-scan technique. OA z-scan measurements were carried out on 10^{-4} M solutions of Ir-complexes in DMSO solvent at different wavelengths, ranging from 720–840 nm (Figure S16). The experimental OA curves were then fitted with the transmission equation, including two-photon absorption (TPA).[6]

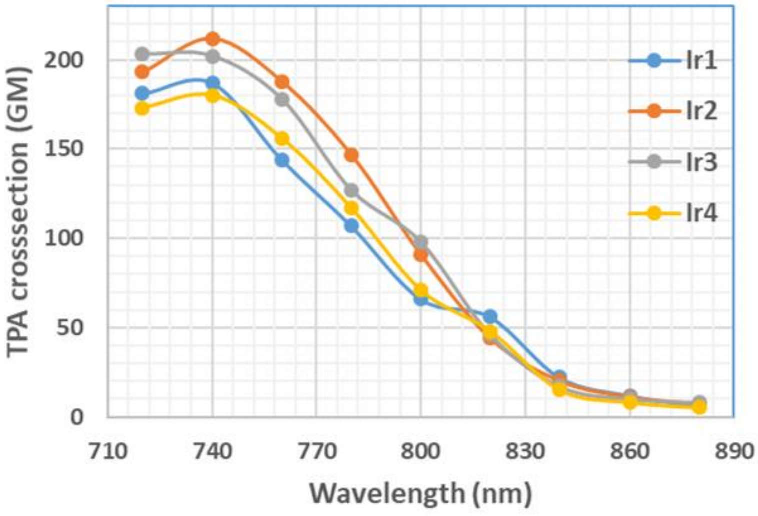

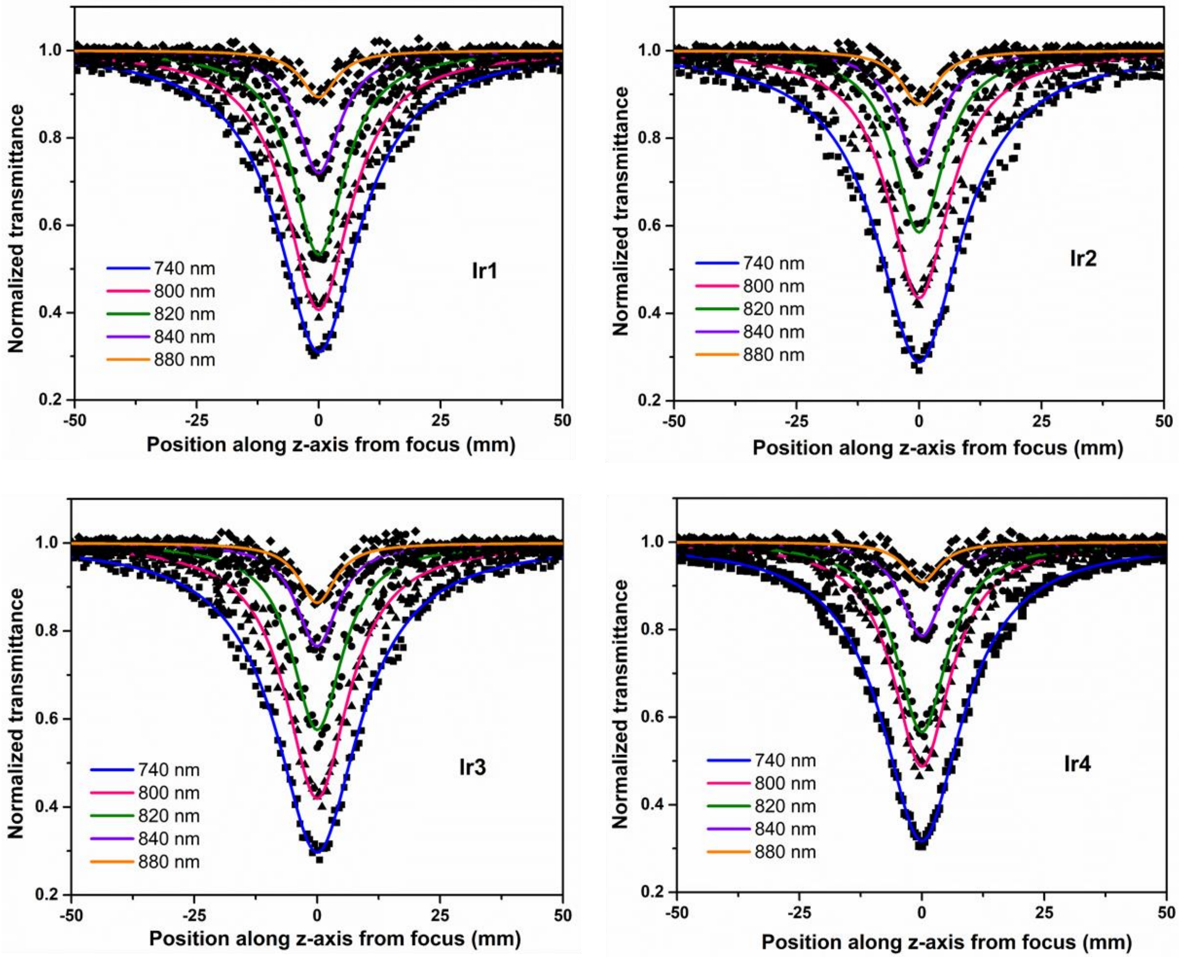

Figure S22. Two-photon absorption (TPA) cross-sections (δ_{2PA}) of Ir1-Ir4 complexes in DMSO at excitation wavelengths between 720–840 nm. The open-aperture (OA) z-scan curves were recorded at different wavelengths using a 10^{-4} M solution of Ir1-Ir4 complexes.

1.18. Photocatalytic oxidation of NADPH by UV-visible spectroscopy

Ir-complexes were evaluated for the catalytic oxidation of NADH to NAD^+ by UV-visible spectroscopy in the dark and under blue LED light irradiation (405 nm) at 298 K. The Ir concentration remained fixed at 10 μM and the NADH concentration was about 200 μM. The conversion of NADH to NAD^+ was followed by absorption at 339 nm (ε(NADH) = 6220 cm^{-1} M^{-1}) to allow evaluation of kinetic data. The catalytic turnover number (TON) was calculated using the following equations (1) and (2):

$$[NAD^{+}] = \frac{Abs(339\ nm)initial - Abs(339\ nm)final}{Abs(339\ nm)initial} \times [NADH] \quad \ldots\ldots (7)$$

$$Turnover\ number\ (TON) = [NAD^{+}] \div [Catalyst] \quad \ldots\ldots (8)$$

The turnover frequency (TOF) of photocatalytic oxidation of NADH was calculated from the difference in NADH concentration after 1 min irradiation divided by the concentration of photosensitizer.

$$Turnover\ frequency\ (TOF) = TON \div Time\ (\text{min}\ or\ h) \quad \ldots\ldots (9)$$

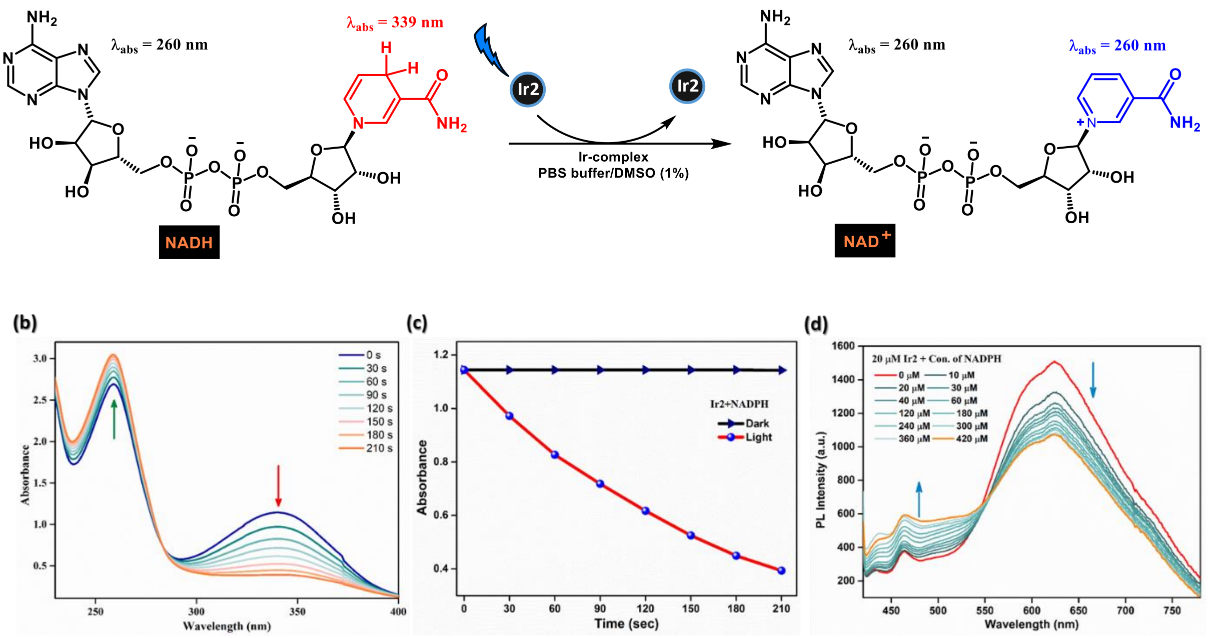

Figure S23. Oxidation of NADH by Ir2. (a) Schematic illustration of the photocatalytic NADH/NAD^+ transformation. (b) The UV-Vis absorption spectra for the photocatalytic oxidation of NADH (200 μM) by Ir2 (10 μM) under light irradiation in PBS solution at 298 K. (c) Time dependence of Absorbance at 339 nm of NADH in under light irradiation or in the

dark. Light irradiation: 405 nm LED. (d) Fluorescence quenching experiment of Ir2 (20 μM) with changing the NADPH concentration (0-420 μM) were also done in MeOH solution.

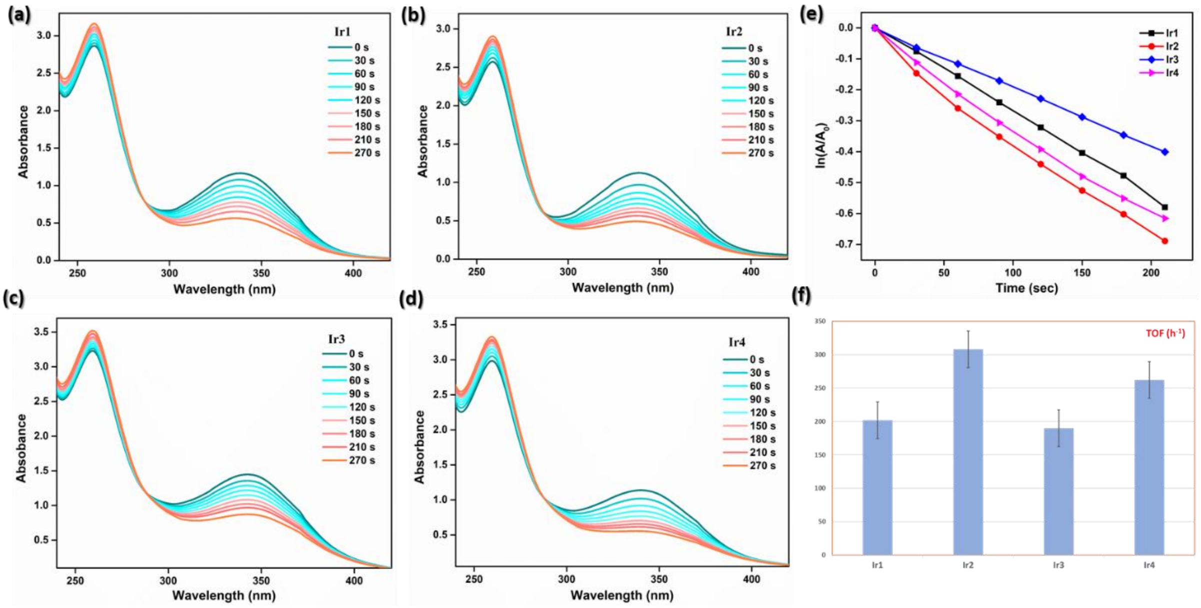

Figure S24. (a)-(d) The study of photocatalytic oxidation of NADH (*ca.* 200 μM) by Ir1-Ir4 complexes (10 μM) through the change of UV-Vis absorption spectra under blue LED light irradiation in PBS solution at 298 K. (e) The kinetic plot of ln(A/A_0) vs Time (s) at 339 nm of NADH under light irradiation of all complexes. (f) Comparison of TOF values of Ir1-Ir4 complexes.

1.19. Photocatalytic Oxidation of NADPH by ^{1}H NMR Spectra

β-Nicotinamide Adenine Dinucleotide Disodium Salt (Reduced) (β-NADH.Na_2) (3.5 mM) was added to an NMR tube containing 0.25 mM of Ir2 in CD_3OD/D_2O (2:1). ^{1}H NMR spectra of the resulting solutions were recorded at 298 K after 30 min and 45 light irradiation (405 nm, 13 mW/cm^2) or in the dark.

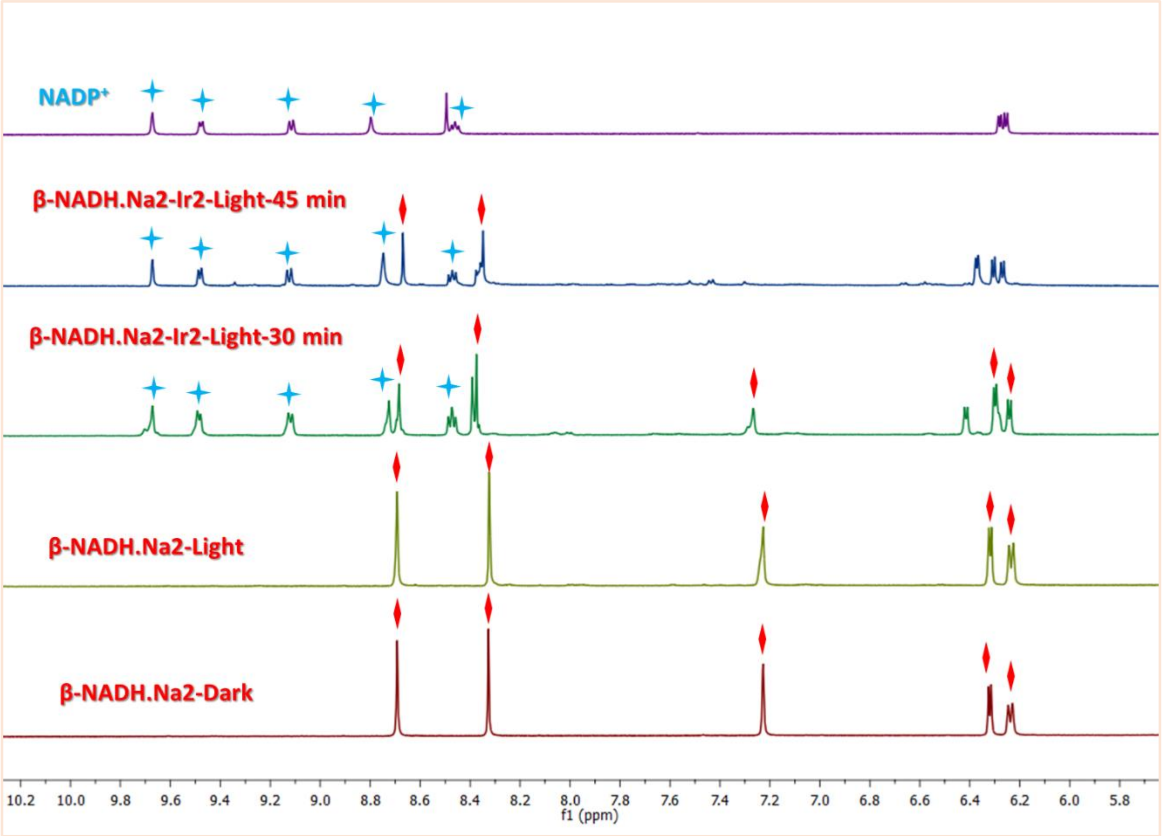

Figure S25. Photocatalytic oxidation of β-Nicotinamide Adenine Dinucleotide Disodium Salt (Reduced) (β-NADH.Na2) (3.5 mM) by Ir2 (0.25 mM) under dark or irradiation conditions monitored by ^{1}H NMR spectroscopy at 298 K. Peaks associated with red squares represent NADH, those with blue stars represent NAD^+.

1.20. Photocatalytic Performances and Mechanism of Ir2-complex

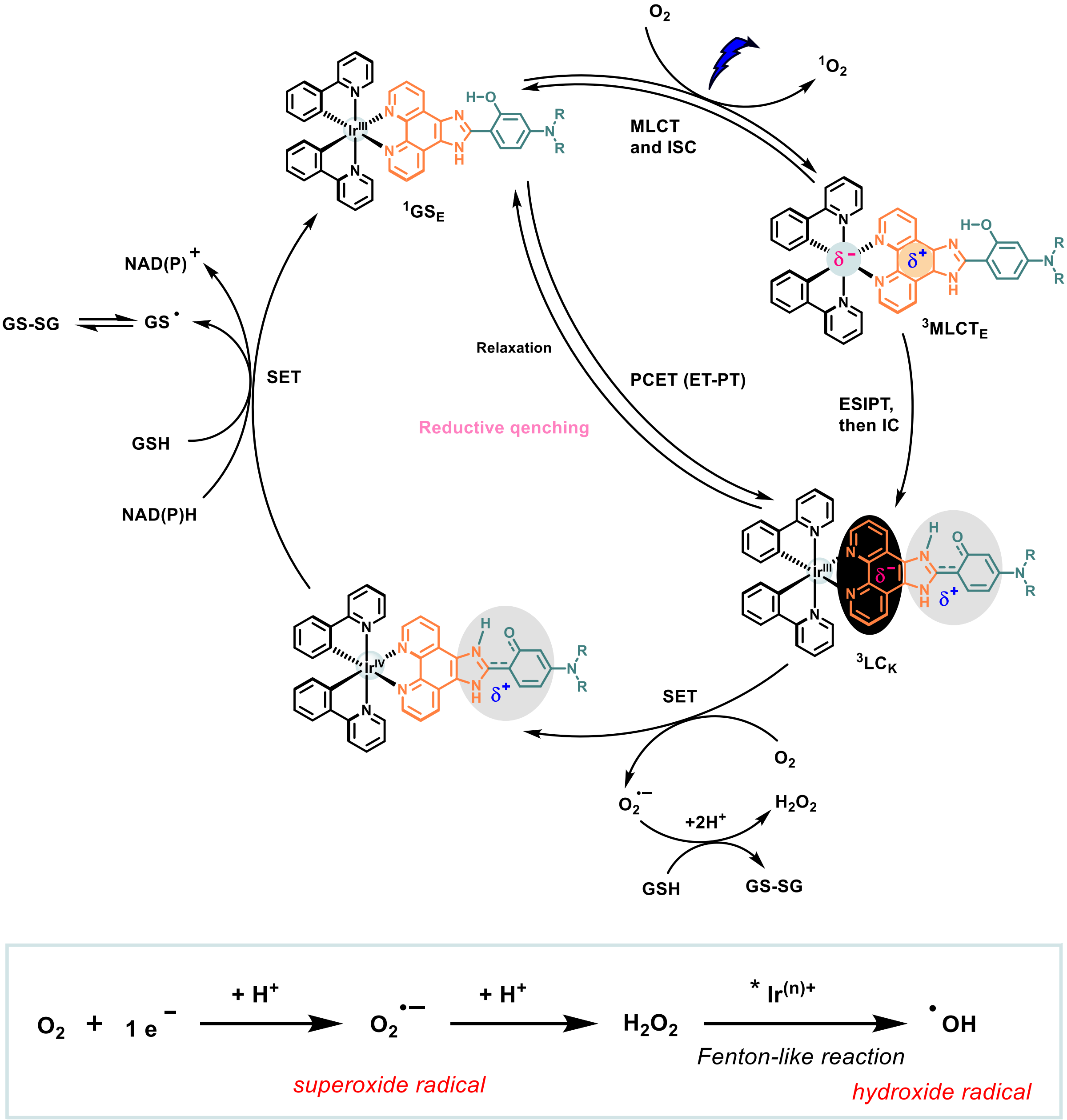

Figure S26. Proposed photocatalytic cycle for molecular oxygen reduction and oxidation of NAD(P)H or GSH with Ir2-complex *in cellulo*.

1.21. Depletion of GSH in solution

The consumption of GSH was monitored by UV-Vis spectroscopy. Ir2 (20 µM) was mixed with different concentrations of GSH (0-200 µM) in PBS solution at room temperature. Then the absorbance spectrum of the solution was measured by UV-vis spectroscopy. Apart from UV-Vis spectroscopy, a fluorescence quenching experiment of Ir2 (20 µM) with changing the GSH concentration (0-700 µM) was also done in MeOH solution.

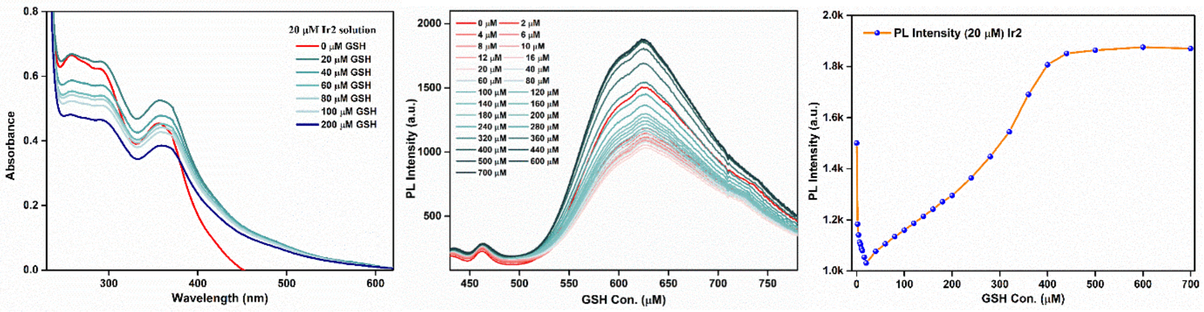

Figure S27. (a) The consumption of GSH was monitored by UV-Vis spectroscopy Ir2 (20 µM) was mixed with different concentrations of GSH (0-200 µM) in PBS solution at 298 K. (d) Fluorescence quenching experiment of Ir2 (20 µM) with changing the GSH concentration (0-700 µM) was also done in MeOH solution. (c) Change in PL intensity of Ir2 with changing the GSH concentration.

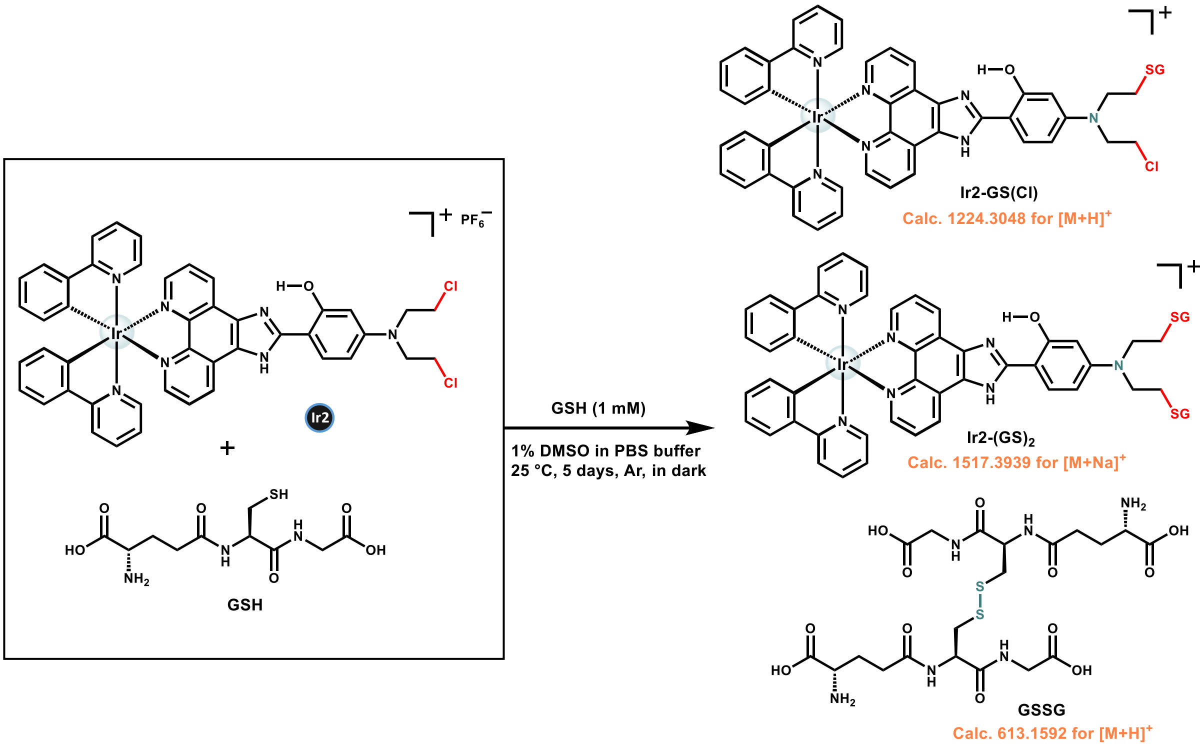

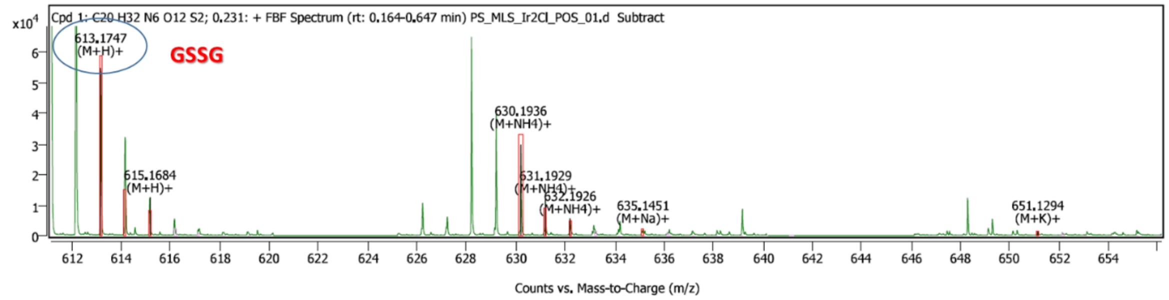

Compound ID Table

Name	Formula	Species	RT	RT Diff	Mass	CAS	ID Source	Score	Score (Lib)	Score (Tgt)
	C20 H32 N6 O12 S2	(M+H)+ (M+NH4)+ (M+Na)+ (M+K)+	0.231		612.1632		FBF	38.36		38.36

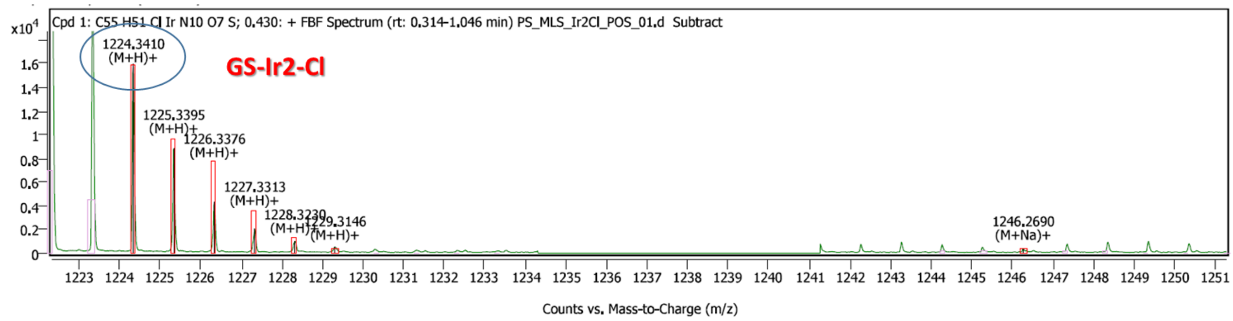

Compound ID Table

Name	Formula	Species	RT	RT Diff	Mass	CAS	ID Source	Score	Score (Lib)	Score (Tgt)
	C55 H51 Cl Ir N10 O7 S	(M+H)+ (M+Na)+	0.430		1221.3278		FBF	17.43		17.43

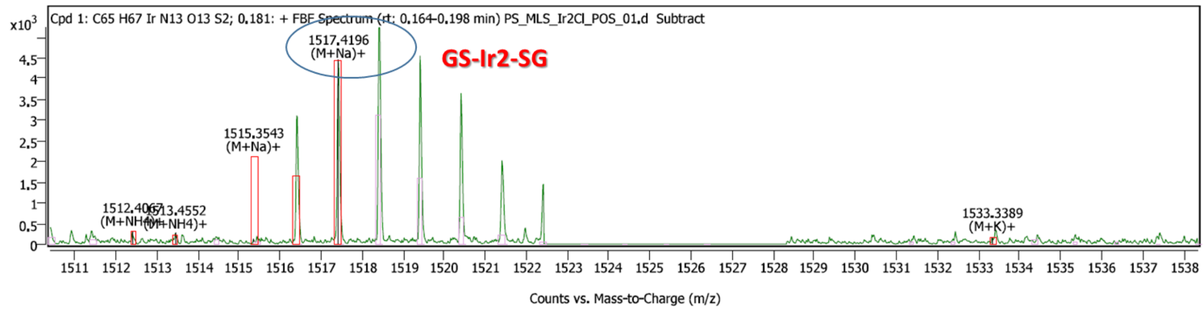

Compound ID Table

Name	Formula	Species	RT	RT Diff	Mass	CAS	ID Source	Score	Score (Lib)	Score (Tgt)
	C65 H67 Ir N13 O13 S2	(M+NH4)+ (M+Na)+ (M+K)+	0.181		1492.4211		FBF	16.91		16.91

Figure S28. Schematic illustration for the reaction of Ir2 bearing electrophilic *N*,*N*-bis(2-chloroethyl)-azane group with GSH in 1% DMSO/PBS buffer. HRMS analysis of the reaction mixture containing Ir2 complex (100 μM) and GSH (1 mM) in the dark for 5 days.

2. *In vitro* study:

2.1. Cell culture

Mouse breast cancer cells (4T1) and human breast cancer cell line (MCF-7) were cultured in DMEM medium, supplemented with 10% fetal bovine serum (FBS) (Gibco) and (1%, v/v) penicillin-streptomycin. Cells under normoxia were cultured in 5% CO_2 and 21% O_2 incubator at 37 °C; cells under hypoxia were cultured in 5% CO_2 and 1% O_2 incubator at 37 °C. Oxygen indicator was used to detect the oxygen levels in the chamber (1% O_2).

2.2. Photo-toxicity and dark-toxicity *in vitro*

The *in vitro* cell cytotoxicity of Ir1-Ir4 complexes to tumour cells under different oxygen levels were evaluated on 4T1 and MCF-7 cells using a standard MTT assay. 4T1 or MCF-7 cells were seeded into 96-well plates with a density of 1×10^4 per well and culture in hypoxia (1% O_2), or normoxia (21% O_2) environment for 24 h, respectively. The cells were incubated with different concentrations of Ir1-Ir4 complexes for 8 h under hypoxia or normoxia. After that, the culture media was replaced by fresh culture media, which did not contain the complex. The 96-well plates of the dark groups were kept in the dark for 1 h. The 96-well plates of the light groups were irradiated by light (405 nm, 20mW/cm^2, 10 min). Afterward, upon further incubation for 40 h, the MTT solution (10 μL, 5 mg mL^{-1}) was added to each well for an additional 4 h. The medium containing MTT solution was discarded, and dimethyl sulfoxide (DMSO, 200 μL per well) were added to dissolve formazan crystals for 30 min. Finally, the microplate reader was used to measure the absorbance at 490 nm. The wells containing cells incubated without complex were set as control. The cell viability rate (VR) was calculated according to the equation: $VR = (A-A_0)/(A_S-A_0) \times 100\%$, where A is the absorbance of the experimental group, A_S is the absorbance of the control group and A_0 is the absorbance of the blank group (no cells).

The cells were subject to different treatments: (i) control group (with only PBS); (ii) only light irradiation group (only PBS, 405 nm, 20 mW/cm^2, 10 min); (iii) only Ir2 group (without light); (iv) Ir2+light irradiation group (405 nm, 20 mW/cm^2, 10 min).

2.3. Cellular colocalization imaging

4T1 cells were incubated with Ir1-Ir4 complexes (2 μM) for 4 h at 37 °C. Then co-incubated with LysoTracker Green (20 μM) or MitoTracker Green (20 μM) for 30 min. The cells were rinsed twice with PBS and imaged with confocal laser scanning microscope immediately. (Ir complexes, λ_{ex}: 405 nm), (MitoTracker Green, $\lambda_{ex}/\lambda_{em}$ = 488/550 nm) or (LysoTracker Green, $\lambda_{ex}/\lambda_{em}$ = 488/550 nm).

2.4. Flow cytometry analysis Apoptosis

Flow cytometry was used to investigate the mechanism of cell death of 4T1 cells under hypoxic conditions using an Annexin V-APC/7-AAD Apoptosis Detection Kit. 4T1 cells were seeded in six-well plates for 24 h under hypoxia and incubated with Ir-2 complexes (2 μM, at 37 °C) for 4 h. Cells were irradiated with 405 nm light, 20 mW/cm^2, 10 min. Apoptosis assay was performed 0.5 h after irradiation. The medium was removed and the cells were washed with PBS buffer twice. After that, 500 μL of Annexin V binding buffer containing 5 μL Annexin V-APC and 5 μL 7-AAD were added. After incubation for 10 min in the dark at room temperature, Flow cytometry were done.

2.5. Detection of intracellular ROS generation

4T1 cells were seeded on confocal dishes (1 × 10^5 cells/dish) and incubated for 24 h under a normoxic (21% O_2) or hypoxic (1% O_2) environment. The cells were treated with Ir2 complexes (2 μM, 1% DMSO, v%) for 4 h and then the cells were washed with PBS for three times. Next, the serum-free RPMI medium containing DCFH-DA (20 μM) was added and incubated for another 30 min. After all treatments, cells were irradiated with 405 nm light, 20 mW/cm^2, 10 min under different oxygen concentrations. The cells were rinsed with PBS for three times and the medium was replaced with fresh one before visualization by a laser fluorescent confocal microscope (DCF, $\lambda_{ex}/\lambda_{em}$ = 488/525 nm).

2.6. Intracellular lipid peroxides measurement

For LPO detection, BODIPY$^{665/676}$ probe was used that can be oxidized by intracellular LPO. 4T1 cells were seeded on confocal dishes (1 × 10^5 cells/dish) at 37 °C and incubated for 24 h

under hypoxia. The cells were incubated with Ir-2 complexes (2 μM, at 37 °C) for 4 h; then the cells were washed with PBS and irradiated with 405 nm light, 20 mW/cm^2, 10 min. After that, cells were incubated with 20 µM BODIPY$^{665/676}$ for 30 min. Finally, cells were imaged in the laser fluorescent confocal microscope. (BODIPY$^{665/676}$, $\lambda_{ex}/\lambda_{em}$ = 633/676 nm).

2.7. Analysis of mitochondrial membrane potential (MMP)

The mitochondrial membrane potential was measured by using JC-1 probe. 4T1 cells were seeded on confocal dishes (1 × 10^5 cells/dish) at 37 °C and incubated for 24 h under hypoxia. The cells were incubated with Ir-2 complexes (2 μM, at 37 °C) for 4 h followed by irradiated with 405 nm light, 20 mW/cm^2, 10 min. JC-1 staining assay was performed 0.5 h after irradiation. The medium was removed and the cells were washed with PBS. The cells were stained with JC-1 (50 nM) at 37 °C for 30 min. The medium was replaced with fresh one and the fluorescence was recorded by using a laser fluorescent confocal microscope, Excitation/emission: green 488/530 nm and red 535/610 nm.

2.8. Cellular GSH detection

GSH detection was investigated in 4T1 cells by using GSH and GSSG assay kits. 4T1 cells were incubated on confocal dishes (1 × 10^5 cells/dish) at 37 °C for 24 h followed by the treatment with Ir-2 complexes (2 μM, at 37 °C) in the dark for 4 h incubation. The supernatant was replaced with a fresh culture medium and cells were subjected to irradiation (405 nm light, 20 mW/cm^2, 10 min). Cells without irradiation were replaced with a fresh culture medium and maintained in the dark. After 0.5 h of the additional incubation, and washed twice with PBS. Then the manufacturer's protocol was followed.

2.9. Western blot analysis for GPX4 protein

Western blotting was performed according to the literature description for GPX4 (19 kDa) detection. GAPDH (36 kDa) was used as the loading control. 4T1 cells were seed into 24-well plates at a density of 1×10^5 cells in RPMI-1640 medium at 37 °C for 24 h under hypoxia followed by the treatment with Ir-2 complexes (2 μM, at 37 °C) in the dark for 4 h incubation. The supernatant was replaced with a fresh culture medium and cells were subjected to

irradiation (405 nm light, 20 mW/cm^2, 10 min). Cells without irradiation were replaced with a fresh culture medium and maintained in the dark. After 0.5 h of the additional incubation, 4T1 cells were collected and washed twice with PBS. All cells were collected. The expression of GPX4 in 4T1 cells upon formulation treatment was analyzed by western blotting according to the protocol method.

3. In vivo antitumor experiments

Female BALB/c mice with 5-6 weeks of age, were purchased from Hunan SJA Laboratory Animal Co., Ltd (Hunan, China). All animal operations were in conformity to Guidelines for Care and Use of Laboratory Animals of South China University of Technology (SCUT) and approved by animal ethics of SCUT. Female B/BLAC mice (18 ± 2 g, 5-6 weeks old) 4T1 cells (1×10^7) were injected into the right mammary fat pads to establish an orthotopic 4T1 tumour model. After the tumor volumes reached to 50-80 mm^3, the mice were used for subsequent experiments. All animal experiments were approved by the ethics committee of the South China University of Technology.

To examine in vivo tumour growth inhibition by Ir2, we randomly divided the 4T1-tumor-bearing B/BLAC female mice into 4 groups (n = 5 in per group): (i) control group (with only PBS injection), (ii) only light irradiation group (PBS injection, 405 nm, 20 mW/cm^2, 10 min), (iii) only Ir2 group (Intratumoral injection, 25 μL, 500 μM), and iv) Ir2+Light irradiation group (Intratumoral injection, 25 μL, 500 μM, 405 nm, 20 mW/cm^2, 10 min). Tumour volume and body weight were recorded every 2 days for 16 days to note the therapeutic effect. At the end of experiment, the mice in different groups were sacrificed and their tumours were collected for photographing and weighing. After treating the tumours with different treatments, they were stained with hematoxylin and eosin (H&E), terminal deoxynucleotidyl transferase-mediated dUTP nick-end labeling (TUNEL) on the next day and then examined by fluorescence microscope.

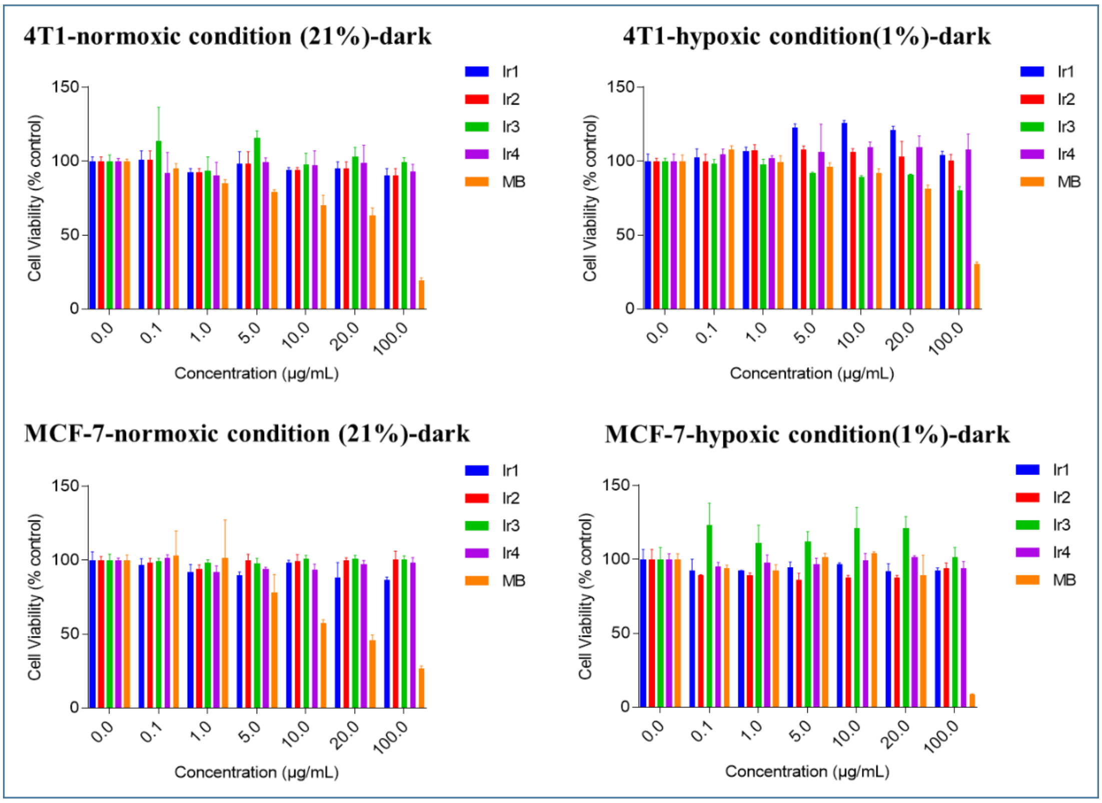

Figure S29. The MTT assay results of the dark toxicity experiment of 4T1 and MCF-7 under hypoxia (1% O_2) and normoxia (21% O_2).

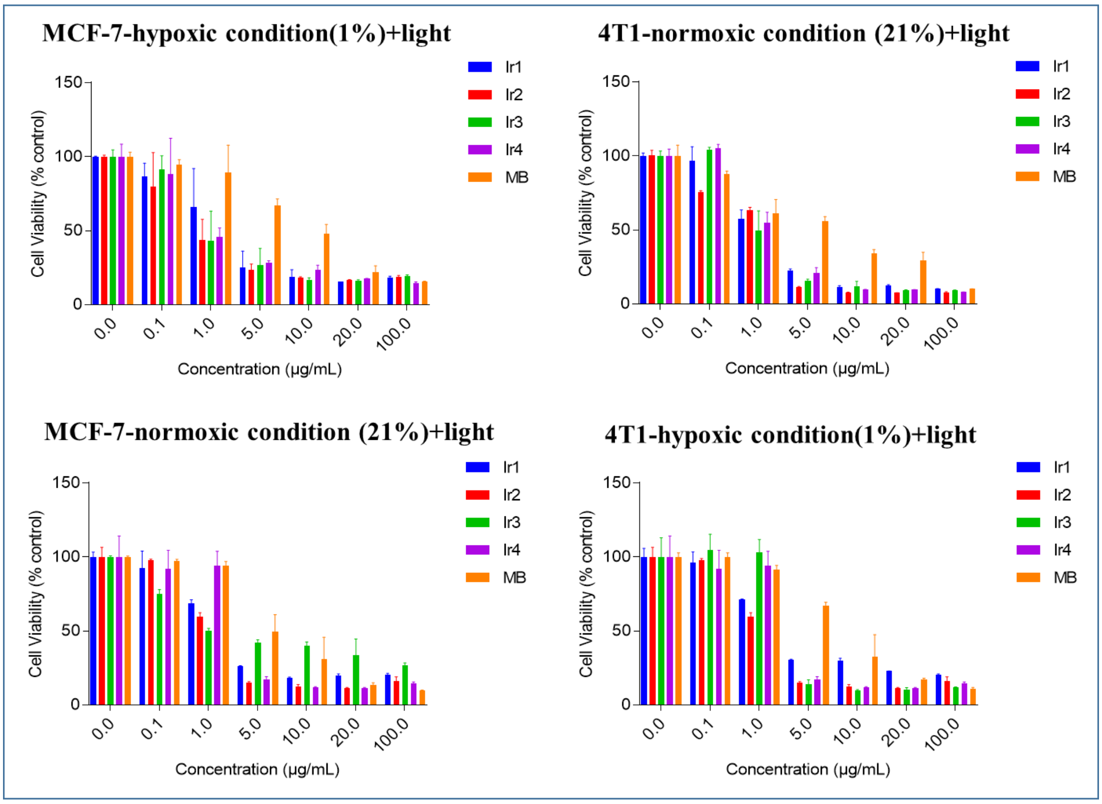

Figure S30. The MTT assay results of the phototoxicity experiment of 4T1 and MCF-7 under hypoxia (1% O_2) and normoxia (21% O_2).

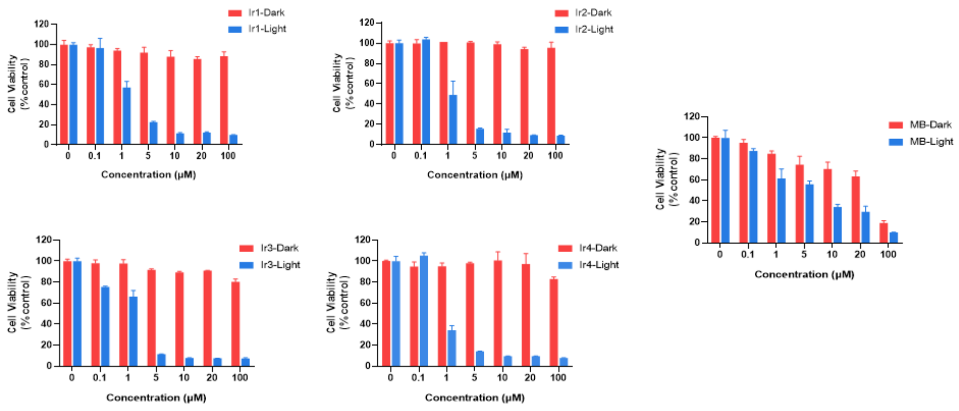

Compound	Dark (μM)	Light(μM)	PI
Ir1	>100	0. 91	>109. 89
Ir2	>100	0.57	>175.44
Ir3	>100	1. 83	>54.64
Ir4	>100	0.76	>131.48
MB	48. 18	6.91	6.90

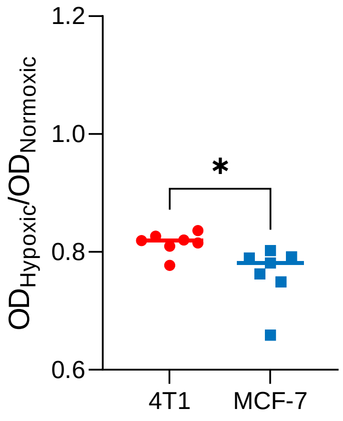

Figure 31. (a) The shows the results of the phototoxicity experiment of 4T1 at low concentrations that were re-examined. It can be concluded that Ir2 is the most toxic. As for the high phototoxicity index of Ir4, I guess it is related to its targeting to mitochondria. Because Ir2 is the most toxic, follow-up experiments are done with Ir2. (b) Cell viability experiments using CCK8 reagent under different oxygen concentrations for 4T1 and MCF-7, and the results showed that, compared to 4T1, MCF-7 is more sensitive to low oxygen environments. Under low oxygen conditions, the proliferation and survival rate of MCF-7 cells decreased significantly, while the proliferation and survival rate of 4T1 cells remained relatively stable. This may indicate that our material is more toxic in hypoxic environments.

4. Computational studies

4.1. Density Functional Theory (DFT) and time-dependent DFT (TDDFT) calculations:

The ground-state (S_0) structures of four Ir-complexes, designated as Ir1, Ir2, Ir3, and Ir4, have been optimized using density functional theory (DFT) calculations using the B3LYP[7-10] functional and the def2-SVP[11] basis set. We also included dispersion corrections using the D3BJ[12-13] method and the RIJCOSX[14] approximation to improve the accuracy of our calculations. All calculations were performed using the ORCA program package (version 5.0.3)[15] running on a high-performance computing cluster. On the basis of optimized geometries of the S_0 state, time-dependent TDDFT[16-18] calculations using the CAM-B3LYP[19] functional and the same basis sets were carried out to obtain various the excited states. To accomplish this, we performed TDDFT calculations on both the singlet (S_1) and triplet (T_1) excited states of the optimized geometries obtained from the CAM-B3LYP/def2-SVP calculations. The first three singlet and triplet excited states were calculated and optimized for each complex.

To gain insight into the Charge Density Difference (CDD) for the optimized geometries of the Ir-complexes, we performed analysis using Multiwfn program.[20] Furthermore, the CDD analysis was performed to visualize the changes in charge density that occur during excitation. The CDD maps were generated using the wave function files of the optimized geometries for each complex. To gain insight into the spatial and energetic characteristics of the system, we conducted a natural transition orbital (NTO) analysis specifically in the triplet state. To create the images of the optimized geometries and their respective CDD maps, the Jmol software was used.

The optimized structure of ground-state (S_0), first singlet (S_1) and triplet excited state (T_1) of the Ir-complexes are shown below in Figure S32-34. The hydrogen bonds are intentionally omitted in the figure to enhance clarity and facilitate a focused visual representation of the molecular structure. The determined dihedral angle (θ(CCCN)) between the proton donating and proton accepting groups.

Abbreviations: When representing photocatalysts Ir1, Ir2, Ir3, and Ir4, the total charge and spin state are denoted as follows, since the ground states are cationic (excluding the hexafluorophosphate counterion): ^{1}[Ir]–lowest energy singlet state of Ir-complexes, ^{3}[Ir]–lowest energy triplet state of Ir-complexes.

4.2. Optimized Structures

4.2a. Ground-state Characterization

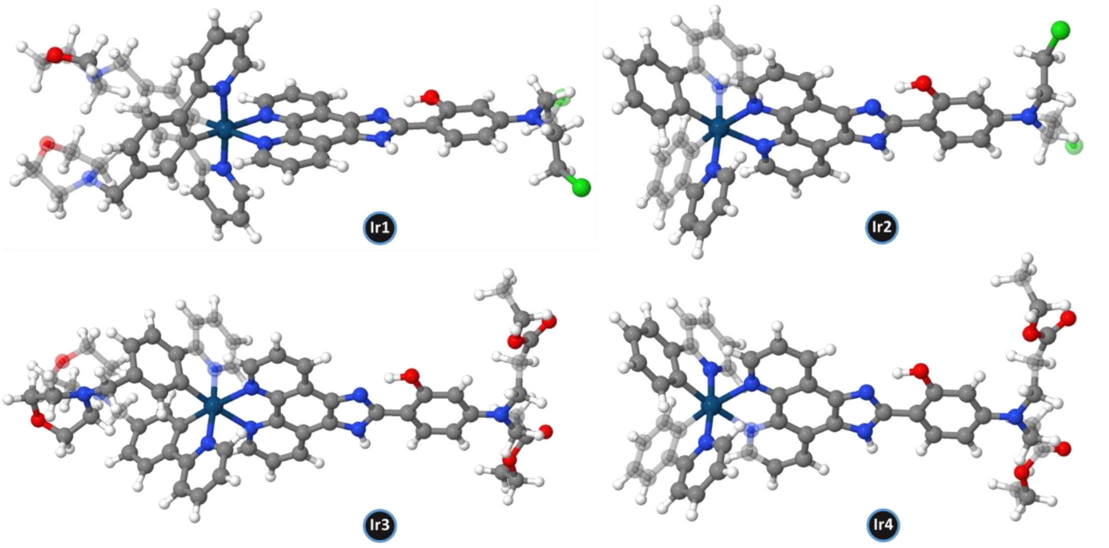

Figure S32. Views of the optimized ground-state (S_0) structure of Ir1−Ir4 at B3LYP/6-31G(d) level.

4.2b. Excited state Characterization

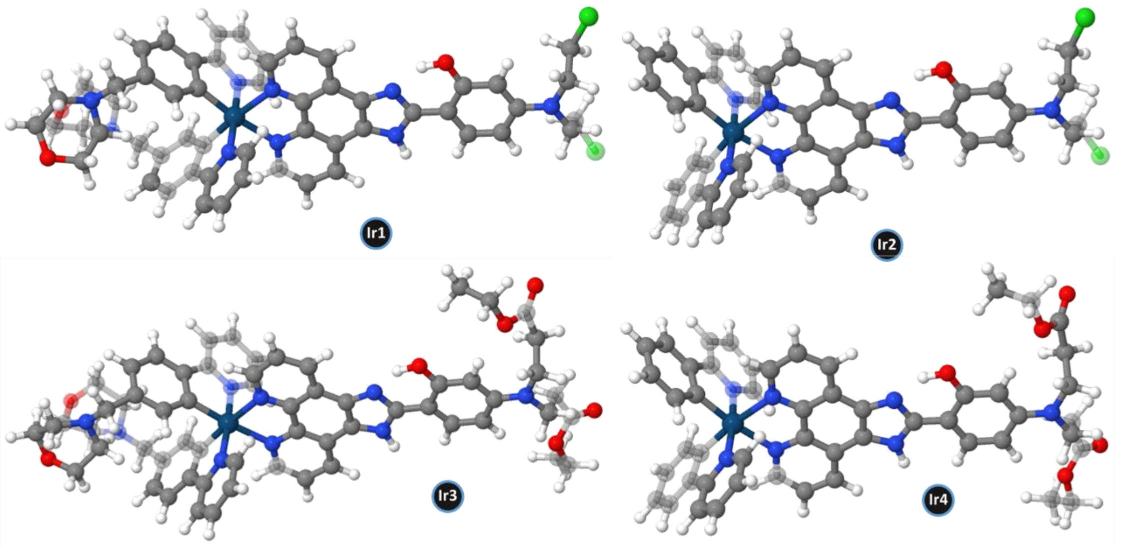

Figure S33. Optimized first singlet excited state (S_1) geometries of ^{1}[Ir]-complexes at the CAM-B3LYP/def2-SVP level of theory.

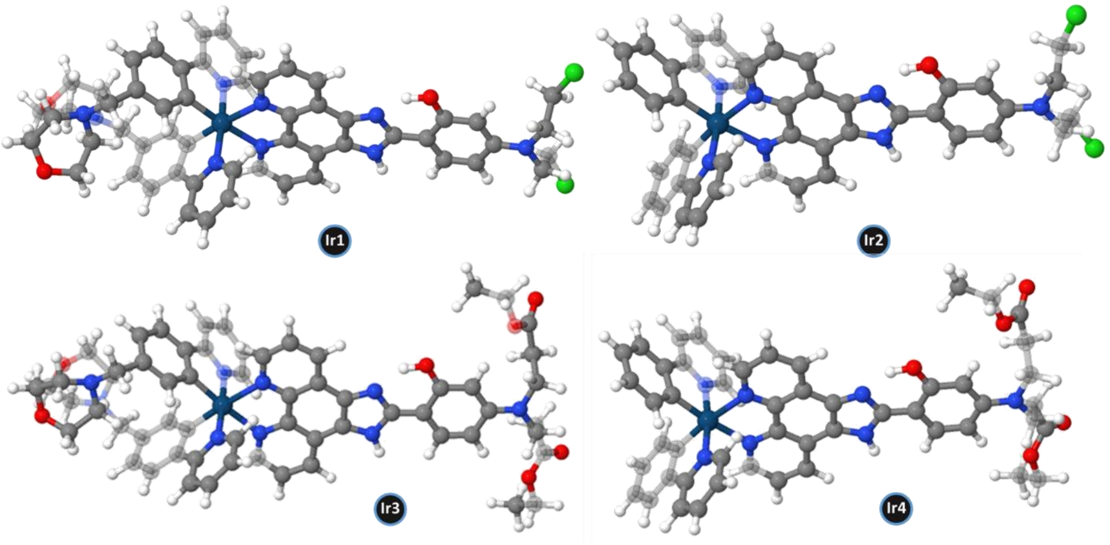

Figure S34. Optimized first triplet excited state (T_1) geometries of ^{3}[Ir]-complexes at the CAM-B3LYP/def2-SVP level of theory.

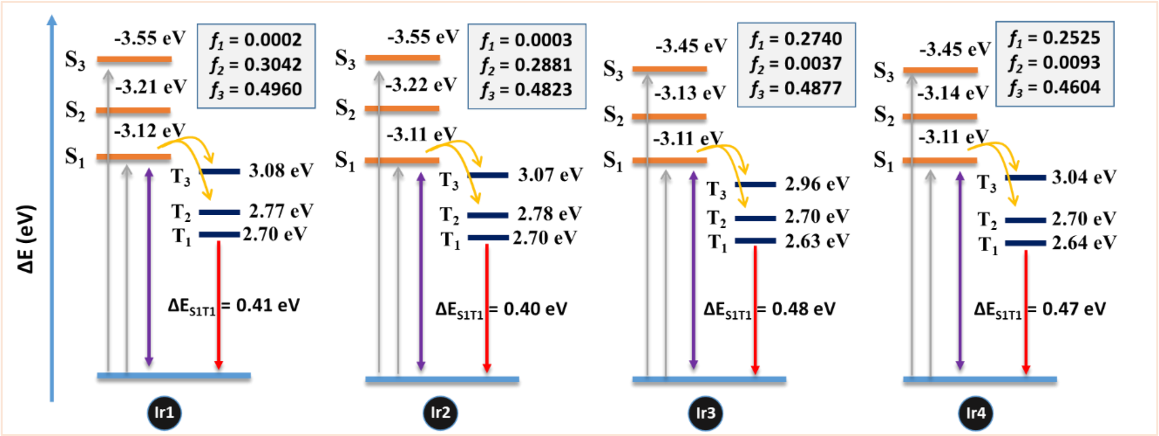

Figure S35. Schematic diagram for electronic transitions of Ir1-Ir4 on a basis of TD-DFT calculation at the CAM-B3LYP/def2-SVP level of theory. Vertical transition energy (ΔE, eV) and the ΔE_{S1T1} between singlet and triplet states are also presented in the figures.

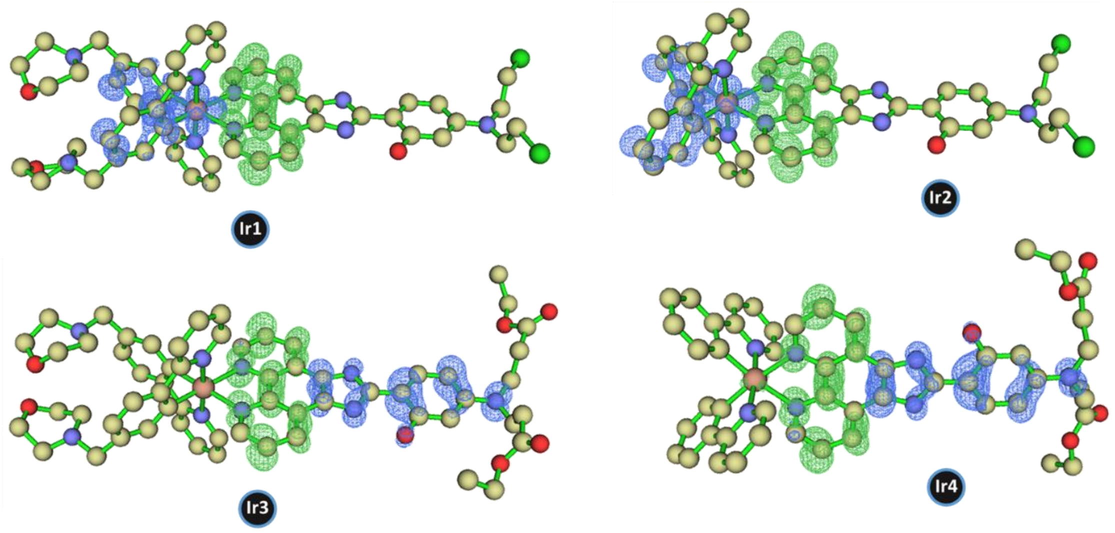

Figure S36. Charge density difference (CDD) of ^{3}[Ir]-complexes in the enol form using the CAM-B3LYP functional. The CDD plot shows the change in the charge distribution induced by the MLCT or LLCT transition of the enol form. In this map, the isovalue is set to 0.002. The green and blue densities correspond to increase ($\boldsymbol{\delta}^{-}$) and decrease ($\boldsymbol{\delta}^{+}$) of the excited state density with respect to the ground state negative charge densities, respectively.

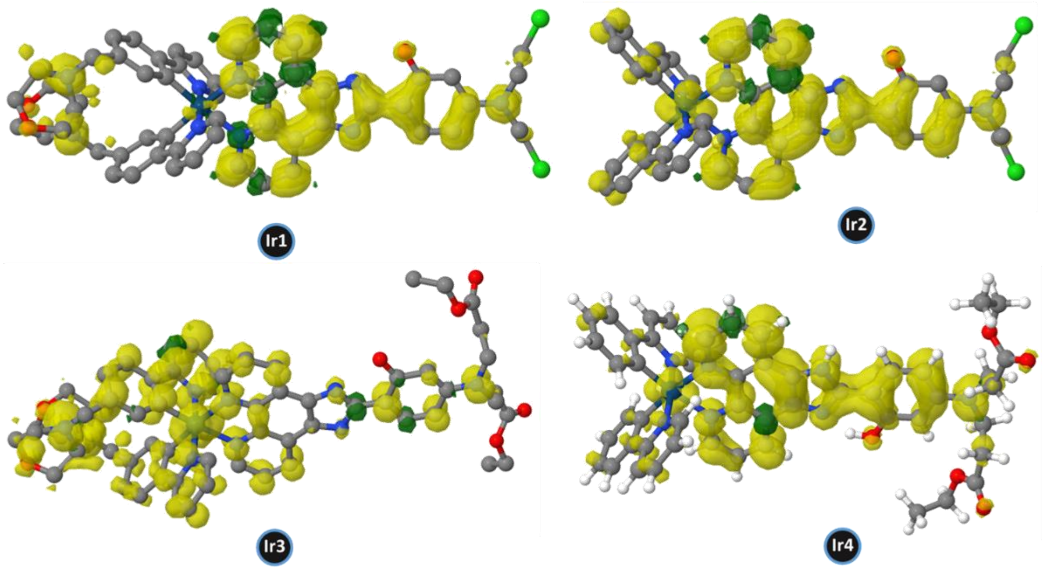

Figure S37. Spin density plots of ^{3}[Ir]-complexes in the enol form using the CAM-B3LYP functional.

4.3. Natural transition orbitals

NTOs are obtained by diagonalizing the difference density matrix between the excited and ground states, and they represent the orbitals that are predominantly involved in the electronic transition. The occupation number for each NTO gives an indication of the electron density that is transferred between the initial and final states.

Complex	HONTO	LUNTO
**Ir1**	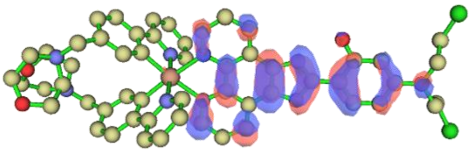 **HONTO 260**	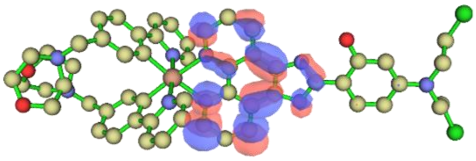 **LUNTO 261**
**Ir2**	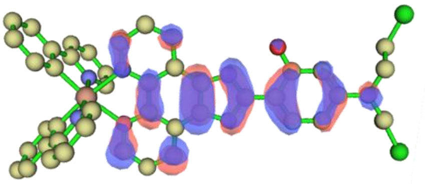 **HONTO 206**	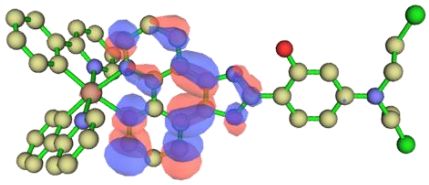 **LUNTO 207**
**Ir3**	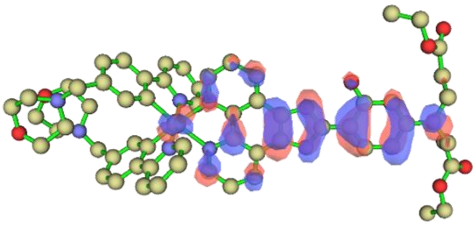 **HONTO 282**	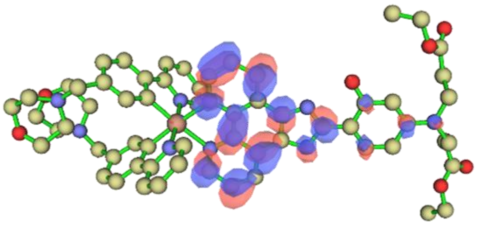 **LUNTO 283**
**Ir4**	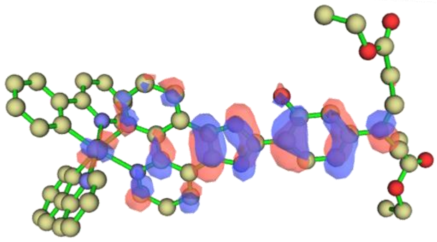 **HONTO 228**	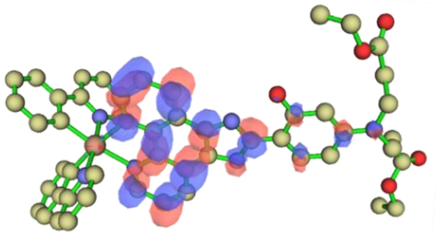 **LUNTO 229**

Figure S38. The highest occupied natural transition orbital (HONTO) and lowest unoccupied natural transition orbital (LUNTO) of the optimized structure of the T1 state of the enol form of Ir1-Ir4 complexes. The contributions of HONTO and LUNTO are more than 50 %.

References:

1. Féau, C.; Klein, E.; Dosche, C.; Kerth, P.; Lebeau, L. *Org. Biomol. Chem.* **2009**, *7*, 5259−5270.

2. (a) Lachaud, F.; Quaranta, A.; Pellegrin, Y.; Dorlet, P.; Charlot, M.-F.; Un, S.; Leibl, W.; Aukauloo, A. *Angew. Chem., Int. Ed.* **2005**, *44*, 1536 –1540. (b) Manbeck, G. F.; Fujita, E.; Concepcion, J. J. *J. Am. Chem. Soc.* **2016**, *138*, 11536−11549.

3. Zhang, K. Y.; Gao, P.; Sun, G.; Zhang, T.; Li, X.; Liu, S.; Zhao, Q.; Lo, K. K.-W.; Huang, W. *J. Am. Chem. Soc.* **2018**, *140*, 7827−7834.

4. Mandal, S.; Ghatak, C.; Rao, V. G.; Ghosh, S.; Sarkar. N. *J. Phys. Chem. C* **2012**, *116*, 5585−5597.

5. Kim, S.; Choi, J.; Cho, D. W.; Ahn, M.; Eom, S.; Kim, J.; Wee, K.-R.; Ihee, H.; *Chem. Sci.* **2022**, *13*, 3809−3818.

6. Sheik-Bahae, M.; Said, A. A.; Wei, T.-H.; Hagan, D. J.; Van Stryland, E. W, Sensitive Measurement of Optical Nonlinearities Using a Single Beam. *IEEE J. Quantum Electron.* 1990, 26, 760–769.

7. Becke, A. D. Becke's three parameter hybrid method using the LYP correlation functional. *J. Chem. Phys.* **1993**, *98*, 5648−5652.

8. Lee, C.; Yang, W.; Parr, R. G. Development of the Colle-Salvetti Correlation-Energy Formula into a Functional of the Electron Density. *Phys. Rev. B: Condens. Matter Mater. Phys.* **1988**, *37*, 785−789.

9. Vosko, S. H.; Wilk, L.; Nusair, M. Accurate Spin-Dependent Electron Liquid Correlation Energies for Local Spin Density Calculations: a Critical Analysis. *Can. J. Phys.* **1980**, *58*, 1200−1211.

10. Stephens, P. J.; Devlin, F. J.; Chabalowski, C. F.; Frisch, M. J. Ab Initio Calculation of Vibrational Absorption and Circular Dichroism Spectra Using Density Functional Force Fields. *J. Phys. Chem.* **1994**, *98*, 11623−11627.

11. Schäfer, A.; Horn, H.; Ahlrichs, R. Fully Optimized Contracted Gaussian Basis Sets for Atoms Li to Kr. *J. Chem. Phys.* **1992**, *97*, 2571−2577.

12. Grimme, S.; Antony, J.; Ehrlich, S.; Krieg, H. A Consistent and Accurate Ab Initio Parametrization of Density Functional Dispersion Correction (DFT-D) for the 94 Elements H-Pu. *J. Chem. Phys*. **2010**, *132*, 154104

13. Grimme, S.; Ehrlich, S.; Goerigk, L. Effect of the Damping Function in Dispersion Corrected Density Functional Theory. *J. Comput. Chem*. **2011**, *32*, 1456−1465.

14. Izsá k, R.; Neese, F. Speeding up Spin-Component-Scaled Third-Order Pertubation Theory with the Chain of Spheres Approximation: The COSX-SCS-MP3 Method. *Mol. Phys*. **2013**, *111*, 1190−1195.

15. Neese, F. The ORCA Program System. *Wiley Interdiscip. Rev.: Comput. Mol. Sci*. **2012**, *2*, 73−78.

16. Casida, M. E.; Huix-Rotllant, M. Progress in Time-Dependent Density-Functional Theory. *Annu. Rev. Phys. Chem*. **2012**, *63*, 287−323.

17. Marques, M. A. L.; Gross, E. K. U. Time-dependent density functional theory. *Annu. Rev. Phys. Chem*. **2004**, *55*, 427−455.

18. Dreuw, A.; Head-Gordon, M. Single-reference ab initio methods for the calculation of excited states of large molecules. *Chem. Rev*. **2005**, *105*, 4009−4037.

19. Yanai, T.; Tew, D. P.; Handy, N. C. A New Hybrid Exchange-Correlation Functional Using the Coulomb-Attenuating Method (CAM-B3LYP). *Chem. Phys. Lett*. **2004**, *393,* 51–57.

20. Lu, T.; Chen, F. Multiwfn: A Multifunctional Wavefunction Analyzer. *J. Comput. Chem*. **2012**, *33*, 580–592.

5. Characterization of compounds

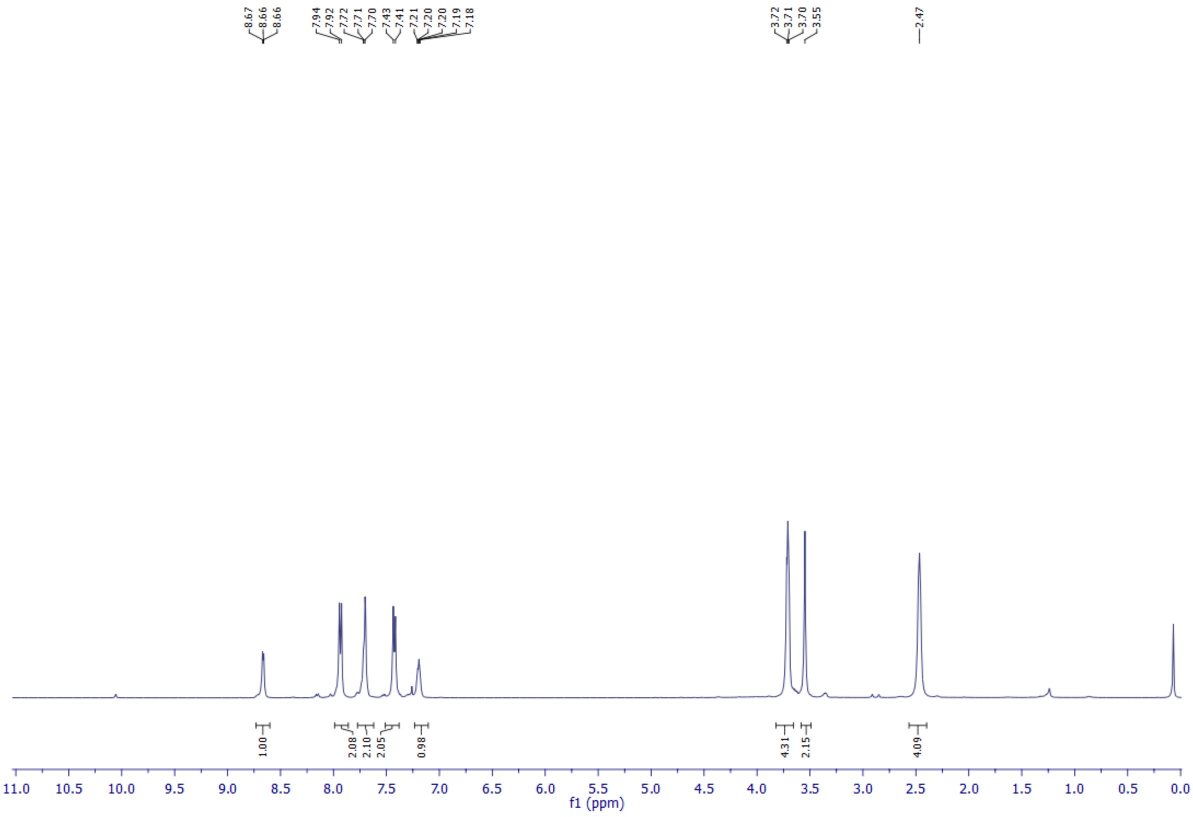

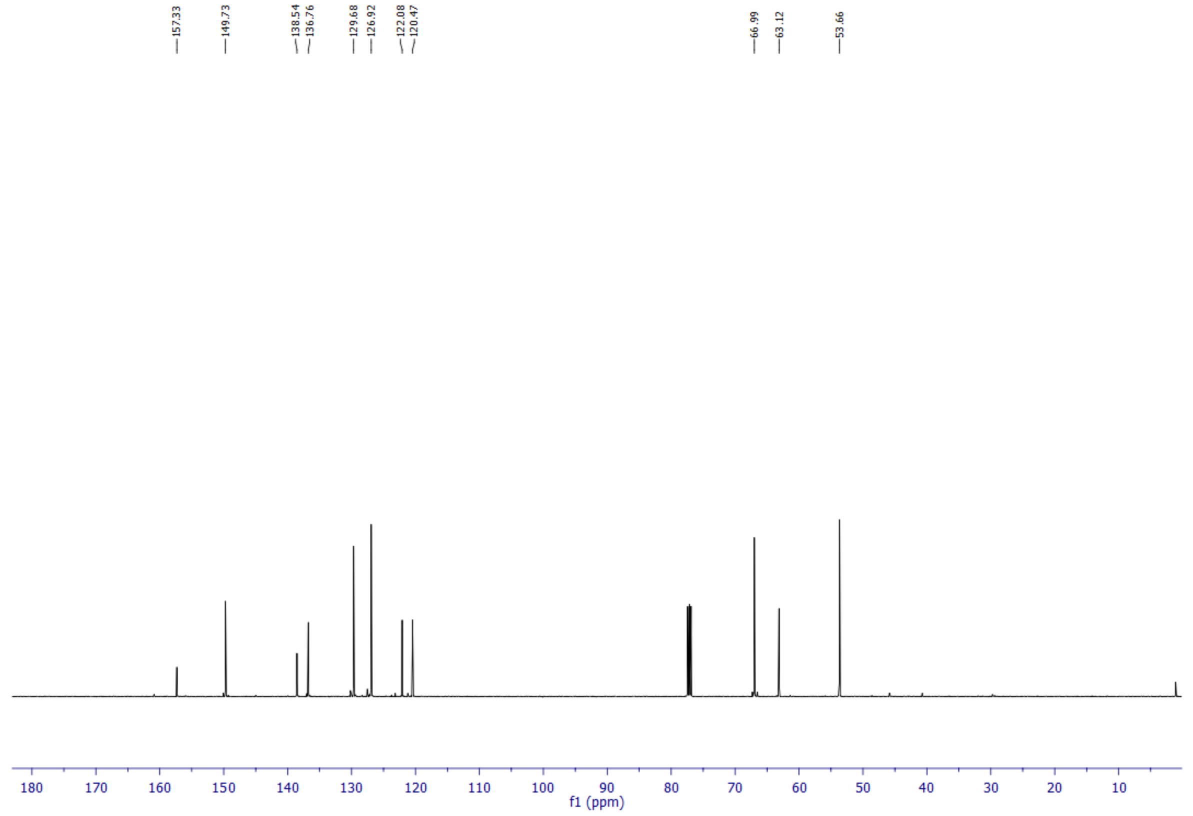

Figure S39. ^{1}H and ^{13}C NMR spectra of compound, 4-(4-(pyridin-2-yl)benzyl)morpholine.

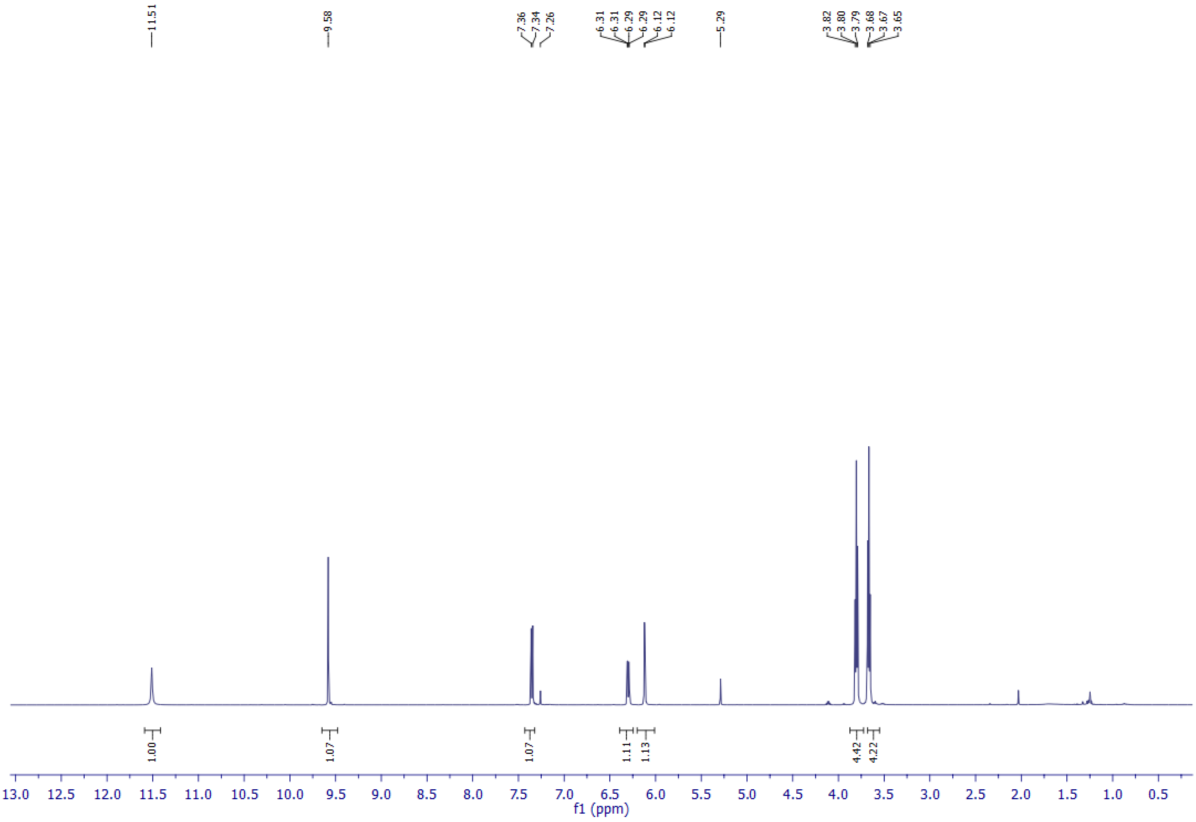

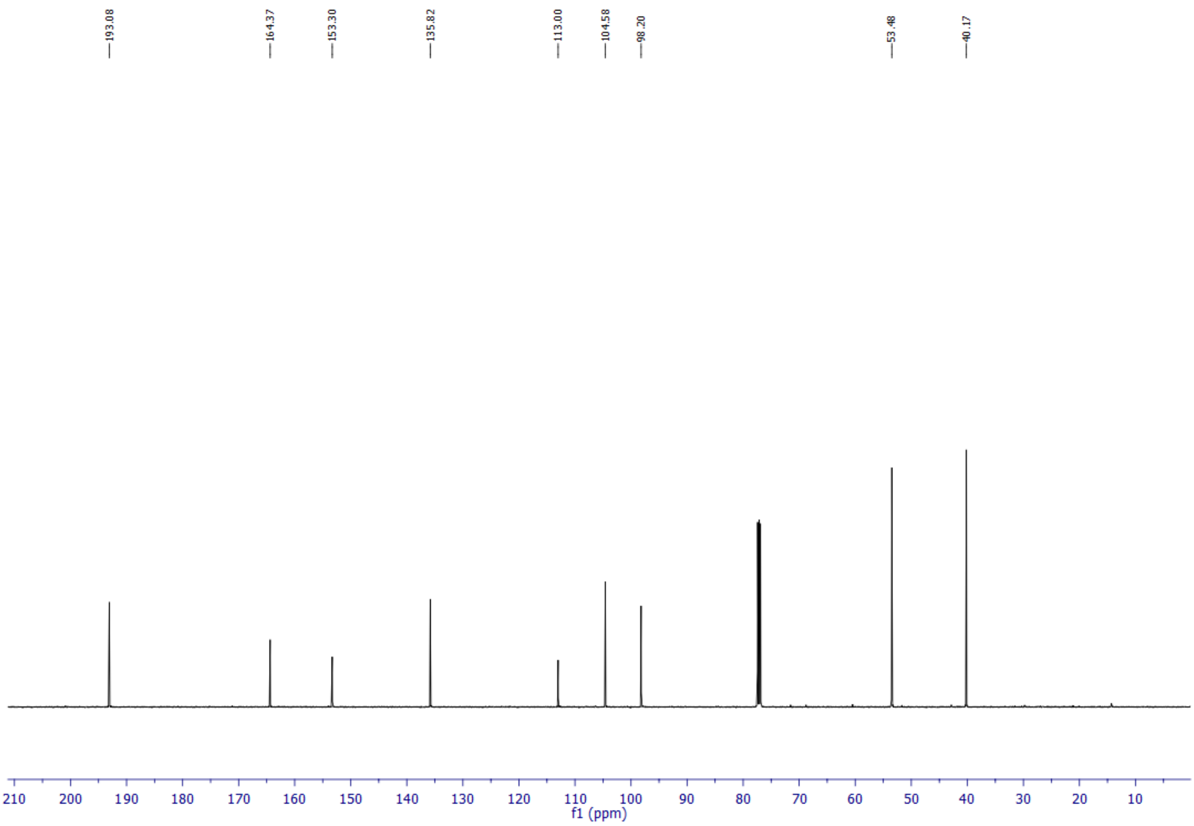

Figure S40. ^{1}H and ^{13}C NMR spectra of compound, 4-(Bis(2-chloroethyl)amino)-2-hydroxybenzaldehyde.

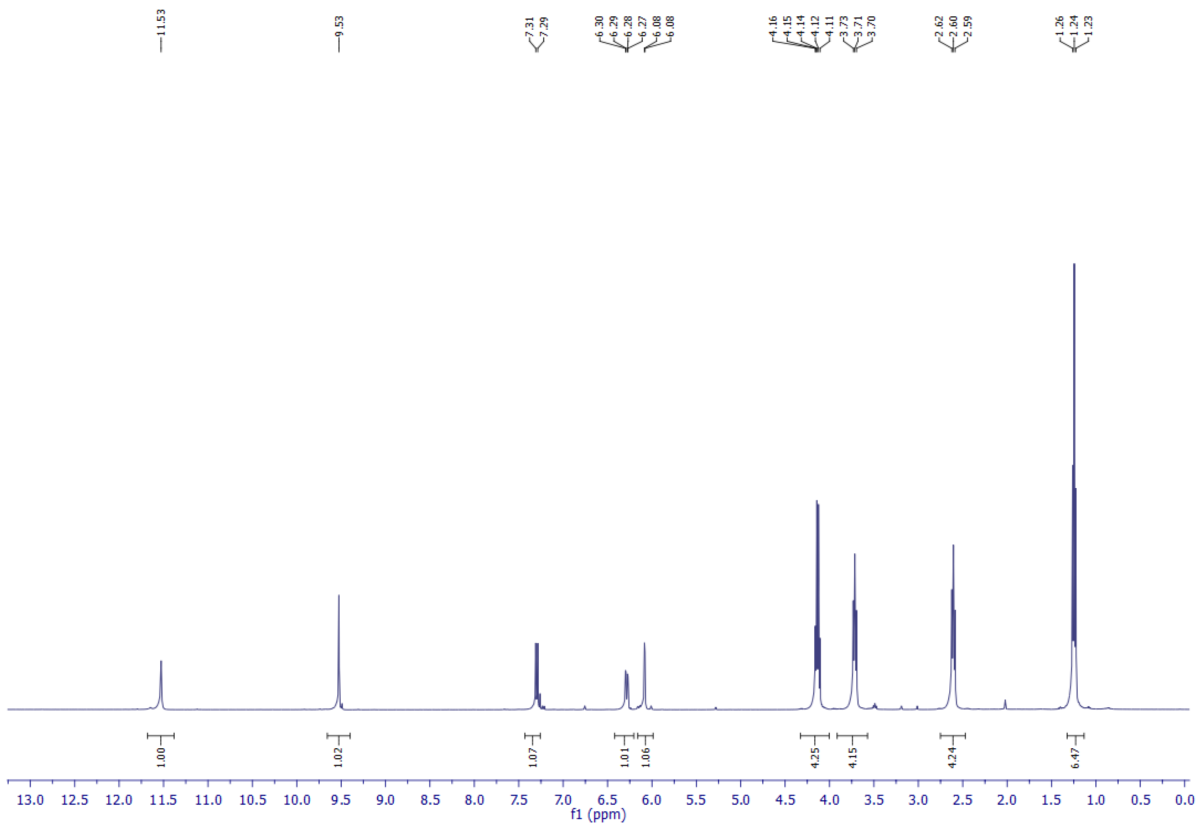

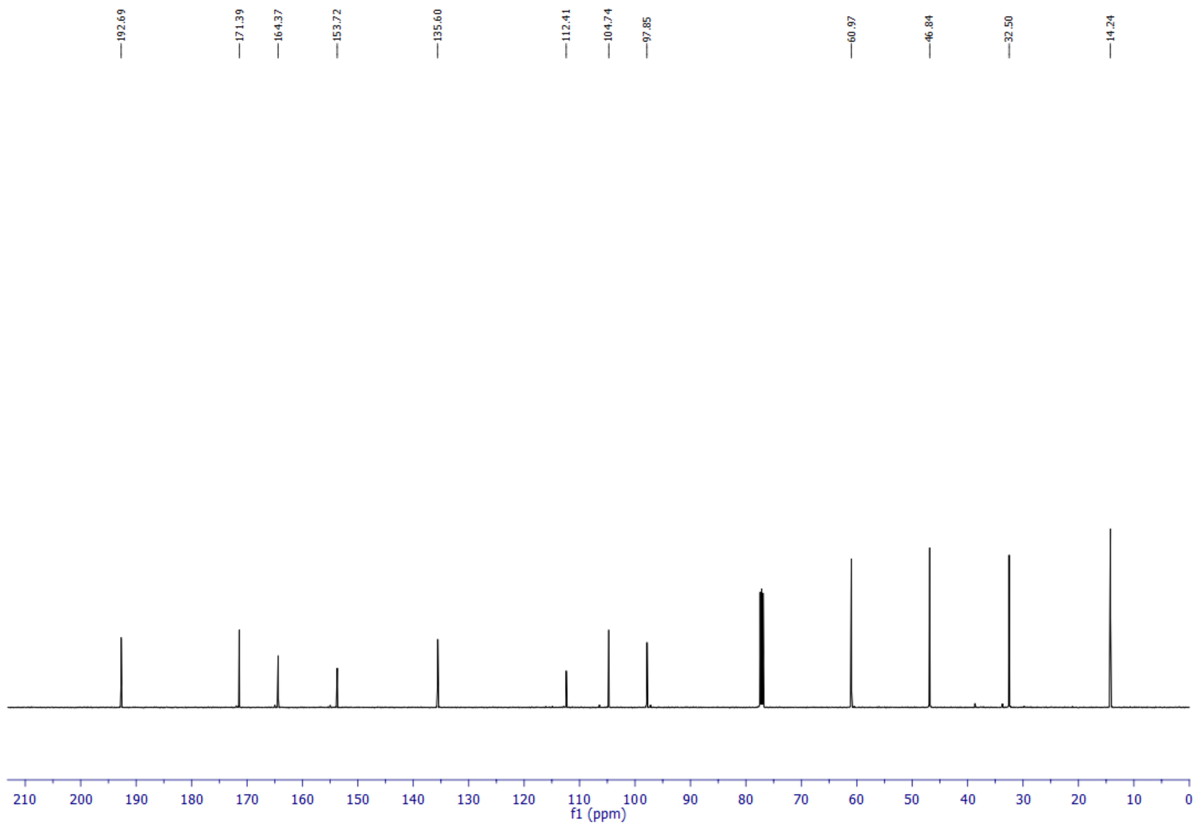

Figure S41. ^{1}H and ^{13}C NMR spectra of compound, Diethyl 3,3'-((4-formyl-3-hydroxyphenyl)azanediyl)dipropionate.

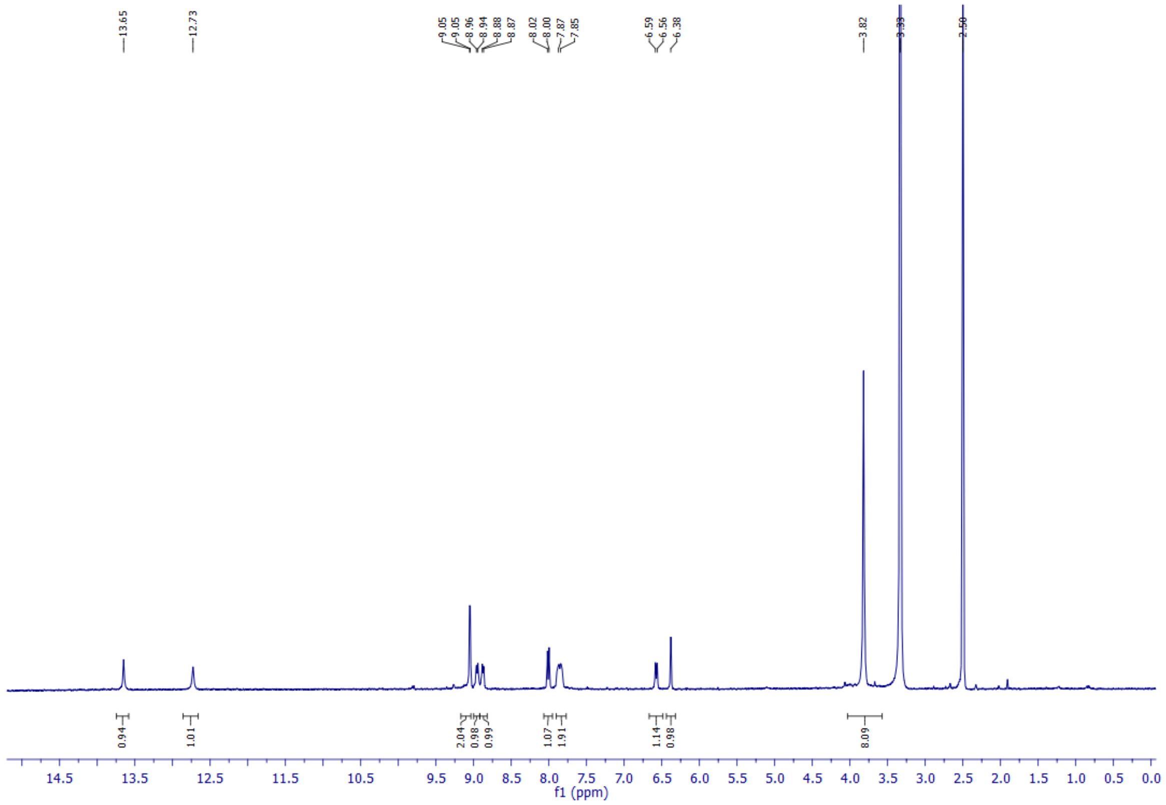

Ligand 1

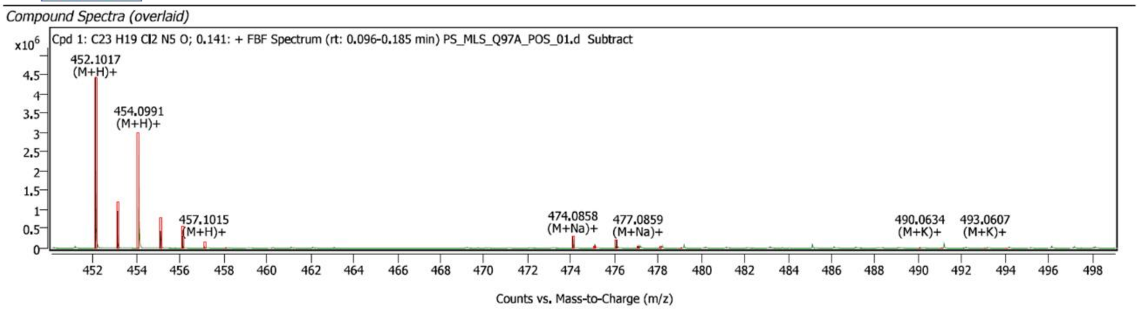

Compound ID Table

Name	Formula	Species	RT	RT Diff	Mass	CAS	ID Source	Score	Score (Lib)	Score (Tgt)
	C23 H19 Cl2 N5 O	(M+H)+<br>(M+Na)+<br>(M+K)+	0.141		451.0948		FBF	99.41		99.41

Figure S42. ^{1}H NMR and ESI HRMS spectra of compound, 5-(bis(2-chloroethyl)amino)-2-(1*H*-imidazo[4,5-*f*][1,10]phenanthrolin-2-yl)phenol.

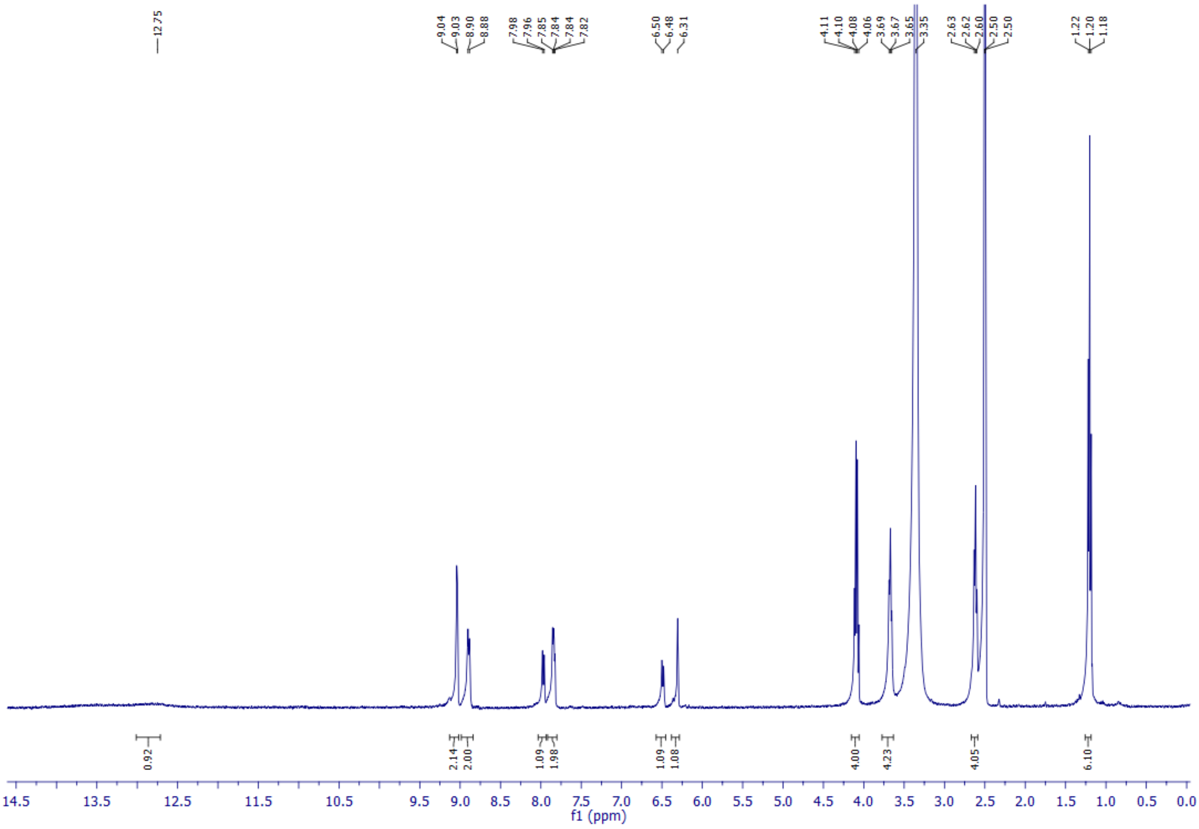

Ligand 2

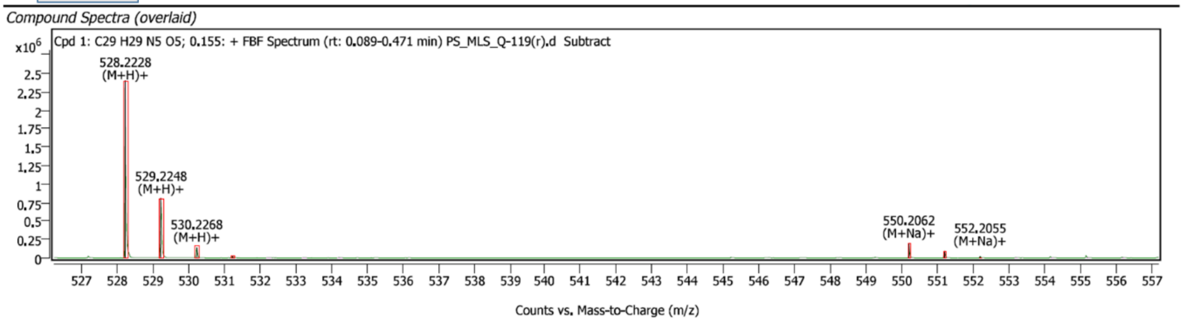

Compound ID Table

Name	Formula	Species	RT	RT Diff	Mass	CAS	ID Source	Score	Score (Lib)	Score (Tgt)
	C29 H29 N5 O5	(M+H)+ (M+Na)+	0.155		527.2152		FBF	93.36		93.36

Figure S43. ^{1}H NMR and ESI HRMS spectra of compound, (diethyl 3,3'-((3-hydroxy-4-(1H-imidazo[4,5-*f*][1,10]phenanthrolin-2-yl)phenyl)azanediyl)dipropionate).

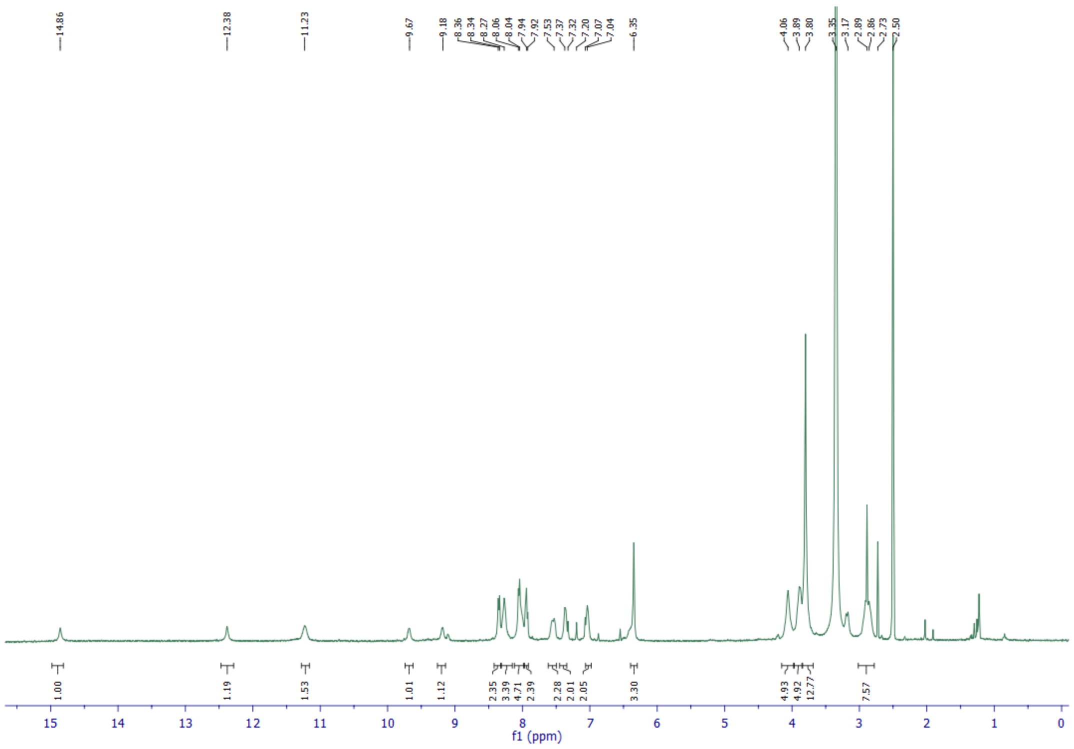

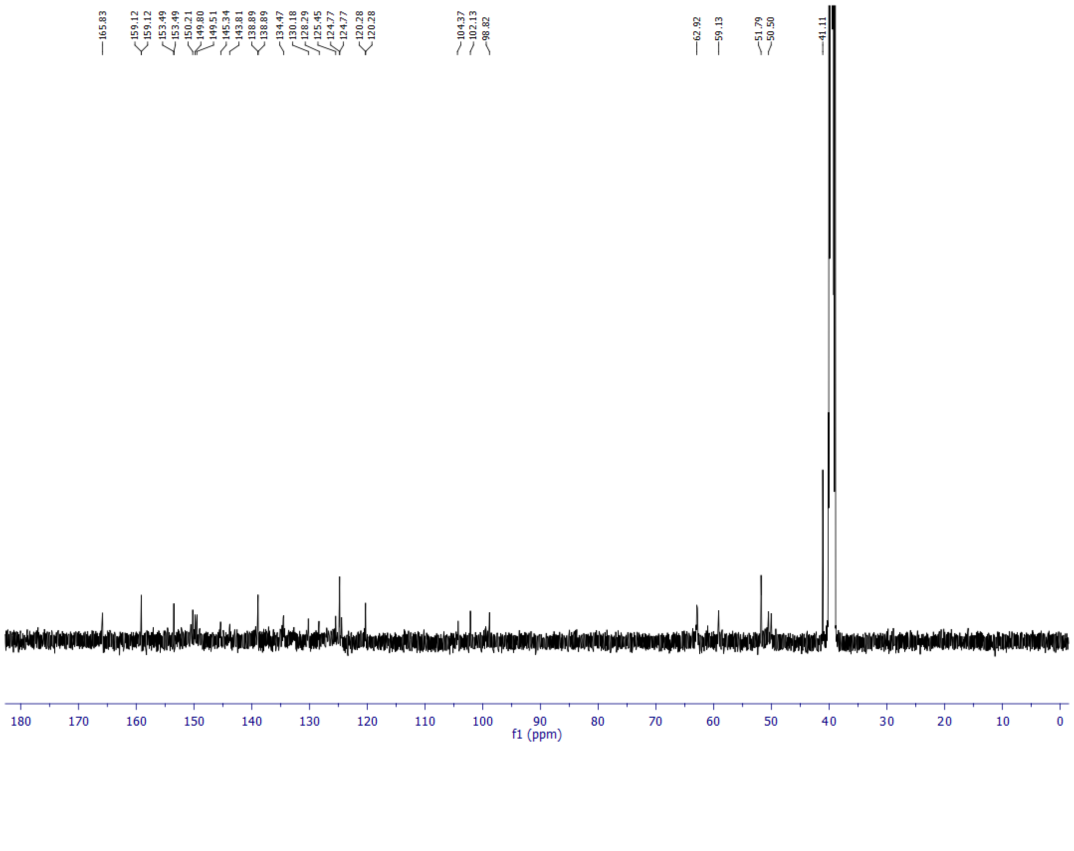

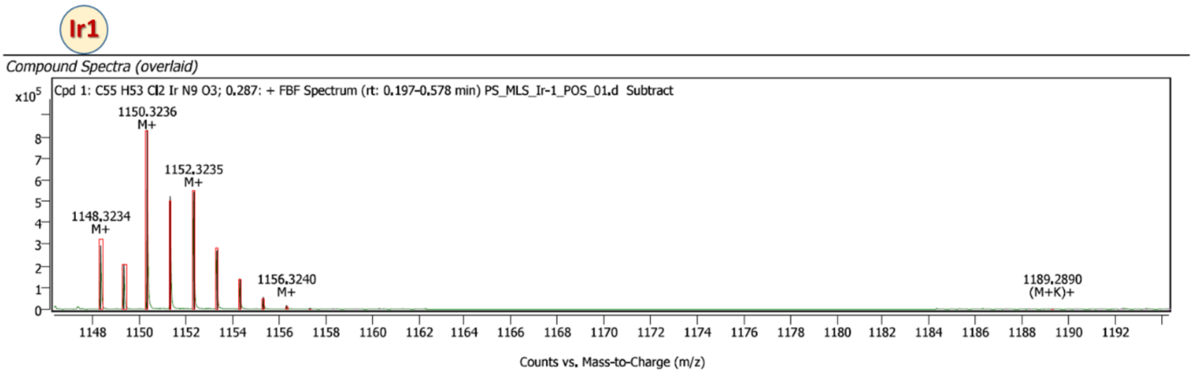

Compound ID Table

Name	Formula	Species	RT	RT Diff	Mass	CAS	ID Source	Score	Score (Lib)	Score (Tgt)
	C55 H53 Cl2 Ir N9 O3	M+ (M+K)+	0,287		1148,3226		FBF	92,95		92,95

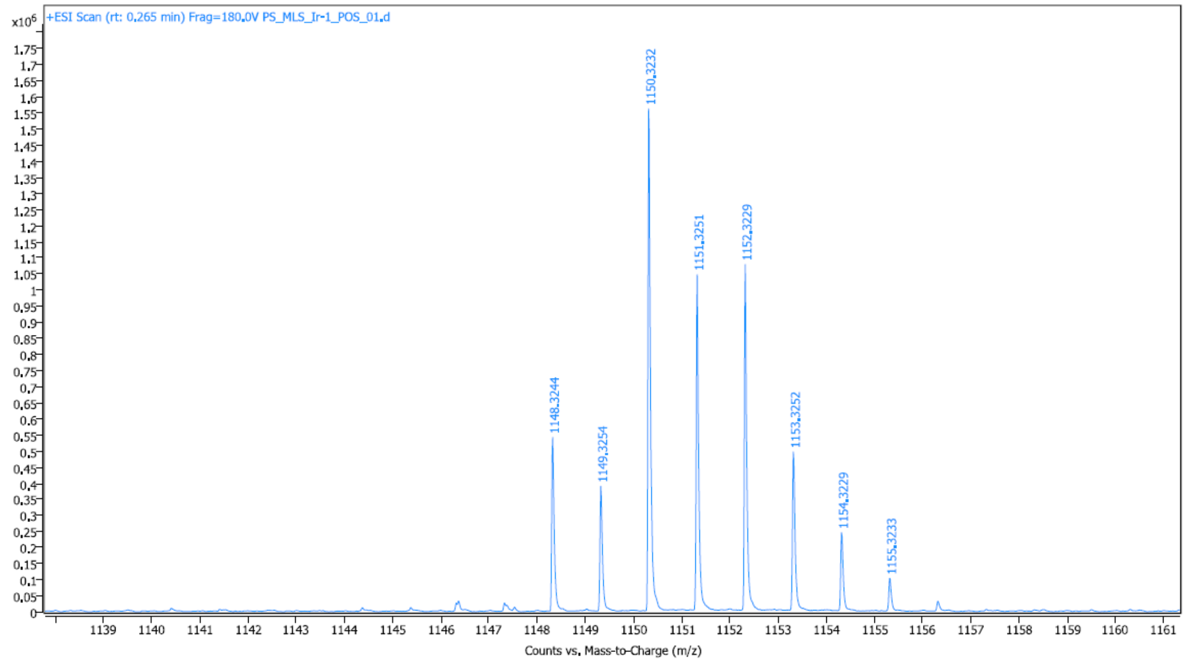

Figure S44. ^{1}H, ^{13}C NMR and ESI HRMS spectra of Ir-1 complex.

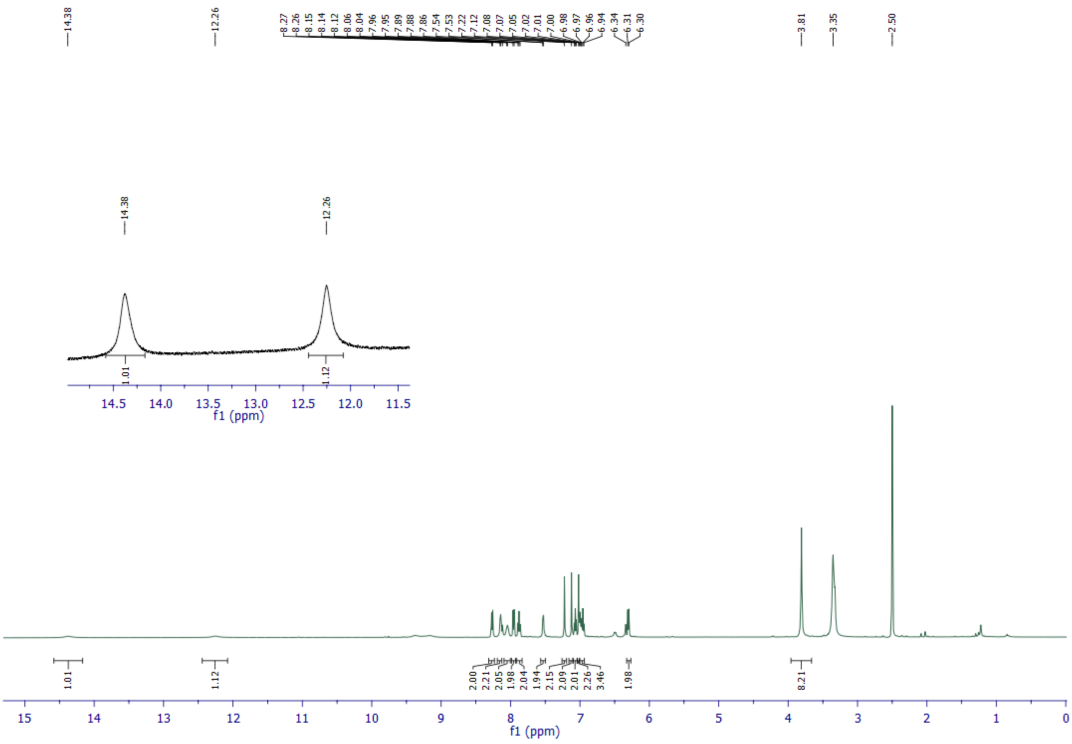
14.38
12.26
8.27 8.26 8.15 8.14 8.12 8.06 8.04 7.96 7.95 7.89 7.88 7.86 7.54 7.53 7.22 7.12 7.08 7.07 7.05 7.02 7.01 7.00 6.98 6.97 6.96 6.94 6.34 6.31 6.30
3.81
3.35
2.50
1.01
1.12
2.00 2.21 2.05 1.98 2.04 1.94 2.15 2.09 2.01 2.26 3.46
1.98
8.21
f1 (ppm)

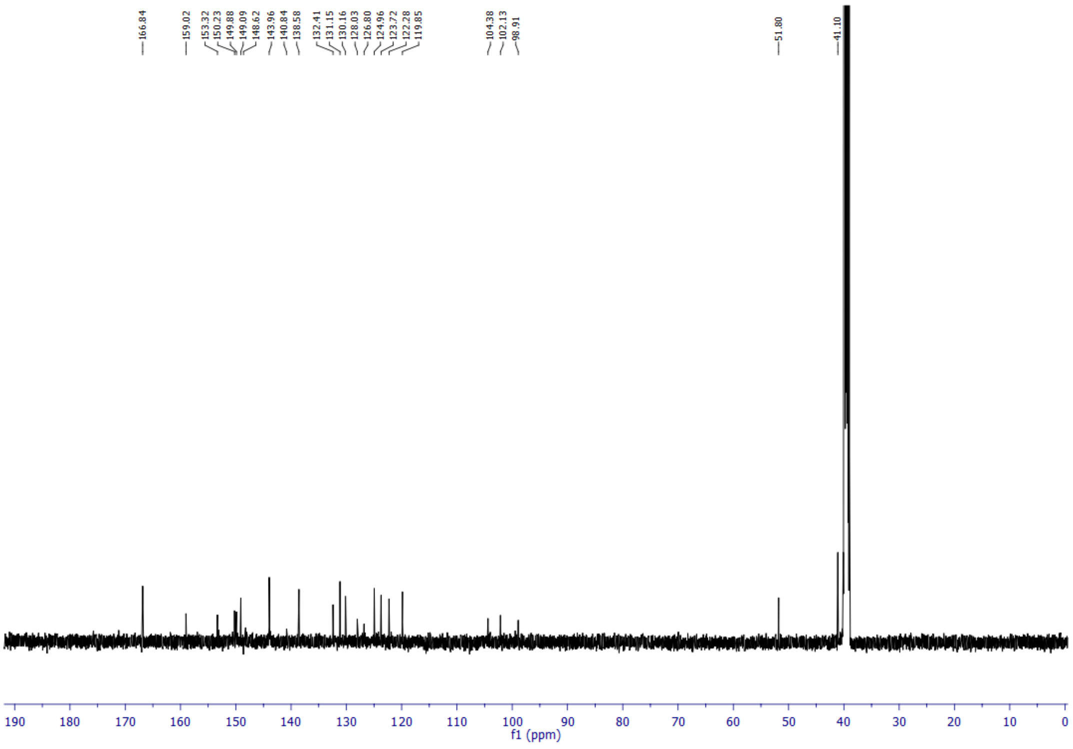
166.84
159.02 153.32 150.23 149.88 149.09 148.62 143.96 140.84 138.58 132.41 131.15 130.16 128.03 126.80 124.96 123.72 122.28 119.85
104.38 102.13 98.91
51.80
41.10
f1 (ppm)

Ir2

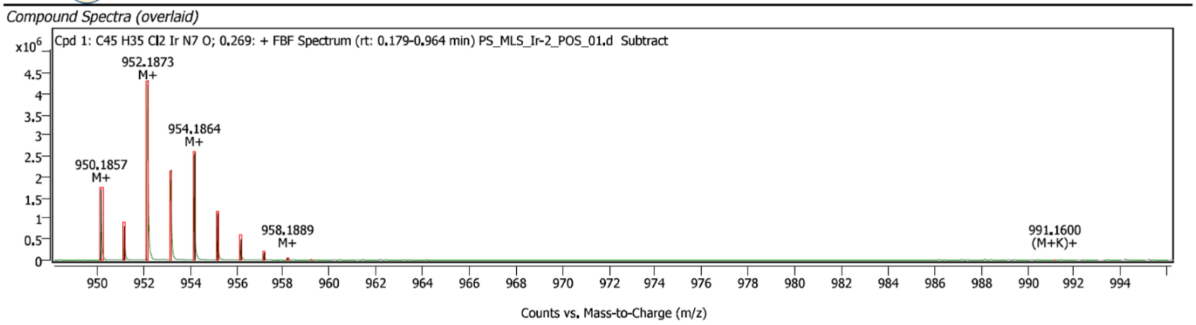

Compound ID Table

Name	Formula	Species	RT	RT Diff	Mass	CAS	ID Source	Score	Score (Lib)	Score (Tgt)
	C45 H35 Cl2 Ir N7 O	M+ (M+K)+	0.269		950.1865		FBF	95.52		95.52

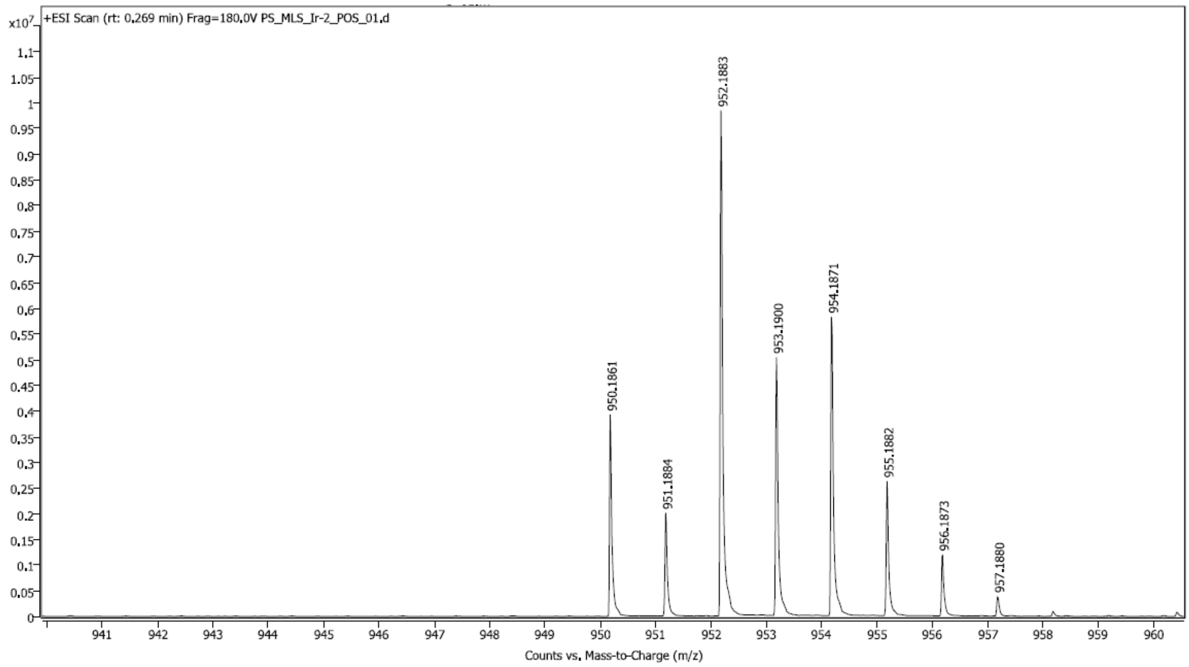

Figure S45. ^{1}H, ^{13}C NMR and ESI HRMS spectra of Ir-2 complex.

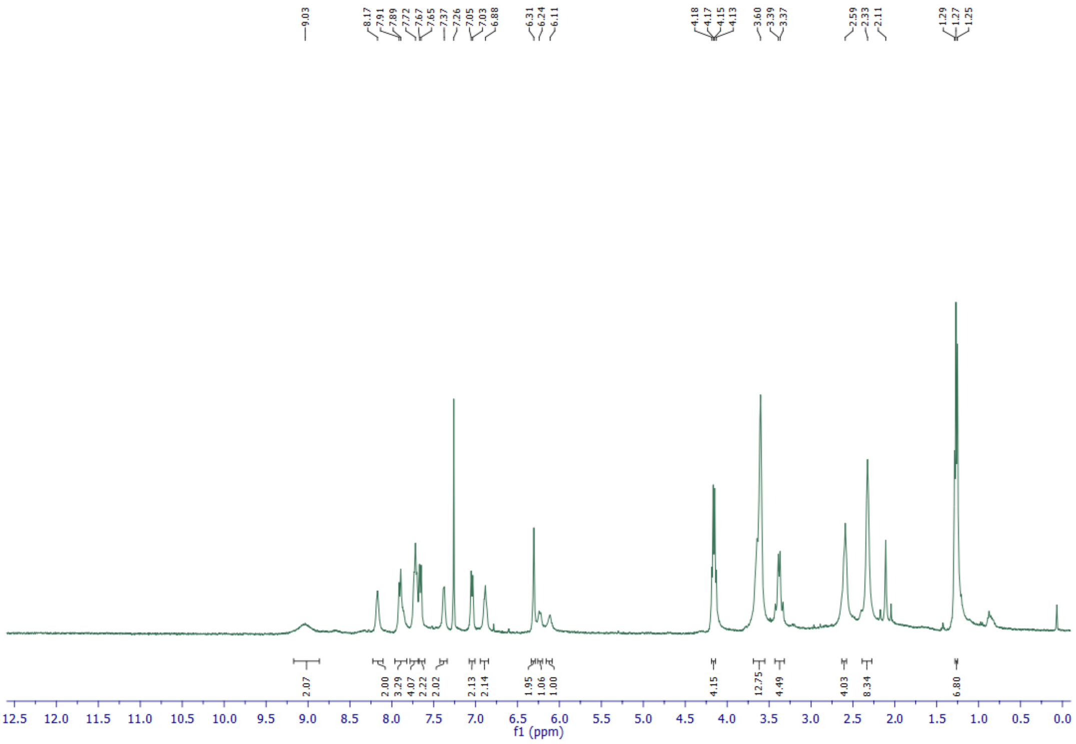

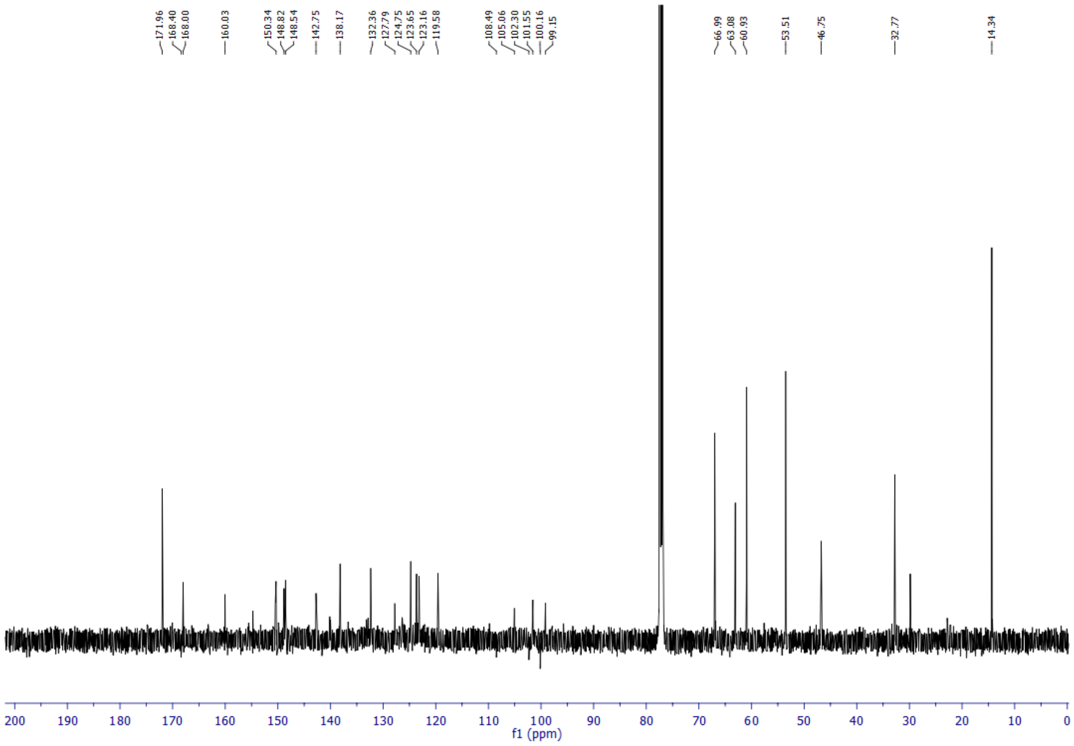

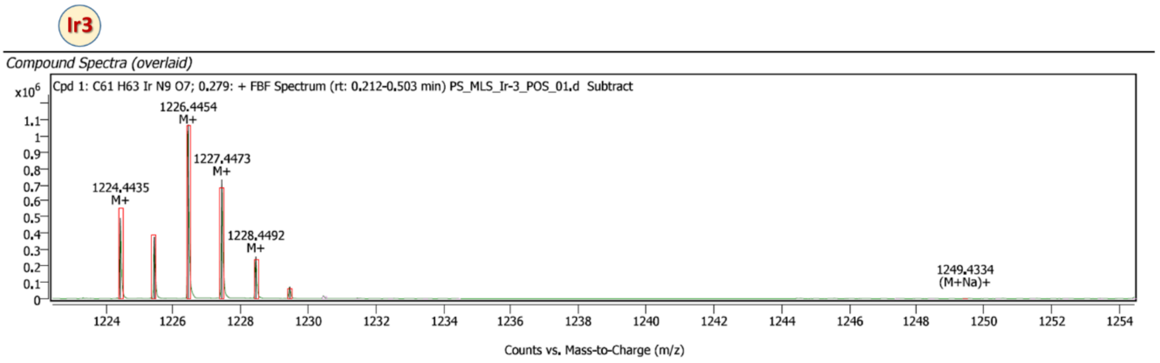

Compound ID Table

Name	Formula	Species	RT	RT Diff	Mass	CAS	ID Source	Score	Score (Lib)	Score (Tgt)
	C61 H63 Ir N9 O7	M+ (M+Na)+	0.279		1224.4428		FBF	91.96		91.96

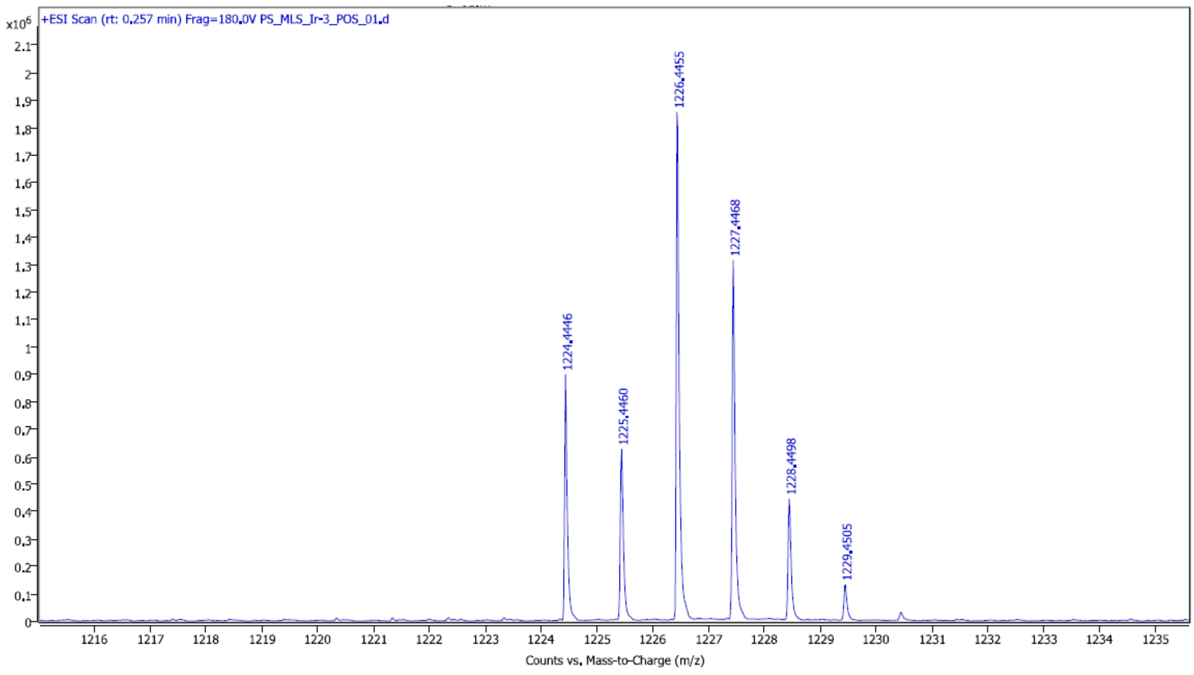

Figure S46. ^{1}H, ^{13}C NMR and ESI HRMS spectra of Ir-3 complex.

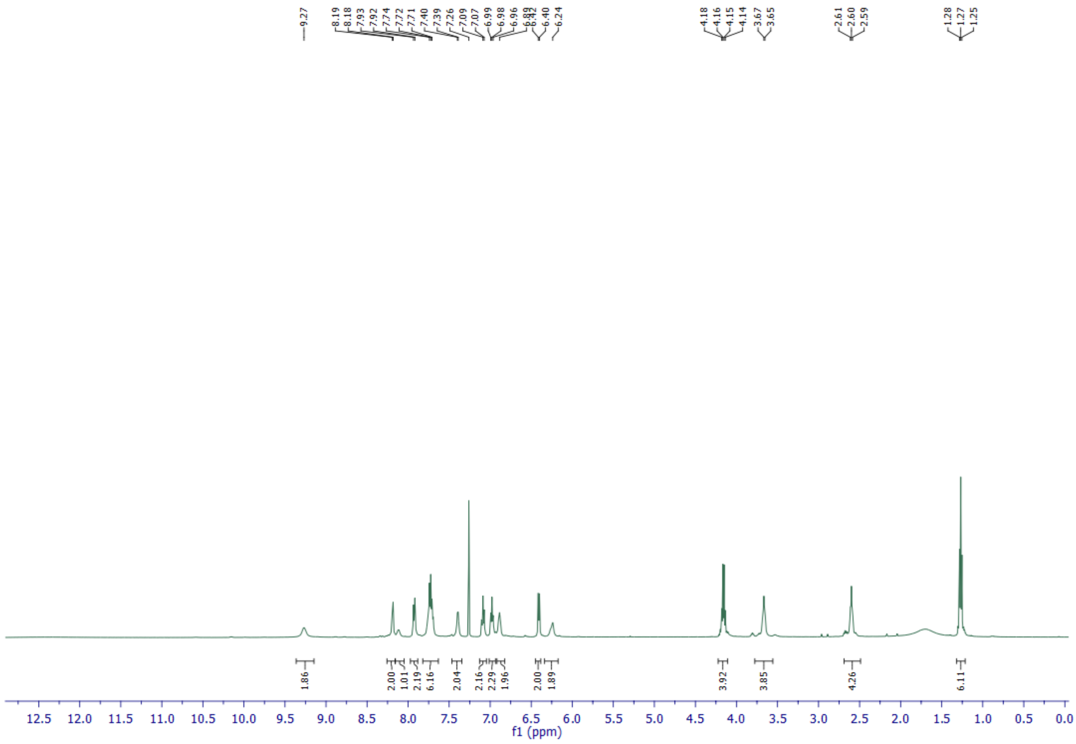

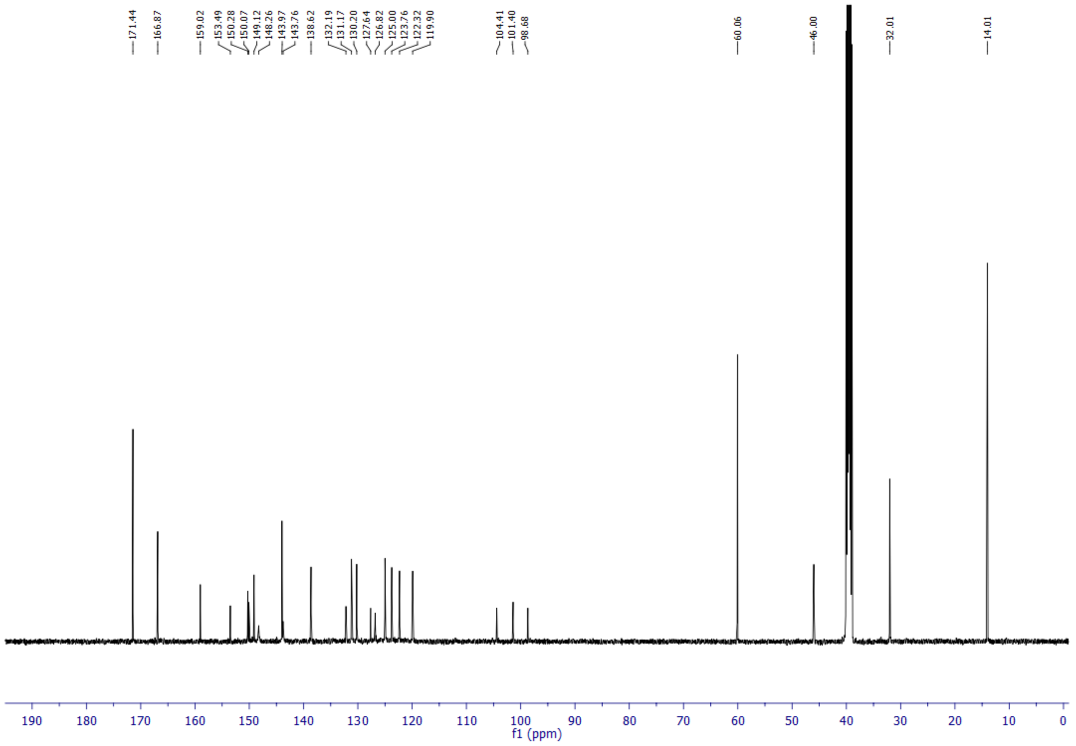

Ir4

Compound Spectra (overlaid)

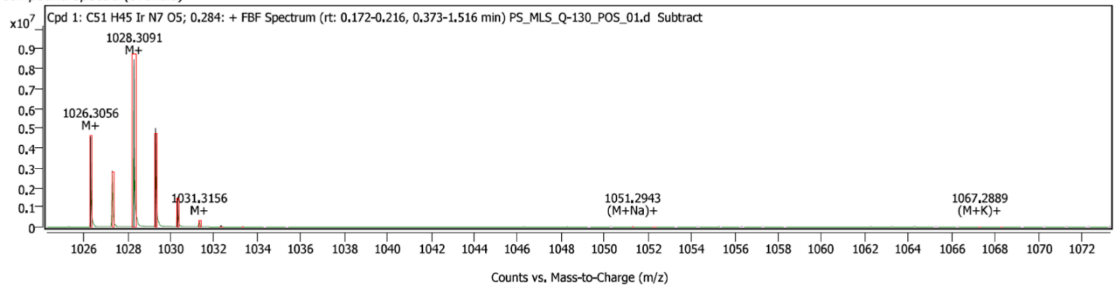

Compound ID Table

Name	Formula	Species	RT	RT Diff	Mass	CAS	ID Source	Score	Score (Lib)	Score (Tgt)
	C51 H45 Ir N7 O5	M+<br>(M+Na)+<br>(M+K)+	0.284		1026.3061		FBF	93.43		93.43

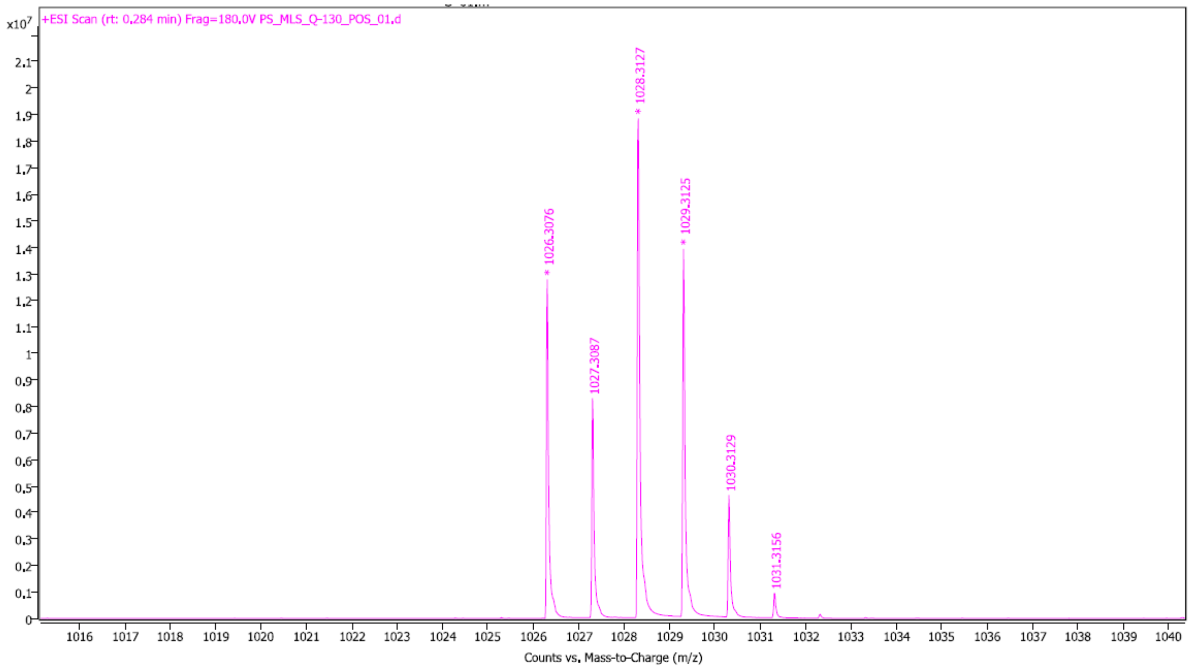

Figure S47. ^{1}**H,** ^{13}**C NMR and ESI HRMS spectra of Ir-4 complex.**

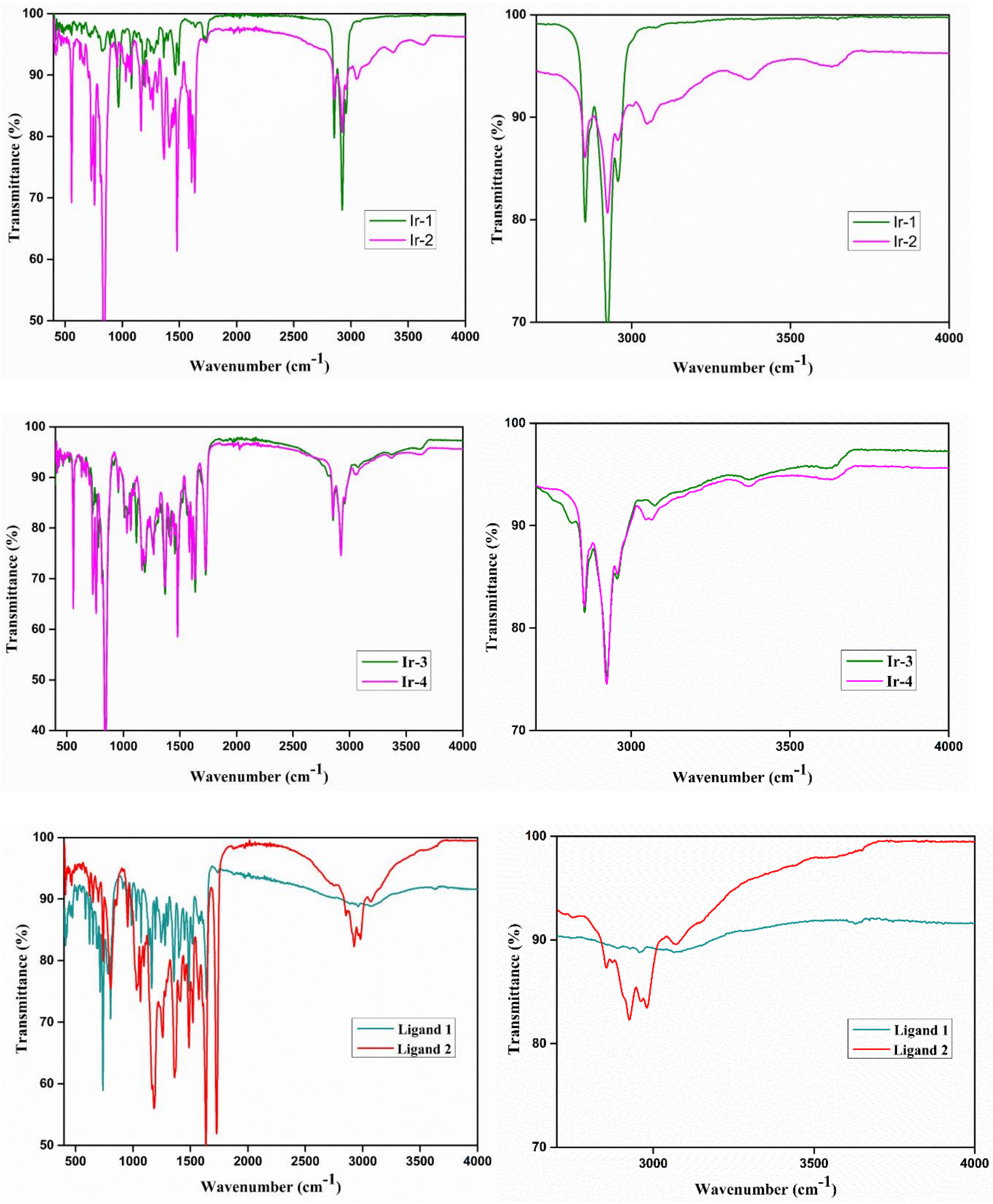

Figure S48. IR spectra of all complexes Ir-1, Ir-2, Ir-3, Ir-4, Ligand 1, and Ligand 2. Inset: expansion of the region 2500 to 4000 cm^{-1}. The weak -OH stretch is not evident due to its hydrogen bonded nature.

Coordinates

1. Ir-1-S_0

Nimag=0

123

Coordinates from ORCA-job opt

C	-1.91928073965579	1.53905017615379	-4.51297536536658
C	-2.71204426927463	2.65294703670447	-4.81752814784794
N	-2.72796867773470	1.57542716142847	-2.25806848381665
C	-3.50792834948902	3.21086989393815	-3.82486387536486
C	-1.95830167593537	1.03511287187073	-3.22020441546101
C	-3.51437258226775	2.66032992124307	-2.53214110573044
C	-5.32820527169005	4.10315863590040	-1.51867081096820
C	-6.17320557365073	4.35205593934422	-0.44652413860479
C	-4.32787348053780	3.11712071980141	-1.41060093804356
C	-6.01486161037643	3.64860645907286	0.76232638779967
C	-4.96369703477668	2.73441208398159	0.88883566787785
C	-4.12446756824005	2.42317157235186	-0.19000159834905
Ir	-2.80907867257267	0.89434803904379	-0.30045354392025
C	-6.58160078577710	-0.87040494577756	-1.58791521477303
C	-7.00177990981481	-1.49856644988150	-0.40082694745816
C	-4.48904692989740	-0.21002716327877	-0.51142180555080
C	-6.18572469419512	-1.49196170278995	0.72103918415466
C	-5.31817565851640	-0.27480885238688	-1.64046464294902
C	-4.94784419244170	-0.81979302287718	0.68475983307308
C	-4.35384129754558	-1.10471182431586	3.14949847426684
C	-3.51029192434461	-0.76771308952680	4.20087706444833
C	-4.11827324789165	-0.59059297538610	1.86321177988537
C	-2.42708509927356	0.08755325455274	3.96225365187458
C	-2.23574366962975	0.56616313267041	2.67326689195345
N	-3.05092555818732	0.23866003429945	1.65429883180802
H	-6.53753539783672	-1.96392786205468	1.64131483419379
C	-0.48693988712901	-2.90815024994427	-0.81907279674894
C	0.82290403477472	-2.50190285304499	-0.63389496056685
N	-1.28162432708856	-0.67303469676340	-0.49811164785512
C	1.10008332123866	-1.14097147317628	-0.36474377051911
C	-1.52269704901282	-1.95857636328230	-0.74341209052954
C	-0.00751153093354	-0.24225608873490	-0.29985147894741
C	2.38841591030945	-0.57255839460872	-0.14779305465735
C	2.59343314353828	0.78870654040235	0.11458274535030
C	1.48907069208998	1.70189067127589	0.17445677262735
C	1.60934807215553	3.08643581160317	0.42024651691095
C	0.18807382393131	1.16789517133087	-0.02833650206739
C	0.46642302957579	3.86759784381419	0.45336650695024
C	-0.78900621349070	3.26353112538674	0.25365278892180
N	-0.91937209252002	1.95838457713258	0.02919329547350
N	3.92411441504227	1.04057107895048	0.28720196456636
N	3.64997638997599	-1.13215721161744	-0.13025822821072

C	4.56058090424395	-0.12889168444976	0.13923973688616
C	5.98373171568804	-0.30657780943131	0.26236594314135
C	8.80883572244573	-0.61678766439720	0.57919504804602
C	6.80608047193272	0.80252717297046	0.63159584283360
C	6.62278517653628	-1.54603415116487	0.04348865736392
C	7.98459573805922	-1.71432440960033	0.18364589395656
C	8.18479600983311	0.63043935018077	0.78413724951538
H	6.03278649593606	-2.41691792419694	-0.25619304809959
N	10.16628222290729	-0.78049461057002	0.76368103432624
O	6.30483385210578	2.02050786904535	0.84171669658782
H	5.32658575209070	1.99340857258214	0.69020400015554
H	3.87889078451610	-2.10825580506075	-0.26190042160834
H	8.41104185656363	-2.69640761990454	-0.00637450721624
H	8.74494059119788	1.51897314478533	1.06631163622592
C	10.82104690616996	-2.03734117343599	0.43448372691932
C	10.99996502278008	0.32872436516975	1.20060883487391
H	10.46924156303385	0.92155945889136	1.95942624754354
C	11.43219682895706	1.24502233364629	0.05441543019621
H	11.89405490907272	-0.07815146629437	1.69426476542725
H	10.23786797066983	-2.88846620478152	0.81665790455458
H	11.78922974435163	-2.07181027910741	0.95276415374064
C	11.04469556418200	-2.21271833406176	-1.06912034866406
H	10.10639291264079	-2.09336037194924	-1.62708964446297
Cl	11.67529400744283	-3.86242958585379	-1.41335978722409
H	11.77657061152583	-1.49078408903776	-1.45582051822646
H	10.56569475613634	1.63161123254958	-0.49867536496809
H	12.09568402201663	0.72441238919856	-0.64960553808284
Cl	12.33839949741124	2.65546769584358	0.70677271991656
H	-5.01774361448100	0.22177258382801	-2.56655379260429
H	-7.99590389685902	-1.94811471297183	-0.35424997157326
C	-7.57483777907234	-0.68963718353252	-2.71506527452060
N	-8.57080034014975	0.28754999140044	-2.31998638901903
H	-8.09269643725953	-1.64163455899225	-2.91512901727926
H	-7.03957646002287	-0.40452847011406	-3.65011414802813
C	-9.83348422181158	0.26862228982664	-3.02789722075300
C	-10.89185228069866	0.96669639639125	-2.14672122718901
H	-10.13948728420567	-0.77217838244462	-3.21510428473385
H	-9.76146284007968	0.76144071892185	-4.02539339323747
C	-8.06122890583533	1.64366472617604	-2.18157866198284
C	-9.22327276326046	2.60861943254934	-1.84908748765615
H	-7.55332873715953	1.98629325233693	-3.11498819619022
H	-7.30324237843239	1.66749699457877	-1.38618383503280
O	-10.30870641356546	1.91595571276208	-1.27362624860946
H	-9.55229433801420	3.15010401738600	-2.75887789712286
H	-8.89677168921546	3.34289618040249	-1.10071195988149
H	-11.39473480963540	0.23357121584294	-1.49800122508904
H	-11.66018246704945	1.44552141746430	-2.78680003293674
H	-4.85580648646018	2.19661847362264	1.83389824227568
C	-7.06799102246966	3.76824713513910	1.83843322694892
H	-5.47938814960991	4.64284742866905	-2.45641091019391

N -8.28397077222379 3.06595143478298 1.44805824818394
C -8.16445524928151 1.60083068921254 1.35093502876186
C -9.44580807465769 3.47428552989329 2.24472435838555
C -10.51663751150616 2.39356847577018 2.20211437071638
H -9.85256647449151 4.42735051396775 1.86152948067331
H -9.16923423326726 3.64394264320777 3.30845684696265
O -10.06534312022771 1.18917476919050 2.81003271432222
H -10.81805721428691 2.21806962931191 1.15286745250664
H -11.40309543636711 2.72117029049223 2.76648845705821
C -8.69392135584985 0.90221667438480 2.59679596267163
H -7.11796664564844 1.32041354419506 1.17442646365148
H -8.73650077103460 1.24047786854258 0.48290452901333
H -8.09824774005006 1.20144960141100 3.48515018086603
H -8.58940512006443 -0.19154429433744 2.48939997880216
H -1.71597573575263 3.84174650743323 0.27929621264645
H -2.57026860455198 -2.23509306283496 -0.88453354506754
H -0.72935718550671 -3.95204269231442 -1.02432780908381
H 2.60008696253351 3.51674807841986 0.57692066861994
H 0.52116807520794 4.94214340021905 0.63528713132786
H 1.63575303675965 -3.22915797321728 -0.69476684227741
H -5.20487451761467 -1.76531905833049 3.31338364583925
H -3.69436971829580 -1.16397978469678 5.20200604562393
H -1.41349098987579 1.24125065832646 2.43440509628119
H -1.74394028826891 0.38395675996119 4.75918706558224
H -1.36540830651327 0.16723612922663 -2.93030670828362
H -1.28344087393462 1.06601303040470 -5.26236181111023
H -2.70807839058248 3.07900888186510 -5.82334039893848
H -4.13385738399452 4.07674960764989 -4.03897501731295
H -6.98926744071490 5.07102253318971 -0.54610874325612
H -6.65909240870231 3.41194486284678 2.81136671596413
H -7.33170266723846 4.82930515821149 1.97909995379146

2. Ir-2-S$_0$

91

NImag=0

Coordinates from ORCA-job opt

C -2.91670802290849 2.03080476621003 -3.62314328758075
C -3.79020265468047 3.11727959419238 -3.48997335188283
N -3.49963713885742 1.29052534940698 -1.42145812138270
C -4.50921510021858 3.27010307222775 -2.31061392159157
C -2.80225966933122 1.14311450928613 -2.56223977685934
C -4.36016293461863 2.34124867497685 -1.26754866343039
C -5.96469297734477 3.38114309354849 0.38985983983152
C -6.59318951604719 3.32812567344517 1.63106368334318
C -5.06133006180892 2.37009059713035 0.01522339998876
C -6.32161723271756 2.26100304015967 2.49813226368747
C -5.42435633037175 1.25361590458311 2.13056399598536
C -4.77151093741396 1.28663467279589 0.88908028943501
Ir -3.39228016435261 -0.00646672907556 0.18973619018282
C -6.51779970794959 -2.11946188451861 -2.00769113759851

C	-6.81176464535938	-3.17156278317042	-1.12956768444521
C	-4.86393032970706	-1.22425058883846	-0.45617504443356
C	-6.14355566567648	-3.25574397775225	0.08900179804910
C	-5.55851118702238	-1.15858839481628	-1.67372537715684
C	-5.17791705644029	-2.29125226677171	0.42966331776652
C	-4.59663136621721	-3.21396454079710	2.73941862346984
C	-3.83150616986120	-3.09713172814382	3.89378500535972
C	-4.43173279102913	-2.29810246571920	1.68742840123177
C	-2.89535798605685	-2.06036609274662	3.99245094947591
C	-2.76786648663144	-1.18356018817308	2.92387017775065
N	-3.50992124189370	-1.29610742792475	1.80746575422926
H	-6.37714569921133	-4.07499716901665	0.77279194334434
C	-0.67872492358852	-3.11116355216300	-1.58767314587305
C	0.58285141184878	-2.63562547444015	-1.27683870721638
N	-1.70826242719234	-1.22124899913130	-0.53800093799794
C	0.71448613292714	-1.41647585633648	-0.57159351102711
C	-1.81106028090573	-2.37141805535721	-1.20006831766577
C	-0.48398839359703	-0.73025918115205	-0.20723049583335
C	1.93947491533247	-0.79743272137183	-0.18982610945310
C	2.00200875033106	0.42130526698867	0.49822801396418
C	0.80588343883120	1.11644335810548	0.87520137332710
C	0.78413283543559	2.34369309410264	1.57169188567907
C	-0.43567782132029	0.52149143359165	0.52293575373305
C	-0.43530631047799	2.91813859755593	1.88751094665150
C	-1.62368563516348	2.26267911701389	1.51559009550718
N	-1.61960019978176	1.10304321838315	0.86223499435683
N	3.30262671949789	0.77260806629267	0.71865802045746
N	3.25591336023111	-1.16875291601985	-0.37621997869374
C	4.05952271649887	-0.19553307765860	0.18523155887113
C	5.49899049916645	-0.20530653912753	0.19579651749486
C	8.35800933401394	-0.15230554316783	0.21276924747683
C	6.20860133305498	0.88824873774052	0.78199917649165
C	6.26317342663082	-1.25173516904213	-0.36409814021147
C	7.64268359328078	-1.24435586853737	-0.36669734286883
C	7.60609307469571	0.89975073972464	0.77365778817860
H	5.76064498286321	-2.10965700253572	-0.81987855538318
N	9.73762234041626	-0.13523686058017	0.22923980122575
O	5.58231528985685	1.92284085599028	1.34377180488462
H	4.60457760750736	1.78029483348037	1.27406677134107
H	3.58809174721508	-2.00064858874323	-0.84536458234486
H	8.16798440120577	-2.08192625185586	-0.81963670034024
H	8.07569414966848	1.76807862541799	1.23014517907663
C	10.50776428979232	-1.22078276699029	-0.35671377874215
C	10.46028707233735	1.02393916804618	0.73082224670769
H	10.01261811994314	1.37520995289575	1.67260016864622
C	10.49913090714320	2.17873083739142	-0.27251273523062
H	11.48785495743145	0.71783774075316	0.97137163834119
H	10.05111741204084	-2.18911776599337	-0.10559020997667
H	11.50797695936377	-1.22624621474136	0.09911397754377
C	10.64543838995259	-1.10450275647187	-1.87636053084629

H	9.66410121588947	-1.00437610847176	-2.35928054173715
Cl	11.42946176233653	-2.5816115598159	-2.54129680819572
H	11.26581014249043	-0.24393142267100	-2.16142145914638
H	9.48819466327626	2.46700904712515	-0.58955310754073
H	11.08649646757124	1.91835902236458	-1.16361676930005
Cl	11.26549583009994	3.62254790248739	0.47809363080142
H	-5.35806150844554	-0.34540535171414	-2.37476008726058
H	-7.56293515220573	-3.91808174337843	-1.39583184634306
H	-5.23963373038566	0.43075944659995	2.82454916813491
H	-6.18079011417513	4.21233211069984	-0.28520640857985
H	-2.60621481367477	2.67772676416396	1.75329797441786
H	-2.82477028760804	-2.70875542615074	-1.42844535963130
H	-0.80981034015805	-4.04873524517427	-2.13027686373752
H	1.72789471237701	2.81692883706722	1.84856278526564
H	-0.49202677084814	3.86699405704736	2.42345319622404
H	1.46997966780601	-3.19893050411774	-1.57505508399544
H	-5.33503940087851	-4.00974485106598	2.64488139338379
H	-3.96382617413326	-3.80545948892269	4.71470749703293
H	-2.06154852649923	-0.35325080547126	2.94776173333189
H	-2.27705603355806	-1.92834734720173	4.88124495328966
H	-2.14272099173440	0.27617180297560	-2.61168679117371
H	-2.33560931415546	1.87036942225170	-4.53212831509274
H	-3.90960774221500	3.83636216969216	-4.30347800508741
H	-5.19849016766686	4.10519346204688	-2.18845638016342
H	-7.29649008887313	4.11054753543627	1.92350366093332
H	-6.81984531358171	2.21325179672801	3.47026871416425
H	-7.04780558381001	-2.04642056892026	-2.96130642701580

3. Ir-3-S_0

Nimag=0

141

Coordinates from ORCA-job opt

C	-1.71872754728449	1.65806254741335	-4.18882100749238
C	-2.48779560949357	2.79624360235360	-4.46318681848670
N	-2.53752127544076	1.64368417661216	-1.93739544101437
C	-3.27775421151554	3.33989578293376	-3.45805954740103
C	-1.77407496868164	1.11692612927055	-2.91173720343183
C	-3.30156459016949	2.75155149426540	-2.18197083843036
C	-5.09847559192183	4.19622351266880	-1.14169056184010
C	-5.98214453453178	4.39661898438580	-0.09165373663858
C	-4.12364747299205	3.18162470148674	-1.05672705383777
C	-5.88731512889341	3.61466864317015	1.07508514977551
C	-4.85718028316422	2.67739665963410	1.19016533781069
C	-3.97526931321502	2.42016494224674	0.13094385369217
Ir	-2.64582464244168	0.90546530054310	-0.00115750609801
C	-6.36392748562273	-0.87853859815618	-1.41458277064070
C	-6.81051033420720	-1.52138329789337	-0.24567877383375
C	-4.30644921503559	-0.21363979312351	-0.27296954644374
C	-6.02457446006108	-1.52283175710665	0.89874942160482
C	-5.10044783747744	-0.27727587528094	-1.42582767279347

C	-4.79048071424175	-0.84481647493620	0.90280247112925
C	-4.23230422729692	-1.19300382686920	3.36835702526157
C	-3.40364759671967	-0.88384749496322	4.43991023440787
C	-3.98164404080273	-0.64215884749198	2.10027643059722
C	-2.31954112875960	-0.01988310670825	4.23979735012976
C	-2.11243595970744	0.49503910927535	2.96732594273067
N	-2.91356454003817	0.19467048752628	1.92912185594821
H	-6.39525503093409	-2.01332086655534	1.80190318213568
C	-0.22480023689877	-2.82507374801106	-0.59974065607000
C	1.07556999796664	-2.38426905083213	-0.42791993605604
N	-1.07919883217177	-0.62146103512559	-0.22007910136673
C	1.31844203842623	-1.02140807938602	-0.13432763136900
C	-1.28676945434890	-1.90795900611430	-0.48957475149882
C	0.18480013973333	-0.15822939173501	-0.02928546862750
C	2.59337332028474	-0.41878415610699	0.06585488658375
C	2.76179331682282	0.94300735615403	0.35273863769000
C	1.63115398720503	1.81744662310914	0.46641752454599
C	1.71486418242638	3.19714364336969	0.75179229713194
C	0.34304922043444	1.24919836914794	0.27623215396489
C	0.54991905581398	3.94080024703466	0.83519970978615
C	-0.69052529026200	3.30471043843424	0.64113183834521
N	-0.78584439651962	2.00397512032171	0.37791119066143
N	4.08658653792925	1.23704570207172	0.48894696943128
N	3.87345309949274	-0.93527260488957	0.03370344179803
C	4.75942278308791	0.09368001632460	0.29366156830812
C	6.19039160543633	-0.03092633491647	0.35151858594129
C	9.04073032976534	-0.22226973930193	0.49408830115504
C	6.98935582457756	1.11630350045184	0.65471728286179
C	6.86568953116230	-1.24925352583054	0.11301034456953
C	8.23842176724717	-1.36219746888690	0.17084378201919
C	8.37984969173855	1.00554538513227	0.71676326638213
H	6.29345771986593	-2.14774792381520	-0.13633048472308
N	10.40829612765995	-0.32217750676498	0.58764020999985
O	6.45108331701686	2.31667231882462	0.88186539397631
H	5.46725162605301	2.24489071812083	0.79827065435989
H	4.13246734822438	-1.89706637764809	-0.13986602831072
H	8.69478661267354	-2.32662420649132	-0.04108426508122
H	8.92097110502867	1.92234506260580	0.94103258653826
C	11.09082107527629	-1.55810671127126	0.24350541360466
C	11.21404874398155	0.86809832949269	0.81093418115800
H	10.76007840692490	1.46923285802988	1.60959193826389
C	11.39437191749909	1.73920154553609	-0.44712860583513
H	12.19727534497590	0.54665412404959	1.18369897834055
H	10.55788107294662	-2.41026447131459	0.68562872177345
H	12.08384062630951	-1.53760944653872	0.71563028300498
C	11.24585550587952	-1.78153372361519	-1.27362305537323
H	10.27046027182850	-1.61520070063734	-1.75977768017862
H	11.96897284893265	-1.08072500467833	-1.71088262335875
H	10.41894124938191	1.84071324349478	-0.95159013609801
H	12.09503448804120	1.28450382077877	-1.15979017735833

H	-4.76949757281308	0.21787613476585	-2.34192497079329
H	-7.79572719397629	-1.99355835739677	-0.23335611652417
C	-7.31327274385257	-0.69351673756586	-2.57906681604384
N	-8.10707453455244	0.50445848318787	-2.37543146507506
H	-7.99720655085066	-1.55382002531863	-2.63794284393530
H	-6.74073677039882	-0.66106689069899	-3.53465699181676
C	-9.42387493676184	0.53965301308470	-2.98260965668838
C	-10.26817507295278	1.61451142365074	-2.27745336887199
H	-9.91294563151901	-0.43797744397259	-2.85208115405295
H	-9.38013398311383	0.72366491378438	-4.08063070307833
C	-7.37103951703740	1.75167877360534	-2.54476338474579
C	-8.34886950501251	2.92865430918431	-2.68775078037831
H	-6.70875949080528	1.72204087401235	-3.44291493047656
H	-6.71556222980711	1.91163835224976	-1.67474607145790
O	-9.48620657812131	2.73755115970176	-1.88720815945275
H	-8.63265784017599	3.07126790355741	-3.75070365599777
H	-7.86565395866659	3.85612728146231	-2.34943135233245
H	-10.69731981939882	1.21994880594991	-1.34512409857319
H	-11.09267283830100	1.93480775349172	-2.94528102195271
H	-4.80142827751980	2.07855073709948	2.10228314384123
C	-7.00460488824392	3.67372328637551	2.09538034292162
H	-5.20309933698449	4.79261511175583	-2.05121635828762
N	-8.19589665355658	3.07900268270544	1.53183787526976
C	-8.09338129830369	1.65306227138580	1.19938967062023
C	-9.46502413700866	3.47296271052142	2.11024269407754
C	-10.59786709044969	2.95045017695158	1.23290498813460
H	-9.52598644539820	4.57393076194055	2.13302627012535
H	-9.59814991909825	3.13111253398048	3.16285768404028
O	-10.52964126032346	1.53954483404658	1.02221931882799
H	-10.53629527396292	3.44254035888950	0.24801230423723
H	-11.57202491556664	3.18776986799200	1.69603355338725
C	-9.38127915398199	0.91209153448687	1.54698857236139
H	-7.26648081130507	1.16980091264968	1.75554075230655
H	-7.88298737994598	1.51721899472669	0.12756897206338
H	-9.46903026142979	0.79243902113123	2.64621378223786
H	-9.34495638912021	-0.09825934298782	1.11219495388834
H	-1.63357742855369	3.85366864584227	0.70002999564552
H	-2.32764479705071	-2.21147327632398	-0.62498430257819
H	-0.43998043001682	-3.87125491616720	-0.82305326288854
H	2.69576818346001	3.65237870804971	0.89952646692498
H	0.57564647606528	5.01029283674313	1.05024650751221
H	1.90816108150161	-3.08564126144676	-0.51828724692694
H	-5.08250783130300	-1.86164354090893	3.50148734160418
H	-3.59914058254059	-1.30936646928740	5.42679652463413
H	-1.28796625413490	1.17716224208686	2.75733858115171
H	-1.64715558791081	0.25476569253882	5.05349689020703
H	-1.20079798071885	0.22844687282890	-2.64568402948064
H	-1.08947656022151	1.19437530774831	-4.94949108539830
H	-2.47148609867184	3.25113350289634	-5.45617870852685
H	-3.88848369589184	4.22159342424945	-3.65019237106010

H	-6.79128657204493	5.12443633717842	-0.18288433142286
H	-6.67393530914247	3.18049024105962	3.03957348199798
H	-7.23072069841258	4.72395876673908	2.34445524916900
C	11.69545783821428	-3.18502410612114	-1.61582382693231
C	11.88308413354972	3.14042847948816	-0.14764652801034
O	12.80503581355147	3.68898792591114	-0.69515996000319
O	12.67058671171648	-3.46929529021004	-2.26281375419750
O	10.84686326370068	-4.09589346612370	-1.08921337625530
O	11.12734328723739	3.71132590255238	0.81433023647362
C	11.13565430745959	-5.48824459139224	-1.33469423795025
H	10.65881461059166	-6.02244531229318	-0.50045280497291
H	12.22416878855875	-5.63674683156147	-1.28348261537662
C	10.58655966975642	-5.94039679031331	-2.67555572390825
H	11.09451390757294	-5.41756998311684	-3.49930622577913
H	10.75213636914998	-7.02206523069508	-2.80313724409256
H	9.50427336506155	-5.74667303023615	-2.74310088640751
C	11.43052887050390	5.07441336240533	1.17682764725517
H	11.05504921930802	5.17835616347743	2.20469652165268
H	12.52264555941824	5.20462847101348	1.17447069375972
C	10.76037255871020	6.05763900637364	0.23459324083847
H	10.94516162440074	7.08866922678937	0.57595702796664
H	11.16527962399996	5.95746286628853	-0.78333566305002
H	9.67181793624232	5.89304925027598	0.20501984288884

4. Ir-4-S$_0$

NImag=0

109

Coordinates from ORCA-job opt

C	3.77571254640378	-0.17775622338552	-4.37133120264661
C	4.66257057306274	-1.19644788346643	-4.74127693265470
N	4.46673053855055	-0.40126398643911	-2.08894102884672
C	5.44169398107835	-1.80546887870193	-3.76503457507272
C	3.71081606916909	0.18698914851739	-3.03353850079488
C	5.33948326677271	-1.39846885657537	-2.42467807708825
C	7.02666132670714	-2.99700448083402	-1.42590148721267
C	7.70974167603635	-3.45822924616604	-0.30391356877985
C	6.10081205351666	-1.94531020512831	-1.30268971654177
C	7.47026368296938	-2.86511362203621	0.94308406200702
C	6.55183657252867	-1.81859539705094	1.07053931212136
C	5.84476497881434	-1.33905867339554	-0.04255205426619
Ir	4.43589211910829	0.10304010499961	-0.07916033597072
C	7.48974571115000	2.97077323333185	-1.31283845629232
C	7.84147933051399	3.54664823981677	-0.08447443458807
C	5.88831460884619	1.49573779160109	-0.21577892267080
C	7.22569444473040	3.10363402482038	1.08298854624519
C	6.52723409139845	1.95842920813957	-1.37618515071883
C	6.25641380881351	2.08618460836030	1.02438012606631
C	5.78076120749095	1.94935353448741	3.52972038862084
C	5.05955748353864	1.35633572913782	4.55918451671574
C	5.56173831494007	1.55914674208160	2.19830948437507

C	4.11354208670933	0.37055768634516	4.25233258223869
C	3.93246644123435	0.02220905121159	2.92085912418329
N	4.63136417434324	0.59598197606290	1.92486550005930
H	7.50427417515417	3.55323823484238	2.03888549205335
C	1.66878994747833	3.63050681821128	-0.29860264303516
C	0.41977004791731	3.05495909777048	-0.14892514287080
N	2.73124293754165	1.48907605157859	-0.16406439240827
C	0.31081429066774	1.65239170396728	0.00306499831150
C	2.81232882764029	2.81039434977402	-0.30191016617403
C	1.51995560143577	0.89120209703777	-0.00589507159679
C	-0.89871721527421	0.91878343680277	0.16504462783390
C	-0.93901204434721	-0.47542095186892	0.30326730688542
C	0.26764119438548	-1.24989594285391	0.29657364365084
C	0.31268483634641	-2.65400107014111	0.43191125724113
C	1.49544105453630	-0.54949684880972	0.14842922623771
C	1.54018487902995	-3.29408756427993	0.41627475890872
C	2.71442481499312	-2.53121206407519	0.27558823984663
N	2.68886371885316	-1.20636238859922	0.15282187179787
N	-2.22845650278794	-0.90009952251165	0.44002190121531
N	-2.21812583125062	1.31955638136546	0.22401156747487
C	-3.00244668125842	0.19393811357012	0.39309801975475
C	-4.43526986348830	0.18924622609814	0.50911219824418
C	-7.28245875613478	0.12658407440662	0.76772787720766
C	-5.12855945274463	-1.04804904175007	0.69176195344056
C	-5.21262017331721	1.36784404608348	0.44726978574713
C	-6.58598147201395	1.35976649850835	0.56481499326650
C	-6.51995168997976	-1.06121724552252	0.80972385052052
H	-4.72365367539566	2.33456083757921	0.29489474631484
N	-8.64837091440229	0.10272698199606	0.92090847850203
O	-4.48776221655142	-2.21781414819945	0.75174536253119
H	-3.51657287369571	-2.05394416169677	0.65457059990538
H	-2.56568830786990	2.26718814958733	0.16534974934314
H	-7.12428864827497	2.30224898574247	0.49084621197587
H	-6.97927103615765	-2.03964730026318	0.93378406347764
C	-9.43404879181776	1.31595516328089	0.77599066676532
C	-9.35373517946269	-1.16675164680397	1.00483379082071
H	-8.82373569265353	-1.83276718540600	1.69857866626401
C	-9.52182829515242	-1.87117760966425	-0.35558721328223
H	-10.34131173176433	-0.97581911541312	1.44892705128863
H	-8.94481378098596	2.13768421470366	1.31657680802008
H	-10.40189386357902	1.15311396497760	1.27177875045435
C	-9.67192787987497	1.73579113799025	-0.68967138481792
H	-8.71459842759056	1.68792810359035	-1.23253445230998
H	-10.38602738576154	1.06500484896001	-1.18480709274784
H	-8.55878811702465	-1.83961586811669	-0.89146707959671
H	-10.27373488354531	-1.37081427164009	-0.97996447628984
H	6.28106657468883	1.52241612063039	-2.34696718707542
H	8.59608389527997	4.33475482757053	-0.04096682719700
H	6.39352474030683	-1.37112408217234	2.05419842060008
H	7.21885312601915	-3.45930410955545	-2.39688646074217

H 3.70307320944120 -2.99689943481147 0.26729878789616
H 3.81678719524023 3.22351511244630 -0.42022584014128
H 1.78176445948161 4.70948808479367 -0.41562531126909
H -0.62052968795243 -3.20837358255420 0.54576904945045
H 1.61444205156909 -4.37832119161386 0.51502330172224
H -0.47549041151195 3.68086805325792 -0.14828092172421
H 6.52555514991011 2.71441117250281 3.74720325674610
H 5.23313108219938 1.65546974757237 5.59530982745301
H 3.21588933526144 -0.74426654723266 2.62453346310904
H 3.52788617750861 -0.12392208943223 5.02837118509452
H 3.04313578649770 0.97801758910003 -2.69044418456428
H 3.14684189272357 0.32892438740372 -5.10448639966324
H 4.74446737494883 -1.50968532274186 -5.78449234726428
H 6.14055947458365 -2.59791572566320 -4.03128381037510
H 8.42975569610051 -4.27386540363030 -0.39745966276876
C -10.20261421174141 3.14802830501647 -0.79865896080591
C -9.91742437160566 -3.32711827958231 -0.23120579818299
O -10.84833202382562 -3.84750842964765 -0.79112689624345
O -11.27062528239285 3.46368751266662 -1.25703256014064
O -9.31111673691742 4.02733417540700 -0.28810793203792
O -9.07081804958129 -3.97976993267903 0.59299699059833
C -9.67458534460468 5.42373695648844 -0.31293870219094
H -9.08382213586614 5.87867812674152 0.49501613521236
H -10.74417321774245 5.51366331987833 -0.07262127330889
C -9.36425909756323 6.05691143942912 -1.65729019131666
H -9.98285594900802 5.60998918385724 -2.44929862990601
H -9.58231933448479 7.13619819458593 -1.62189606696784
H -8.30183864653375 5.92789007010155 -1.91796779153099
C -9.29175248402175 -5.39225361269137 0.78924334501932
H -8.83263998581718 -5.61159537695064 1.76361088755984
H -10.37492409867490 -5.57364392582187 0.84996077856757
C -8.66193161083768 -6.21187348025898 -0.32233106658130
H -8.78067985808281 -7.28595539237011 -0.10795360017354
H -9.14976863112308 -6.00028071166340 -1.28522837772940
H -7.58597838545268 -5.99384772290857 -0.40985665201646
H 8.01082290471190 -3.22119337939242 1.82432307584073
H 7.97703321861967 3.31252371738116 -2.23008014161192

5. Ir-1-S_1
NImag=0
123
Coordinates from ORCA-job opt
C -1.75269746314607 1.52328307373297 -4.35620422948704
C -2.45361006563847 2.69112398877890 -4.64720649741816
N -2.68581141115793 1.50607495806979 -2.16840327619540
C -3.27026259591500 3.25353174942396 -3.67757451682123
C -1.89368197010063 0.96100815951235 -3.09855962257916
C -3.37723501935608 2.64303791700733 -2.42753971704764
C -5.16420769808939 4.12176011530293 -1.43076353469898

C	-6.02121050947755	4.38066175614165	-0.37301028765217
C	-4.21138338957814	3.10606483079970	-1.31585927832941
C	-5.92222956418244	3.66292236922863	0.83254586107415
C	-4.91782754056262	2.71554730314771	0.96857771982542
C	-4.07736903729451	2.37998195894896	-0.10465341717168
Ir	-2.86966252626169	0.79585261467161	-0.23539479967386
C	-6.51509203462417	-0.99198989762119	-1.65633641810294
C	-6.96216440150921	-1.62068137026538	-0.47936673342052
C	-4.46143800061729	-0.35435900006877	-0.51937208794106
C	-6.17555978072790	-1.64022516480038	0.66261405197560
C	-5.25442833111525	-0.42082944539454	-1.67871654779663
C	-4.94247358415896	-0.98549373958516	0.66114073260973
C	-4.34406538944909	-1.37355753599713	3.10585684587772
C	-3.50435425528978	-1.06428542190907	4.16614602141083
C	-4.12911960128157	-0.77980277969205	1.86231552118087
C	-2.45992773857583	-0.16456746943365	3.96948778343372
C	-2.29131879005268	0.39325117447955	2.71246612318573
N	-3.10617763983981	0.09399607911316	1.69526276599432
H	-6.55813157499098	-2.11599181228601	1.56690350113646
C	-0.53280130177036	-2.95623883138181	-0.78668739054664
C	0.79721894218662	-2.51792805438490	-0.59368157520362
N	-1.36025747659688	-0.73573234561586	-0.43946337531493
C	1.03639619124293	-1.18471134736047	-0.31152409611730
C	-1.55922608610332	-2.04471762840967	-0.69976254877012
C	-0.07281561858998	-0.27847054986396	-0.22366376163710
C	2.31768151347642	-0.58083662229737	-0.08624098255187
C	2.50259530728752	0.76045890682463	0.19611420411357
C	1.38980752607064	1.66805884058222	0.29094802358152
C	1.48909098097510	3.01778452288775	0.56935633665211
C	0.09780183433241	1.08869795769160	0.07475939512083
C	0.31770966472193	3.79578280662517	0.63416525847993
C	-0.90261873975671	3.18666476756459	0.42466844583752
N	-1.03246838139987	1.87502103286831	0.16199285157881
N	3.83361578465386	1.02584667819604	0.35826089779539
N	3.58286374869799	-1.12609906098429	-0.09067961742985
C	4.47190252028829	-0.12260861448203	0.18321375169713
C	5.90916587232873	-0.27925003385955	0.28013752950331
C	8.73554256940804	-0.52549358485540	0.50676088774520
C	6.71264365091417	0.84693510597977	0.59089499733116
C	6.55890778862925	-1.50511211232880	0.08137965649141
C	7.92587009296454	-1.64559209190580	0.18222856317430
C	8.09696455529088	0.70837736544039	0.69537893368657
H	5.97571327229057	-2.39571498740322	-0.16672749253020
N	10.10443492885364	-0.65189039932298	0.63995461873918
O	6.19401237661703	2.05420507568268	0.78894068350541
H	5.21015517289499	1.99984010269105	0.68402386741383
H	3.82095756533314	-2.09345095733881	-0.25746202491537
H	8.36336722649576	-2.62562589527566	0.01064467849006
H	8.64494016811462	1.61668928772117	0.93292787588934
C	10.76040742404174	-1.91879170600183	0.39762849185634

C	10.92680978290118	0.49621980209226	0.95815689156423
H	10.43926927143623	1.11408104661990	1.72538066238210
C	11.24428498775593	1.36347311623666	-0.25571912357714
H	11.86781188113790	0.14249607113188	1.40072729254550
H	10.18949351995232	-2.74187012720801	0.85051986237148
H	11.73579079435333	-1.91209468750363	0.90239540456878
C	10.96857815597616	-2.21147725087345	-1.08489617570655
H	10.02204706336923	-2.15283036448966	-1.63639366302365
Cl	11.62584657444361	-3.85799098428699	-1.30056231146416
H	11.67815029827314	-1.50724837319238	-1.53710111611819
H	10.32709374654461	1.70561229011258	-0.75073706938235
H	11.85334846311968	0.81835690553261	-0.98792170532032
Cl	12.16668515215892	2.80735343080621	0.24591710173132
H	-4.91887365459450	0.07567326392727	-2.59158309803699
H	-7.96064219096578	-2.06106584515022	-0.45899796660116
C	-7.49457126886877	-0.77910224964032	-2.78335364725811
N	-8.45866953435413	0.21343447057397	-2.36934845234635
H	-8.03123926610750	-1.71556701667794	-2.99727365021738
H	-6.95003161368445	-0.49356685153468	-3.70956686877720
C	-9.73050863694667	0.23074375770609	-3.05537265386130
C	-10.75513446908446	0.93693498862525	-2.15565139302931
H	-10.06257625062758	-0.79929319511293	-3.24846708949983
H	-9.66045470561429	0.73057387916955	-4.04590278222013
C	-7.91692848905458	1.55027700406324	-2.20454430408539
C	-9.05664766192224	2.54660715610763	-1.93258612218648
H	-7.35124279985318	1.87703913329470	-3.10700062353508
H	-7.20328001009300	1.55368330014012	-1.36773237384758
O	-10.14456751239385	1.90181370101253	-1.32685081552734
H	-9.37513500546765	3.04177682149615	-2.86967266700446
H	-8.72042344848718	3.31952004593957	-1.22955575043266
H	-11.23093273759884	0.21403320861337	-1.47827604048972
H	-11.54643875779736	1.40079001989783	-2.77398911486685
H	-4.83825301489289	2.16572663890654	1.90901869952882
C	-6.99300883022759	3.83381316450136	1.87909218483273
H	-5.27590046394361	4.68343078273009	-2.35948207403076
N	-8.22854322322503	3.21455656966091	1.43842511346795
C	-8.21925719050786	1.74712212633540	1.41486517736816
C	-9.40750300951357	3.74828531484791	2.11961874274658
C	-10.55564774006361	2.76223829104787	2.02020829897972
H	-9.69887144069367	4.71371478737091	1.67342933964176
H	-9.20402230795115	3.93478225296942	3.19436883014866
O	-10.25960273326419	1.55775688051381	2.70073658751399
H	-10.78132491177972	2.56599614861470	0.95703513610105
H	-11.45410278266177	3.18450057198694	2.49190406315247
C	-8.91124198666409	1.15872252400300	2.63340465197925
H	-7.18567232945879	1.38038068575056	1.35493722066096
H	-8.73837976642701	1.38673752960795	0.51492774221012
H	-8.37565959185112	1.45807078284795	3.55690780325309
H	-8.88490088789508	0.05744679186739	2.58738001871694
H	-1.82992384111417	3.75942941556906	0.47460248772074

H	-2.59487202596496	-2.35177237307806	-0.85140151620709
H	-0.75438578391678	-3.99962210866314	-1.00672511086262
H	2.47190557390396	3.46044735622358	0.73398535789421
H	0.35862990541068	4.86266441085439	0.84965228662386
H	1.62128698923014	-3.22924296664434	-0.66617857616132
H	-5.16359730400517	-2.07918508997501	3.23456624573025
H	-3.66215337274283	-1.52488809155064	5.14299705309463
H	-1.49036728306661	1.09957879037171	2.49561297729369
H	-1.77751213915264	0.10426657624255	4.77551257393844
H	-1.36752862254783	0.05207309939838	-2.80819325853839
H	-1.09896421468202	1.05118939115122	-5.08923628637439
H	-2.36079344212383	3.16321027222975	-5.62693779913683
H	-3.82156144844134	4.17048295882631	-3.88055606325185
H	-6.80434898855850	5.13382183145054	-0.47894107526599
H	-6.63685118023787	3.44215719048759	2.85598100342698
H	-7.18960144977547	4.90680898712497	2.02464848002199

6. Ir-2-S1

NImag=0

91

Coordinates from ORCA-job opt

C	-2.86186087387115	1.95844591021260	-3.62107971738758
C	-3.65456887926499	3.09527661935746	-3.48825798499572
N	-3.51576570213696	1.24799341971793	-1.44781888509589
C	-4.37075458453265	3.29225502590762	-2.31626934684267
C	-2.81646454293281	1.05571250190703	-2.57137745767099
C	-4.28936586448167	2.34936370555769	-1.29234191875714
C	-5.83018509576392	3.46589712375507	0.36762447498496
C	-6.43148187218424	3.45888656327643	1.62320625736625
C	-4.97845059012436	2.42569879632666	0.00235575127854
C	-6.18628770241543	2.41169383962025	2.51726120436048
C	-5.34019398320195	1.37204854445981	2.15946238009166
C	-4.72465529853617	1.34706299879644	0.89562570534909
Ir	-3.45821911624892	-0.01113629303068	0.19159379948375
C	-6.44192514546228	-2.26846674574511	-1.97593056782447
C	-6.69634207507155	-3.30202266746379	-1.06818992427596
C	-4.83170171284727	-1.29429502146712	-0.43991481065751
C	-6.02675312190110	-3.34708491174812	0.15172759803741
C	-5.51838805474350	-1.28077846335237	-1.66760133812716
C	-5.09749196717935	-2.35891764149279	0.46784424959761
C	-4.41760570491577	-3.25895156088276	2.75334625055825
C	-3.63211614457356	-3.10144426772048	3.88648212967083
C	-4.33676617451504	-2.32265142735042	1.72355892653878
C	-2.77138137353798	-2.01083686132168	3.97397835829616
C	-2.72987524291302	-1.11279354445220	2.91986575945507
N	-3.49568220443473	-1.26637096305481	1.83446786014369
H	-6.24061336643525	-4.16049625059878	0.84641176543442
C	-0.77774302467200	-3.10841335660644	-1.58696165711802
C	0.50903348831188	-2.60373116272437	-1.29582940626632

N	-1.80781820011961	-1.21727234472665	-0.53435222610464
C	0.62647125151941	-1.40562008559809	-0.61432237869776
C	-1.88452687648026	-2.39423536642969	-1.19151173327143
C	-0.56235607273190	-0.70379633153155	-0.22050909881781
C	1.85250111315594	-0.76236789509013	-0.24139649082081
C	1.91902353883512	0.43667449029697	0.44401438265301
C	0.72769909580506	1.14087849070585	0.83881974697548
C	0.71118065679897	2.34419639206412	1.51715001759655
C	-0.51199209919916	0.51619378735362	0.48300843917213
C	-0.52542820087716	2.93115353573384	1.84479688684654
C	-1.68984190643155	2.28469592077494	1.48638430061381
N	-1.70834685448629	1.10796910538145	0.83382912620909
N	3.22434102211117	0.78549184807493	0.65252807798959
N	3.16311054565580	-1.13413722008390	-0.44617668109906
C	3.96276286120651	-0.17231213980530	0.10914957515682
C	5.41202088947902	-0.18790410383856	0.11474065342059
C	8.25868276869961	-0.16524760177082	0.17274724430858
C	6.11679647242869	0.86163954981511	0.75701042505782
C	6.16873331822960	-1.19966102689119	-0.49278640468602
C	7.54669194903145	-1.20586469959429	-0.47990244352020
C	7.51185359153394	0.85762832757343	0.77440305162235
H	5.66452743847247	-2.02252352026066	-1.00626874962372
N	9.63906640386769	-0.16602025879165	0.21809586330189
O	5.49321536019002	1.87173091571001	1.35374117883954
H	4.51538889632049	1.74652688911363	1.25461514741677
H	3.48611821917576	-1.96901522431904	-0.91412394648754
H	8.06888123543008	-2.01931442009160	-0.97691648086117
H	7.97915602372948	1.69714870193929	1.28298517526041
C	10.40359471368692	-1.21270818912301	-0.42561588812416
C	10.35886011774593	0.91263835870049	0.86199200093297
H	9.86993335761036	1.18842448188248	1.80725433520542
C	10.49104369826228	2.15476912150385	-0.01344894462233
H	11.36260700078527	0.55503242311698	1.12840473916944
H	9.93949043717726	-2.19322855672632	-0.24817020353577
H	11.39783357057349	-1.25855496133039	0.03879240681933
C	10.56382108337907	-0.99960661468918	-1.92747643091644
H	9.58899444312342	-0.87609716479482	-2.41538484771870
Cl	11.37002750507256	-2.41021455479854	-2.66827801842161
H	11.17509912873826	-0.11438083585147	-2.14382729491536
H	9.50887793007838	2.51714496129053	-0.34108812908323
H	11.10297990978064	1.95435635468028	-0.90210864799694
Cl	11.28076947812606	3.46851250444566	0.90160847610828
H	-5.32994444389827	-0.48176177338748	-2.38614137197453
H	-7.42195863958186	-4.07846247042576	-1.31579388590312
H	-5.15752885215835	0.56355729489287	2.86893063818505
H	-6.03449415005506	4.29139524642102	-0.31561465674501
H	-2.66105577054827	2.71337263958933	1.73495547655687
H	-2.88784873703323	-2.76063649651287	-1.40889808201132
H	-0.90560664647409	-4.04967511861879	-2.11980165365650
H	1.65378750858308	2.82121918665099	1.78711627042919

H	-0.57782071659994	3.87999450360071	2.37716411435183
H	1.39566341680530	-3.15845970633263	-1.60639608351201
H	-5.09396406011343	-4.10764303025464	2.66353321400763
H	-3.68922068669289	-3.82882822520270	4.69826874449514
H	-2.07359913686143	-0.24266072141537	2.92653090453946
H	-2.13441031703572	-1.85307770311139	4.84409261272312
H	-2.21076057622572	0.15050345245310	-2.61050378128411
H	-2.27992912067756	1.76943860466560	-4.52289109219650
H	-3.71201376839129	3.82814151601788	-4.29508646357531
H	-4.99240169681663	4.17745763359349	-2.19084734857152
H	-7.09520918725139	4.27622907877963	1.90972856695717
H	-6.66400169890364	2.41528538632942	3.49884632054878
H	-6.97430809497424	-2.24181770163612	-2.92858121034351

7. Ir-3-S1

NImag=0

141

Coordinates from ORCA-job opt

C	-1.55517540133439	1.53365802210532	-4.23247096265304
C	-2.29461627481802	2.67407444161340	-4.54672724236841
N	-2.40059254595333	1.59888381644425	-2.00240935127786
C	-3.08467974269146	3.25759639143541	-3.57220497156401
C	-1.63726958829963	1.03259376786788	-2.94667952464306
C	-3.13562518951274	2.70420320808617	-2.28731200568454
C	-4.92586288728282	4.19660548474909	-1.32123261556520
C	-5.80455885078473	4.45719943850508	-0.28705419870409
C	-3.96410037653491	3.18358917446896	-1.18358428442404
C	-5.71608119426609	3.73382565638976	0.91154685529937
C	-4.70221603313683	2.79478584158912	1.06838058138454
C	-3.82261878687758	2.47323574071295	0.02744552932766
Ir	-2.51070276729158	0.93991772495571	-0.04478500577642
C	-6.26192289727329	-0.88539899412569	-1.30750651935309
C	-6.67536973582369	-1.49081948804811	-0.11265022620671
C	-4.18176300932882	-0.17741332941430	-0.24489560665454
C	-5.86366179218617	-1.44812027787362	1.00603803649960
C	-5.00722989978896	-0.28463014282644	-1.36976344478534
C	-4.63798702762926	-0.76922296439349	0.95346266640455
C	-4.02376220621296	-1.03941944744090	3.41035441749982
C	-3.17855779560437	-0.69361814757866	4.45009048506422
C	-3.80286440358974	-0.52467969227096	2.12760768448643
C	-2.11097596834922	0.16957717053664	4.20267054667116
C	-1.93590555495500	0.64442781128335	2.91604848957484
N	-2.75315187300122	0.30773164491821	1.90909856529581
H	-6.20919281320095	-1.90772588901499	1.93428609763870
C	-0.17073868379410	-2.83299782336975	-0.57407993648236
C	1.16287046631776	-2.38588018377454	-0.42721126491980
N	-0.98732113617522	-0.59938762551197	-0.24036280121608
C	1.39300298345210	-1.04827997169988	-0.17385573964865
C	-1.19021635125715	-1.90291811327696	-0.47127294511486
C	0.29669368069383	-0.13855993841036	-0.06781404954648

C	2.67906562071582	-0.42905227195770	0.01040985730747
C	2.86627972065868	0.93399449087802	0.28935065935775
C	1.75191877267119	1.84682059422231	0.39508464936822
C	1.85364768680662	3.19953818038095	0.65070532212525
C	0.46969078663717	1.25109573216206	0.20644371191775
C	0.67731967044215	3.97067896515826	0.71711449362475
C	-0.54032396720676	3.33510594410782	0.53141839361398
N	-0.65971202047940	2.02474409152535	0.29469155378050
N	4.18387772890925	1.19063313279845	0.42122063002413
N	3.92901938974514	-0.96930185293578	-0.02705368606109
C	4.83097720024150	0.03659647707850	0.22770441055189
C	6.24642468346279	-0.09080444813959	0.29006198142717
C	9.07929538639100	-0.27842654882810	0.48059852976309
C	7.04496892183483	1.05961650045987	0.64563659180408
C	6.92374543313443	-1.30472330776047	0.01438426403259
C	8.28141442383645	-1.41519996870330	0.08917037800888
C	8.42011544576034	0.94105156266455	0.74268384763515
H	6.35204022120846	-2.18525090001215	-0.28295034576201
N	10.42234699483109	-0.38164452657235	0.58114214315956
O	6.49620481912350	2.23244957426918	0.88250765577192
H	5.50601567046762	2.16611815873190	0.76825313808093
H	4.14726322484048	-1.94325123060321	-0.19085823102426
H	8.74976723078246	-2.36374407546653	-0.16265254076077
H	8.96329387634163	1.84289773324650	1.01283338403665
C	11.12075327174133	-1.60338350367154	0.20722126841121
C	11.22490387299613	0.78932347895902	0.91931613944588
H	10.75788873734669	1.30273590098380	1.76716313288968
C	11.40721492338868	1.76857561483127	-0.25103196677071
H	12.19923854063988	0.42805970729468	1.26899141603471
H	10.56804973504106	-2.47209967208666	0.58007527393927
H	12.08675273347757	-1.60711207458726	0.72787156746576
C	11.33771429793764	-1.73267230447500	-1.30428251787524
H	10.40736840686862	-1.46324203541559	-1.82873193380388
H	12.13156756519781	-1.06581255019336	-1.66285296493224
H	10.58467786358488	1.63476796416344	-0.97225847831904
H	12.33747106532320	1.59119286260357	-0.80544936926129
H	-4.70100365935483	0.18016545382735	-2.31025268236205
H	-7.65580116234956	-1.96885352613855	-0.06028793696909
C	-7.23947431788500	-0.75193992923122	-2.45097137325966
N	-8.05558790761389	0.42957612235028	-2.27219187288298
H	-7.90402549113937	-1.62790132324020	-2.46957499565134
H	-6.69066093159289	-0.73529855163995	-3.41854149600640
C	-9.38061710417794	0.40945968005535	-2.84734737643121
C	-10.23268410837116	1.48668587652290	-2.16906232420673
H	-9.84323188602953	-0.57171766762553	-2.66662447744410
H	-9.36644823015542	0.54969169013573	-3.95117949500491
C	-7.35796506736115	1.68209389129102	-2.50879163105835
C	-8.36357386093398	2.82322133451698	-2.68247054076045
H	-6.71801863903355	1.62861232059799	-3.41956242376762
H	-6.68707165111011	1.89734955301893	-1.66392680639732

O	-9.47788476292998	2.64191194118835	-1.85814279190402
H	-8.67089646537841	2.91185780563296	-3.74318632497746
H	-7.89713597023072	3.77515444317472	-2.39592903385866
H	-10.62162977486879	1.12318774055549	-1.20793073579072
H	-11.08652971869288	1.75006459978262	-2.82269927276385
H	-4.65167704632549	2.24133606097075	2.00923453491892
C	-6.81652041643998	3.86989937070346	1.93730746982833
H	-5.02342075192082	4.74972040852592	-2.25776060812582
N	-8.01750452866837	3.23353968820887	1.45431784794148
C	-7.92716759715402	1.78730616492337	1.26119065773368
C	-9.26637192206623	3.68265595787588	2.02310638147760
C	-10.41941330982249	3.10052382712216	1.22090218511264
H	-9.32161477398947	4.78057613957016	1.95450216902596
H	-9.37607092586589	3.43233827253960	3.10209801702016
O	-10.35358414043594	1.68616678564156	1.11285454734039
H	-10.38426496834174	3.51597179514036	0.20189137174472
H	-11.37926504530392	3.37481018540219	1.69124446626061
C	-9.21479674646256	1.09653831103920	1.68223902823919
H	-7.10261130856669	1.35406119208236	1.85839226086486
H	-7.72150823151029	1.54375746258808	0.20848916944799
H	-9.29619734129560	1.07738974208418	2.78668268147928
H	-9.19147389336491	0.05170412171761	1.34150153538107
H	-1.48060385299019	3.89021735014434	0.57533002371310
H	-2.23635163972781	-2.19907573796002	-0.58308246990641
H	-0.40208570949599	-3.87907867967647	-0.76592477773332
H	2.83408578775395	3.65461598265734	0.79386206451779
H	0.70900086086994	5.04185510703663	0.90935811612818
H	1.98918379551054	-3.09433135520203	-0.51099625454727
H	-4.86487973311695	-1.70988400859448	3.57976060192832
H	-3.34968769064126	-1.09123603352547	5.45228202606265
H	-1.12273797173996	1.32441149669380	2.66279117300378
H	-1.42505828958419	0.47431050696124	4.99286659506341
H	-1.08462656549588	0.14396203855429	-2.64233791457519
H	-0.92548112477102	1.03741331373113	-4.97070044158878
H	-2.25503341952589	3.10199851084992	-5.55024387968840
H	-3.67275944028454	4.14650501844128	-3.79509260465528
H	-6.60122917206068	5.19318649568537	-0.41142507658114
H	-6.46965307155956	3.45357511724551	2.91008134869831
H	-7.03864394788067	4.93536136986023	2.10562389701830
C	11.68947583072712	-3.14150561048228	-1.72339437607803
C	11.37153547941876	3.23880844562589	0.11350862173263
O	11.95868600243803	4.09292740992902	-0.48536605066494
O	12.62657045844332	-3.44449495026693	-2.40455642970842
O	10.79420816960291	-4.01613969625101	-1.23890362191606
O	10.54501133206937	3.47207821936330	1.14368553533433
C	10.97944872959924	-5.40336462473292	-1.57442065847641
H	10.46545062954868	-5.95356641471222	-0.77569660789345
H	12.05322888473191	-5.63073239791212	-1.53433086675768
C	10.40455910670488	-5.72444459383957	-2.93555351870344
H	10.94859943776326	-5.18704202062897	-3.72425590595187

H	10.49615352612853	-6.80232478577706	-3.13356713449352
H	9.33924600437790	-5.45576594506773	-2.98720336995118
C	10.26497996194343	4.84170646337146	1.48459718682318
H	10.02900098549613	4.82221803140139	2.55623598857845
H	11.17551290515314	5.43359057780059	1.32422832973000
C	9.10738053206084	5.36459221517594	0.66338601247770
H	8.84956432522218	6.38366168253850	0.98641546835612
H	9.37498034309089	5.40574540343200	-0.40206072273095
H	8.21935691875349	4.72649011547714	0.78556831167566

8. Ir-4-S$_1$

NImag=0

109

Coordinates from ORCA-job opt

C	3.38688094898701	-0.07976640721545	-4.31922519163644
C	4.22774949047248	-1.10261777228615	-4.75760514968083
N	4.16428618504477	-0.39718488451061	-2.08627656016746
C	5.02850673277579	-1.75980326585855	-3.83984738766030
C	3.38787437238232	0.23877339521678	-2.97388837699387
C	4.99097449992460	-1.39514393528646	-2.48942917248831
C	6.68342042351654	-3.05540471432232	-1.62841214956307
C	7.40668486660608	-3.57117298073894	-0.56359303410402
C	5.78947340554769	-1.99771944647100	-1.42025698642256
C	7.23595385698914	-3.02349033561830	0.70943302035400
C	6.34803662619969	-1.97203187051054	0.91704822314569
C	5.59942722846104	-1.43374822615149	-0.13706944911362
Ir	4.21817084668411	0.03386641858589	-0.06580613880651
C	7.29420319209463	2.88619140905736	-1.27167656504488
C	7.68859495193140	3.40449522274540	-0.03658541246357
C	5.69371103581934	1.40174315712886	-0.19601290680449
C	7.09506948090872	2.92705937252573	1.12227755002464
C	6.31410620116224	1.90094073793916	-1.34815240215721
C	6.10831207245083	1.93630715463650	1.04633320424491
C	5.70001491857761	1.71536288957980	3.55188330184952
C	4.99860969004504	1.10228817963765	4.57553257221686
C	5.43402404405700	1.37802911795566	2.22022687520672
C	4.02739950805362	0.15160606577841	4.26228909854892
C	3.80338488627113	-0.14249930424288	2.93014118976131
N	4.48410763156232	0.45026978752014	1.94009738303769
H	7.40605332443556	3.33184059831428	2.08740923423755
C	1.53947228132828	3.61312027652420	-0.17501046527624
C	0.25815767997270	3.03577871256827	-0.02103151540195
N	2.56093772385235	1.44117853669929	-0.08131539173553
C	0.15698914030265	1.66537773032900	0.11094705713642
C	2.63891624081404	2.77363059799051	-0.19609451416860
C	1.33037788436871	0.85095618513475	0.08779779023985
C	-1.06260543589841	0.92123421744775	0.27874479328853
C	-1.12442473158249	-0.47350775072146	0.42133228061271
C	0.06692133605375	-1.29003377230327	0.39653344664171
C	0.08779529630728	-2.66490446074909	0.51569422332212

C	1.28683033543793	-0.56892467448871	0.22655581928558
C	1.32551328449743	-3.33418773172360	0.46479976831205
C	2.47746975825646	-2.58067209803804	0.30473637021222
N	2.47945847412937	-1.24764138605160	0.19710516576730
N	-2.41076027409561	-0.85573174905947	0.56348218155754
N	-2.35296538627661	1.35256816601551	0.33577125917933
C	-3.15802801646298	0.25199927996864	0.50844595579710
C	-4.57713205559459	0.25227645614611	0.60543193507040
C	-7.41620085363227	0.16959657517800	0.76816045245356
C	-5.27307167387051	-0.99688828732294	0.80740333930836
C	-5.35542540634488	1.43145512478248	0.49470233214767
C	-6.71761921529604	1.41553236400861	0.56599666384203
C	-6.65426960734291	-1.01183848135165	0.88661388785565
H	-4.86151568694837	2.39100662575339	0.33394294080475
N	-8.76544695402761	0.13922612791894	0.82317444391285
O	-4.62338466371463	-2.13749072992647	0.91088325771001
H	-3.64332833939871	-1.97350102643760	0.81018173109309
H	-2.65765583853852	2.31473928149029	0.27071377535458
H	-7.26431512430509	2.34819826568620	0.44790234861567
H	-7.11900704994521	-1.98488066345943	1.02149505181900
C	-9.55836659653360	1.34205966148365	0.61008481601637
C	-9.46714806795076	-1.13680508397199	0.92324375767403
H	-8.99999382011556	-1.73741741872719	1.71176274488293
C	-9.48928714181818	-1.92599652921373	-0.39590383649931
H	-10.48916723690798	-0.92246197470470	1.25669345493338
H	-9.10700537069917	2.17896084035125	1.15309629461589
H	-10.54282551854670	1.17521803270979	1.06489066491662
C	-9.71627203064994	1.70664423681424	-0.87034446614581
H	-8.75238376203326	1.56256891377249	-1.38422641406261
H	-10.45765935737809	1.07522553111354	-1.37562788439489
H	-8.66998221754801	-1.57757715265134	-1.04536975671505
H	-10.41662205775951	-1.77027315967421	-0.96190001153664
H	6.03389910480162	1.51188945215825	-2.32953324657518
H	8.45898332039502	4.17538046825904	0.01847198053070
H	6.24345306547479	-1.56236584676575	1.92402841084259
H	6.82022663075242	-3.48258289382129	-2.62376830900891
H	3.46058429757007	-3.05617592676077	0.26507972929939
H	3.64881415850388	3.17357144812513	-0.31648678785823
H	1.67087197867074	4.68883978443969	-0.27571472028574
H	-0.84523535651439	-3.21438463548592	0.64357096654886
H	1.39087878910480	-4.41776148983957	0.54880072436354
H	-0.62955834880722	3.67090032752518	-0.00525315746976
H	6.46644520315408	2.45619090738308	3.77382698231010
H	5.20824621018359	1.35949722166926	5.61553003316985
H	3.06349945550169	-0.88168339495759	2.62354106075362
H	3.45409697889337	-0.35936651184148	5.03566803807127
H	2.75758020351683	1.03215189525322	-2.57234713126484
H	2.74216611153678	0.46665964836792	-5.00743467080026
H	4.25842228996573	-1.38224602958407	-5.81237333843963
H	5.69537885630406	-2.55860094088068	-4.16022411190309

H	8.10503084234817	-4.39462716443206	-0.72172261726531
C	-10.10964450486300	3.14975569946879	-1.08990438014539
C	-9.28123642152595	-3.42243321573886	-0.27863682370749
O	-9.70129593711241	-4.21810936593381	-1.06835915141153
O	-10.97182764409017	3.52265604094013	-1.83224875894047
O	-9.33714631782185	3.96993820629524	-0.35965784374782
O	-8.51132717270669	-3.73806786467269	0.77242445348685
C	-9.56621304119221	5.38426666414131	-0.49464322501155
H	-9.18878132616673	5.81766040658474	0.44052480914980
H	-10.64892979693806	5.55655219845404	-0.55975453444322
C	-8.85127641520391	5.94680809568107	-1.70241065433101
H	-9.25932114946654	5.52088594343634	-2.62918303929891
H	-8.98690939090470	7.03741244938504	-1.74116091526499
H	-7.77241320795804	5.73734375467873	-1.65554887513311
C	-8.08242896961730	-5.10558005927212	0.90424005283832
H	-7.91498733643036	-5.24402652722609	1.97999748543991
H	-8.90098551031819	-5.76005459170422	0.57724545876884
C	-6.82221393322524	-5.34691972940358	0.10336238476527
H	-6.46012695403640	-6.37066016350609	0.27712790617887
H	-7.01940044001668	-5.23649571775277	-0.97237944925587
H	-6.02891054683244	-4.64321650460978	0.39661165714376
H	7.80850486080530	-3.42254167515397	1.55023236075485
H	7.76333681920022	3.25511922437764	-2.18699576081752

9. Ir-1-T$_1$

NImag=0

123

Coordinates from ORCA-job opt

C	-1.91777993055190	1.64927342192816	-4.52904283384550
C	-2.70108101730430	2.76913829194641	-4.80437489672449
N	-2.72986653155651	1.62885212703224	-2.28634007510806
C	-3.49586985673370	3.30300730946527	-3.80226402781728
C	-1.96088353533526	1.11202446826818	-3.25474492531560
C	-3.50316851091083	2.71642360179251	-2.53397277680111
C	-5.30802301787161	4.13960189625286	-1.48353715783606
C	-6.14218864156525	4.37923444409874	-0.40380791336659
C	-4.31774704938249	3.15086731882020	-1.39551689391862
C	-5.98261257540156	3.65400022227292	0.78596336146657
C	-4.94283940438597	2.73159441814312	0.88663430246171
C	-4.10854455449432	2.43331233364521	-0.19834595689097
Ir	-2.79247035421989	0.91175172655337	-0.34646179346556
C	-6.56318655424943	-0.89509217667082	-1.59307629862387
C	-6.93060036339250	-1.57361444393966	-0.42214605870490
C	-4.46618178037468	-0.20356356336969	-0.54985770748036
C	-6.09183136477047	-1.56487657950343	0.67984836295586
C	-5.32365156233563	-0.26293276554056	-1.65612602942481
C	-4.88277631726275	-0.85616791225142	0.63050711351252
C	-4.22329647178706	-1.20209867401573	3.06584405053295
C	-3.36732358260333	-0.87493977121262	4.10561856113576
C	-4.02769856864567	-0.64050135723403	1.80138506973757

C	-2.32077186203755	0.01605528240607	3.87215330629459
C	-2.17123974575847	0.53790856467434	2.59943936927560
N	-2.99730583271564	0.21772500877867	1.59358858143752
H	-6.40309232873342	-2.07356729383702	1.59455324345982
C	-0.49580447829403	-2.89552286676266	-0.89163781085067
C	0.80156541930039	-2.50976321031057	-0.69796622307895
N	-1.28582345399837	-0.64926180069821	-0.57762005694295
C	1.09674100037953	-1.14494102697018	-0.43104617279246
C	-1.52691708420944	-1.92124959337546	-0.82419708817481
C	-0.01116600074154	-0.21895386792651	-0.36959161421939
C	2.36169206179349	-0.60219927613609	-0.21533559063811
C	2.57448063140065	0.80317081225112	0.04881142991391
C	1.47190067506519	1.72826855022529	0.10618211284345
C	1.59826743025188	3.09177158292061	0.34801859245402
C	0.17455196359722	1.18200620165343	-0.09495155928018
C	0.43764531595059	3.88817149008099	0.38600366836150
C	-0.78818974413040	3.29133015396876	0.19786638420305
N	-0.92527386556026	1.96387400364192	-0.02668617399185
N	3.87352088305223	1.04076861379131	0.22661271662133
N	3.60968440854640	-1.14991106644434	-0.18363629310747
C	4.52194617847718	-0.13617461614556	0.09514031562377
C	5.91449328959511	-0.29561488850115	0.24386684765662
C	8.73274015741361	-0.58041122181258	0.61256641526952
C	6.74234597421844	0.83959656935367	0.55863174917325
C	6.56207051084290	-1.55407121629120	0.10867927866759
C	7.91210207179574	-1.70195920363101	0.28067079037729
C	8.10625941238433	0.68191675220068	0.73182452248364
H	5.97155943899768	-2.43742396794265	-0.13932706369660
N	10.07755656708046	-0.72944198742639	0.81953704710234
O	6.22333723034933	2.05728180715262	0.69132204340077
H	5.23912493498511	2.00696754616912	0.55456466093977
H	3.83397091406608	-2.12914206318458	-0.31887653357495
H	8.34362060452674	-2.69286473616640	0.16162712232409
H	8.66848560615573	1.58345949951656	0.96370965818512
C	10.72865046626451	-2.01746510980484	0.65043352916349
C	10.91226841031417	0.40689045048733	1.16767299606918
H	10.38326894171793	1.05982154216747	1.87472615514303
C	11.34249722202000	1.21648854670120	-0.05010470771947
H	11.80432331732179	0.03290410369820	1.68616422066154
H	10.11945822562939	-2.81569990862158	1.09592905141308
H	11.67515283527149	-1.99750552809051	1.20498034217336
C	11.01090900102908	-2.34526882051944	-0.81116275074184
H	10.09343165568865	-2.34823597634781	-1.41174541545762
Cl	11.73345531382749	-3.98324054376464	-0.93472937765432
H	11.72437508741585	-1.64063259772401	-1.25423782718204
H	10.48013545644650	1.58279333805616	-0.61971552501145
H	11.98977491098834	0.63360317878013	-0.71561392029506
Cl	12.28173800895598	2.65178854329794	0.47819340211635
H	-5.05945867196883	0.26663164529988	-2.57545065494653
H	-7.90103774805482	-2.07226452154410	-0.36881676273074

C	-7.58281357393685	-0.71883858464995	-2.69222055726354
N	-8.58859755239692	0.24322983388635	-2.27854979037959
H	-8.09278068112352	-1.67385606912916	-2.88950320715467
H	-7.07592072150419	-0.41160363475977	-3.63202404401816
C	-9.82826274894782	0.22697637772349	-3.02191167221626
C	-10.90457459000719	0.92067851239658	-2.17399179745179
H	-10.13262025248805	-0.81075987533530	-3.21925199748022
H	-9.72384883958449	0.72380572570230	-4.01126632071071
C	-8.08684922495238	1.60185630411515	-2.15907790282060
C	-9.25259810509884	2.56530841766249	-1.87377319514215
H	-7.56637014315067	1.92460789748805	-3.08824969439598
H	-7.34476521653533	1.64716008565663	-1.35100055741667
O	-10.34513072285550	1.88334973661782	-1.30159132941224
H	-9.57185357209343	3.07652150122192	-2.80111385906924
H	-8.94262130552624	3.32763914251871	-1.14861253425774
H	-11.41456787349479	0.18836133585189	-1.53164496291939
H	-11.66202976035273	1.38818124571766	-2.83138780306255
H	-4.83132075755807	2.17771745930972	1.82256510471518
C	-7.01150041436267	3.77495579562375	1.88166977451282
H	-5.45829694314503	4.69525251257897	-2.41148954805974
N	-8.24475776205136	3.08283864484183	1.53043885254632
C	-8.13843615172796	1.62294547879077	1.42630468899332
C	-9.36405407524301	3.48973959963995	2.37226885342949
C	-10.47626616160023	2.46377189876966	2.28657501026173
H	-9.73936585941831	4.47718533338612	2.05581590451023
H	-9.06401879970328	3.58686544936942	3.43726753629625
O	-10.07488358867887	1.21414774591549	2.82533399126413
H	-10.78430123611051	2.34783636434587	1.23133581541946
H	-11.34890246940506	2.80323249262456	2.86345463981030
C	-8.69749294811607	0.92090185523939	2.64965848408706
H	-7.09203388997817	1.32944636996835	1.27782090339037
H	-8.69139254652737	1.27004973822290	0.54249016487305
H	-8.12784601615662	1.21477587542723	3.55160264486117
H	-8.59837407458649	-0.17005476488468	2.53820043572635
H	-1.71618754224738	3.86424012479119	0.22599633825502
H	-2.57144777991697	-2.19982830738424	-0.97603466380831
H	-0.75698386458031	-3.93295914754913	-1.09787518548013
H	2.58562511117602	3.52766749181543	0.50277664337193
H	0.49784489939532	4.96109515052015	0.56579179103048
H	1.61067862023840	-3.24116917814251	-0.74744819330924
H	-5.04984950627921	-1.89306656771321	3.22631904217013
H	-3.51539650828143	-1.31003615811514	5.09586743560344
H	-1.37121695953268	1.23973779423187	2.36429806989433
H	-1.62680007775986	0.30616449188293	4.66105776883696
H	-1.36760316312497	0.23727493077667	-2.98818869612531
H	-1.27975363573473	1.19449268570622	-5.28688810756817
H	-2.69165624344664	3.22286631265228	-5.79728543623107
H	-4.11553319655516	4.17797164955967	-3.99513153649528
H	-6.94866238875546	5.11147845864453	-0.48604908195783
H	-6.58753103101964	3.40646945514923	2.83984135951152

H -7.26696032693640 4.83517379754466 2.03265951363523

10. Ir-2-T$_1$
NImag=0
91
Coordinates from ORCA-job opt

C	-2.99366593840525	1.95546002417860	-3.65872575588103
C	-3.87290768514372	3.03078314612799	-3.53112959478047
N	-3.51285583981002	1.26947401153853	-1.43425008967056
C	-4.56403399181037	3.20381546724107	-2.34441658540574
C	-2.84408382445059	1.09992298437296	-2.58344092324033
C	-4.37887126056973	2.30433603230383	-1.28915146553597
C	-5.96204593821775	3.36251565761508	0.36738276087178
C	-6.57211510070945	3.33342466133290	1.61266110847341
C	-5.06113569046832	2.35312252056993	0.00608895301518
C	-6.28264278225859	2.29025904423354	2.49390499547068
C	-5.38749998087391	1.28591630001610	2.13574616429326
C	-4.75140725244067	1.29255015032526	0.88889804750505
Ir	-3.36361516682005	0.00412347340239	0.19364352861263
C	-6.50131175114795	-2.15419027815680	-1.91977297210024
C	-6.76336881380057	-3.19984009574748	-1.03291497073539
C	-4.83092222733810	-1.23018409543430	-0.41475386205368
C	-6.06948428548237	-3.26603228810124	0.16596775394775
C	-5.55075992426416	-1.18444328519000	-1.61388100491207
C	-5.11324049171659	-2.29120571195950	0.47572245304393
C	-4.46961687620853	-3.20222640737888	2.76586468055300
C	-3.69014932702752	-3.07245038790168	3.90188966362393
C	-4.34101135469347	-2.28288619759799	1.71910573613454
C	-2.77884855088120	-2.01989989707443	3.98708091114303
C	-2.68925149157028	-1.14393036806919	2.92197812991123
N	-3.44382310343779	-1.26995377773680	1.82169632922252
H	-6.27894232345527	-4.08256847054417	0.85948448187359
C	-0.70210028587814	-3.07976999596246	-1.62522873867790
C	0.58054172898634	-2.61169569061567	-1.34598275820395
N	-1.69090888140354	-1.17024909957425	-0.54239932538511
C	0.71247373770275	-1.37413475152227	-0.63349052654856
C	-1.81131181092466	-2.36465021175365	-1.22674583879153
C	-0.48794755143270	-0.69825159423735	-0.25067192777884
C	1.90518031802391	-0.75772285237059	-0.27502959122501
C	1.99428880454506	0.50040430271000	0.44143455243842
C	0.81167282744603	1.18440121007964	0.82859857084194
C	0.77671401351946	2.41102006431618	1.52282129332305
C	-0.42251322929744	0.57409274755386	0.48892858744284
C	-0.44293822114536	2.96525140848686	1.84719680242654
C	-1.61901941271021	2.29470013538379	1.48769706758754
N	-1.59778427895535	1.13453210384079	0.83363799568947
N	3.28201472269767	0.82209247040452	0.63506264076669
N	3.21658199629811	-1.12295545217785	-0.47841496973836
C	4.02774505211676	-0.14971249133429	0.08694727495299
C	5.44224531226760	-0.16502371185276	0.09817543070616

C	8.29560947967455	-0.16453178182609	0.17995971501036
C	6.16619439342900	0.89676463818794	0.74364161445574
C	6.20393722577395	-1.19833943904913	-0.50477105452148
C	7.57029293284579	-1.21210054343123	-0.47786072579819
C	7.55414737454890	0.87743457032488	0.76882821437548
H	5.69465773247880	-2.01763265186424	-1.01625989887903
N	9.65904406231242	-0.18861871962899	0.23572189336301
O	5.54584124664238	1.90679664924637	1.32293548820747
H	4.56503843915760	1.78759070178035	1.22692800834030
H	3.53408861895827	-1.96912356963470	-0.92789960171436
H	8.09363636703983	-2.03172151859638	-0.96342741984257
H	8.02975606120369	1.71575366789660	1.27119781239354
C	10.42440874349784	-1.24955427244532	-0.40012336184812
C	10.39742729058297	0.87872972941672	0.89239294344697
H	9.89053494284749	1.17108143907373	1.82184547440419
C	10.58606634297617	2.10909085591208	0.01076956454250
H	11.38112140288203	0.49030595022332	1.18630610631319
H	9.94892087051410	-2.22357293688041	-0.22290550966921
H	11.40965296120909	-1.30024338219690	0.08075089923280
C	10.60709784060607	-1.04025574495663	-1.89994417945433
H	9.64100044529170	-0.90787532864246	-2.40376616771647
Cl	11.40895580517295	-2.46133583938700	-2.61524686120055
H	11.22897428401206	-0.16092892192098	-2.11055026727939
H	9.62329441404903	2.49305902069289	-0.34973313503191
H	11.21848235977133	1.88702646954266	-0.85830438105486
Cl	11.37947622586615	3.40209715435941	0.94445968466331
H	-5.37502679487949	-0.37731519041126	-2.32751301201085
H	-7.51040646082783	-3.95658626764880	-1.27714182147560
H	-5.18890794799479	0.48063128721702	2.84602518441265
H	-6.19301437664009	4.17783383582683	-0.32084757557273
H	-2.60596887145813	2.69272574654349	1.73154708811185
H	-2.82933956961236	-2.69509055581832	-1.42421442050140
H	-0.84254309964996	-4.01798110600813	-2.16311375226068
H	1.71289042892800	2.90190665562637	1.79152419083257
H	-0.50894551828742	3.91336405354932	2.38142472183527
H	1.45917471340830	-3.17315594201106	-1.66022921592176
H	-5.19146766237607	-4.01290537356028	2.68131705267990
H	-3.79256423311978	-3.78505009256574	4.72236127224472
H	-1.99963902131504	-0.30001565240671	2.93745819163436
H	-2.14942888420233	-1.87600195910523	4.86498659660643
H	-2.17882773578859	0.23728896417974	-2.62667602365217
H	-2.43511386015782	1.77779729035732	-4.57744647437151
H	-4.02056027418654	3.72744667905199	-4.35839354497054
H	-5.26144976559594	4.03174462549492	-2.22735547361680
H	-7.27515757092423	4.11766496339317	1.89763413114878
H	-6.76649468952809	2.25987001041242	3.47298415418952
H	-7.05057702799018	-2.09405042205381	-2.86223423728682

11. Ir-3-T_1

NImag=0

141
Coordinates from ORCA-job opt

C	-1.38851820468678	1.86495339675683	-4.05411455292702
C	-2.12145171417688	3.02520067642546	-4.30511657127535
N	-2.31436510187008	1.75486576446442	-1.85787301401310
C	-2.94824812238524	3.53010037949551	-3.31741692395337
C	-1.51495326718058	1.26552419110565	-2.81527428154219
C	-3.04260169999181	2.87856202503214	-2.08207515582542
C	-4.87196324297784	4.28842649503457	-1.06413552396956
C	-5.78670977369974	4.46363686410461	-0.04326674552990
C	-3.90970322406352	3.27063638237108	-0.97405103891381
C	-5.73357866351507	3.65025185856148	1.09805068113238
C	-4.71853896600493	2.70686518889542	1.21653178322203
C	-3.80588856430790	2.47024626244408	0.18259262519652
Ir	-2.49098708164742	0.94876796125507	0.04012912419450
C	-6.16880219898704	-0.80645284209924	-1.50294500886216
C	-6.62110079245882	-1.50534914381804	-0.37540578393748
C	-4.14584063584603	-0.15755748685422	-0.30442825927650
C	-5.85755182401760	-1.53653306080528	0.77744012027198
C	-4.92038609091911	-0.19016254597253	-1.46811045179338
C	-4.64199391920887	-0.83977154932595	0.82770158057651
C	-4.13391831624709	-1.26510035049367	3.28699600850985
C	-3.33895278157536	-0.98105492610821	4.38353218081804
C	-3.85998012829607	-0.66915969228776	2.05079108269107
C	-2.26796396387027	-0.09870159720399	4.24011399123716
C	-2.03867817003546	0.45719406609303	2.99542367830952
N	-2.80760448734920	0.18029117864279	1.93345861341880
H	-6.23431141344582	-2.06850312344965	1.65324600382354
C	-0.12490644122852	-2.80689781855062	-0.60221338502372
C	1.16753933475892	-2.39189671262137	-0.39942977709862
N	-0.96171710408491	-0.59240578727207	-0.19398428100944
C	1.43431650673669	-1.04523528856514	-0.07587987490479
C	-1.17192584311375	-1.85413293775640	-0.49126851237633
C	0.31174733907743	-0.13713001329924	0.03931850297913
C	2.69485863277591	-0.47249549395302	0.14708698018146
C	2.87599003587370	0.91164016078521	0.47085307971367
C	1.75224915098152	1.81017663148148	0.60830253545725
C	1.85146602938997	3.15235454029299	0.93210441855015
C	0.46595017086463	1.24047341259199	0.38438939424268
C	0.67192747420429	3.92352276028595	1.02859783861235
C	-0.53955878855595	3.31227394574383	0.80400118427148
N	-0.65329794516961	2.00161191014221	0.49838094606179
N	4.17639541719074	1.17985985006409	0.60916128626195
N	3.95706799488870	-0.98771506309884	0.11422800588971
C	4.85477896254440	0.03803051046445	0.39461370950252
C	6.25941432396911	-0.07421537997460	0.44053063592063
C	9.10802203351338	-0.21995016013166	0.49648657177409
C	7.06151699174429	1.09732642046019	0.70379587912541
C	6.94735081331855	-1.29857394574009	0.21761962688013
C	8.31038253735933	-1.38747016948143	0.23958682500390

C	8.44026826963587	1.00407289077373	0.72259485683394
H	6.37735643907547	-2.20779231251460	0.01531996997331
N	10.46415232899377	-0.28666631218861	0.50627970123146
O	6.50807491844342	2.27975095538226	0.91727656440610
H	5.51936317589561	2.19554559892082	0.86921475271935
H	4.20357887962248	-1.94493828260224	-0.09538690196743
H	8.77834683479974	-2.34975010679954	0.04243258237615
H	8.98128630028464	1.92871638350296	0.90522332939916
C	11.15667145486587	-1.52658656384887	0.19448300486728
C	11.25420126353421	0.93316558939927	0.62822186976290
H	10.87391191550558	1.52412004293796	1.46966883824802
C	11.26369456036845	1.78779500255069	-0.65024555985072
H	12.27633322159067	0.63678259644898	0.89395014773612
H	10.66109650821618	-2.36114706487339	0.70323177677743
H	12.16595652578052	-1.46325959602995	0.62250033914355
C	11.24290641632034	-1.81567375133935	-1.30907877745939
H	10.26539409832962	-1.60766774746333	-1.77284105520526
H	11.98868384805062	-1.18398945876802	-1.80806718811937
H	10.38941717899980	1.52531861045959	-1.26804498590520
H	12.14689985026809	1.60042788275155	-1.27473803614221
H	-4.58156969394520	0.34530485049009	-2.35829139469485
H	-7.59546230402128	-1.99760580028429	-0.40027063963079
C	-7.10061952557403	-0.58791848179147	-2.67077172645351
N	-7.91632012913500	0.58380189694742	-2.43700786367637
H	-7.76780904855001	-1.45548628671779	-2.77608556996930
H	-6.51496655365834	-0.50692188701309	-3.61324177947785
C	-9.21793209068197	0.61755695621940	-3.06338957683438
C	-10.09136049464779	1.64395964878459	-2.33585563413546
H	-9.69084676743097	-0.37152266920716	-2.97781122122993
H	-9.15929444779818	0.84325248777589	-4.15126078357139
C	-7.20500244552871	1.84603823439606	-2.54761199739723
C	-8.19884929472903	3.00381363941292	-2.67156411925545
H	-6.52843220551291	1.85919941215016	-3.43314498669132
H	-6.56894177933284	1.99144459256487	-1.66183281267778
O	-9.34418852345337	2.76644350764874	-1.90686258132669
H	-8.46408847200587	3.17603044754554	-3.73326386090362
H	-7.74052752875263	3.92758909490098	-2.29428885441574
H	-10.51671938618193	1.20777260869349	-1.42161825986584
H	-10.91857141632052	1.96329695017995	-2.99843916331926
H	-4.69591456188067	2.08315700792349	2.11310333662140
C	-6.87144108057387	3.69389639675213	2.09050606173565
H	-4.94238092114943	4.91212171991615	-1.95767140583592
N	-8.03958331229618	3.07295991707789	1.51698302856568
C	-7.91068395722895	1.64936915194961	1.21457480576042
C	-9.31993460396398	3.45391325489444	2.06525820227959
C	-10.42480551798810	2.92435694388069	1.16501501217849
H	-9.39260977493399	4.55259472878537	2.08592166370689
H	-9.47147512439125	3.11151477632699	3.11317862384997
O	-10.32484897137046	1.52558458561107	0.93972808947701
H	-10.35252852667681	3.42655927820193	0.18785769552098

H -11.41000060097129 3.14093900350405 1.61214288308876
C -9.20406222441557 0.90712622253368 1.51318556461051
H -7.10891660598955 1.18164986822754 1.81737419915768
H -7.64767250323358 1.49539314725884 0.15745723634046
H -9.33711935276383 0.79466097941344 2.60684878538814
H -9.14509044795242 -0.10512446695865 1.08888801849126
H -1.48129495566881 3.86126332634461 0.86388933726479
H -2.21326613541384 -2.14235363613176 -0.65707920998608
H -0.36259706298578 -3.83981268477106 -0.85076304237714
H 2.83034711764182 3.59881749112296 1.10503452857975
H 0.71021745408645 4.98341635714908 1.27518961072256
H 1.98813724843772 -3.10752968961269 -0.48955870346775
H -4.97748250939321 -1.94777095088617 3.37653296972437
H -3.55234105227938 -1.44135560283035 5.35002092099692
H -1.22027087464418 1.15627938403388 2.82297424862360
H -1.62115372030555 0.15895992548683 5.07839211114230
H -0.96850245911028 0.35648910125195 -2.56435217071957
H -0.73042320178149 1.42900499866095 -4.80531619235286
H -2.04764990989841 3.52980061651824 -5.27020318234110
H -3.53152544683441 4.43288335922401 -3.49167536665693
H -6.58439502092392 5.20274674257594 -0.13808480180410
H -6.55094950485557 3.21413288783857 3.04289031275450
H -7.12049001409736 4.74026737295829 2.32624022459875
C 11.57547294935983 -3.25562563683242 -1.62197866719329
C 11.16118012323033 3.28688141007451 -0.46065282699889
O 11.58789481626896 4.09060783768936 -1.24001095105720
O 12.41651246895167 -3.61869927490886 -2.39439680647400
O 10.78112942587443 -4.09052049490697 -0.93178940293810
O 10.47103972592577 3.60632094279306 0.64406412632369
C 10.96199492501614 -5.50007374940517 -1.14762938315401
H 10.57894408544771 -5.97358363955590 -0.23422465731289
H 12.03764690217051 -5.70489398507056 -1.23485967231181
C 10.21784540051848 -5.97308631925470 -2.37662054889160
H 10.63188945635726 -5.51093262740785 -3.28319780714970
H 10.31628850413102 -7.06410038629483 -2.47553705266778
H 9.14721509297320 -5.73025526607320 -2.30725951483131
C 10.13746992094021 4.98910813490452 0.85217895490048
H 10.04105507307539 5.09395393210256 1.94058798140491
H 10.97435763523072 5.60733922264224 0.50131751127437
C 8.84969917276539 5.34017528292445 0.13934661463127
H 8.56151601201242 6.37505713173559 0.37601931632588
H 8.97775817447563 5.26594291698866 -0.94997589466502
H 8.03352011148625 4.67084141009536 0.44975549024962

12. Ir-4-T_1

NImag=0

109

Coordinates from ORCA-job opt

C 3.41952095375478 -0.13825481478284 -4.32859585604344

C	4.25677914689954	-1.17202648931824	-4.74794438368923
N	4.18610796322398	-0.42641626241160	-2.08779935577009
C	5.04971624150572	-1.81992324381905	-3.81712185726268
C	3.41679681824507	0.19994857071565	-2.98846137898795
C	5.00829240720718	-1.43506669037104	-2.47266547981158
C	6.68264969381242	-3.09581228755760	-1.57763307870422
C	7.39363086004812	-3.60249844457105	-0.50026624271813
C	5.79650051879170	-2.02787026938659	-1.39097724308928
C	7.21875626952930	-3.03587366301917	0.76354710401880
C	6.33878518001624	-1.97373038296344	0.95043636742250
C	5.60368231238867	-1.44542551925240	-0.11718163578526
Ir	4.23232124349207	0.02834868932614	-0.07146239731928
C	7.30945568592049	2.87417032730566	-1.29308781492448
C	7.69757543254631	3.40482450325277	-0.06159886405969
C	5.71096421375861	1.39469552699239	-0.20908478735150
C	7.10210017364571	2.93612440835657	1.09998259966701
C	6.33274702807247	1.88465677539864	-1.36380893403274
C	6.11907448517916	1.94145144695714	1.02983337141513
C	5.70949777371468	1.73491735034518	3.53649641172952
C	5.00859723567373	1.12635081743546	4.56324774908882
C	5.44332370464862	1.39077104906453	2.20692733414889
C	4.03767170045310	0.17356392451193	4.25550790846589
C	3.81296627249857	-0.12713546489105	2.92519639127783
N	4.49313431357221	0.46175034308693	1.93210025551257
H	7.40871304990126	3.35089456564799	2.06211506752116
C	1.54589559867524	3.62131490723606	-0.18473583629402
C	0.29683756547755	3.07048810971072	-0.03447590086558
N	2.57618504114197	1.45236591891563	-0.09241621905689
C	0.15288292775372	1.67543922266447	0.09886270556974
C	2.67091029919358	2.75724007325870	-0.20956577667155
C	1.34866758198908	0.85799402136920	0.07459920719301
C	-1.05164779732278	0.97401237848080	0.25982635785197
C	-1.11304584350637	-0.44958731310182	0.39915098297898
C	0.08195987995010	-1.26249953933362	0.37261865748996
C	0.09537680985359	-2.64111378964322	0.48909490770902
C	1.31340169461809	-0.56216676735538	0.21068713444875
C	1.33278139231501	-3.32108058426430	0.44349594819760
C	2.48629339685561	-2.58723306369741	0.29312254916286
N	2.49206760918273	-1.24074581004098	0.18787175807757
N	-2.38286554221096	-0.83838015918056	0.54253559218380
N	-2.34974651938678	1.38466171616833	0.32464486679461
C	-3.15416414823164	0.26336640963439	0.49760243646629
C	-4.56044853476016	0.25945885515280	0.60223545686819
C	-7.40627900782591	0.18003629823156	0.78705195873450
C	-5.26065372300222	-0.98774526637603	0.79741083091613
C	-5.34661222362973	1.44057442784202	0.50809941627987
C	-6.70994226710992	1.42163405984014	0.58954504692347
C	-6.64005938289515	-1.00267301124076	0.88685567850716
H	-4.85638012108699	2.40385177083879	0.35233502455477
N	-8.76158242147367	0.14325768657446	0.85974346276272

O	-4.61146525654205	-2.13694671556575	0.88669764603890
H	-3.63690268049065	-1.97570452925686	0.78496128212359
H	-2.67517618069014	2.33958793353862	0.26755936182951
H	-7.25622322605061	2.35678790353811	0.48513741020961
H	-7.10197120953793	-1.97777460698499	1.01754641849093
C	-9.55664660880058	1.34226248063711	0.64625052887662
C	-9.45402903058696	-1.13702242276293	0.94640050025838
H	-8.98134821524331	-1.74648201396399	1.72538433207918
C	-9.48098291444114	-1.91244166114339	-0.38142147609554
H	-10.47665439615578	-0.93480677088582	1.28800135438124
H	-9.10940745677078	2.18163202210323	1.19065140723360
H	-10.54285852225099	1.17440109015469	1.09871453874797
C	-9.71553383707960	1.71091863294396	-0.83397281500111
H	-8.74370578873244	1.59573989595315	-1.34034117225491
H	-10.43521344594543	1.05987247909507	-1.34619573524630
H	-8.65554425971615	-1.56220087493209	-1.02253652879691
H	-10.40471191835744	-1.74207474874851	-0.94957823336476
H	6.05772591940944	1.48493861491117	-2.34231223180667
H	8.46515213435945	4.17865367347107	-0.01103164840128
H	6.23093159113584	-1.54939951088165	1.95066924811152
H	6.82277585342608	-3.53797690466008	-2.56576459006158
H	3.46775280401772	-3.06400231116590	0.25747515841866
H	3.68072075913404	3.15693535792306	-0.33424438996699
H	1.69083457976571	4.69533772697600	-0.28681138944754
H	-0.84106990319887	-3.18491837392391	0.61069197629516
H	1.38382644417027	-4.40558190702850	0.52622142193691
H	-0.58350949987567	3.71726233019093	-0.01743079297436
H	6.47640382305958	2.47622434596222	3.75484271268208
H	5.21879190647063	1.38840724998952	5.60180212410580
H	3.07379910417521	-0.86895948601199	2.62302503102954
H	3.46553972509800	-0.33403443069598	5.03179953537729
H	2.78796919741070	1.00321986669907	-2.60469951886007
H	2.78059721929578	0.40103704927678	-5.02753848315000
H	4.29037428375964	-1.46734262097718	-5.79819504889147
H	5.71323054367653	-2.62718488813339	-4.12263944327307
H	8.08590266720653	-4.43386383082052	-0.64173943952424
C	-10.15000763526626	3.14085068756891	-1.05424011196149
C	-9.28351269566477	-3.41089664595505	-0.28726145834833
O	-9.72004441050547	-4.19599706995090	-1.07989867255275
O	-11.03040447720398	3.49028807037861	-1.78809091159712
O	-9.39538113166820	3.98633238211381	-0.33299231648193
O	-8.49830405724040	-3.74710767079897	0.74706409680651
C	-9.66999864522999	5.39097494312804	-0.46611936783603
H	-9.29908703558363	5.83750133439734	0.46569742289706
H	-10.75801894214164	5.53045393610773	-0.52351644512143
C	-8.98279327505434	5.97780863029733	-1.67907103683225
H	-9.38467025989722	5.53978833018814	-2.60294437587680
H	-9.15287073171122	7.06387927511889	-1.71532867446688
H	-7.89757989505638	5.80174390456600	-1.64124895421977
C	-8.07095440854921	-5.11584480887955	0.84870649785618

H	-7.89169034350419	-5.27593835233630	1.91975776032896
H	-8.89287785772794	-5.76438935078960	0.51778887152222
C	-6.81841165112098	-5.34446312054824	0.03107280640091
H	-6.45438973212545	-6.37126474028816	0.18313441526421
H	-7.02677741383224	-5.21510326096797	-1.04054263210280
H	-6.02286320804560	-4.64464650139896	0.32767694594454
H	7.78163647926060	-3.42789038772454	1.61389522071431
H	7.78068399970035	3.23610261321620	-2.20994211894857
